# Genetic diversity in global chicken breeds in relation to their genetic distances to wild populations

**DOI:** 10.1186/s12711-021-00628-z

**Published:** 2021-04-14

**Authors:** Dorcus Kholofelo Malomane, Steffen Weigend, Armin Otto Schmitt, Annett Weigend, Christian Reimer, Henner Simianer

**Affiliations:** 1 Animal Breeding and Genetics Group, Department of Animal Sciences, University of Goettingen, Goettingen, Germany; 2 Center for Integrated Breeding Research, Department of Animal Sciences, University of Goettingen, Goettingen, Germany; 3 Institute of Farm Animal Genetics, Friedrich-Loeffler-Institut, Neustadt, Germany; 4 Breeding Informatics Group, Department of Animal Sciences, Georg August-Universität Goettingen, Goettingen, Germany

## Abstract

**Background:**

Migration of a population from its founder population is expected to cause a reduction of its genetic diversity and facilitates differentiation between the population and its founder population, as predicted by the theory of genetic isolation by distance. Consistent with that theory, a model of expansion from a single founder predicts that patterns of genetic diversity in populations can be explained well by their geographic expansion from their founders, which is correlated with genetic differentiation.

**Methods:**

To investigate this in chicken, we estimated the relationship between the genetic diversity of 160 domesticated chicken populations and their genetic distances to wild chicken populations.

**Results:**

Our results show a strong inverse relationship, i.e. 88.6% of the variation in the overall genetic diversity of domesticated chicken populations was explained by their genetic distance to the wild populations. We also investigated whether the patterns of genetic diversity of different types of single nucleotide polymorphisms (SNPs) and genes are similar to that of the overall genome. Among the SNP classes, the non-synonymous SNPs deviated most from the overall genome. However, genetic distance to the wild chicken still explained more variation in domesticated chicken diversity across all SNP classes, which ranged from 83.0 to 89.3%.

**Conclusions:**

Genetic distance between domesticated chicken populations and their wild relatives can predict the genetic diversity of the domesticated populations. On the one hand, genes with little genetic variation across populations, regardless of the genetic distance to the wild population, are associated with major functions such as brain development. Changes in such genes may be detrimental to the species. On the other hand, genetic diversity seems to change at a faster rate within genes that are associated with e.g. protein transport and protein and lipid metabolic processes. In general, such genes may be flexible to changes according to the populations’ needs. These results contribute to the knowledge of the evolutionary patterns of different functional genomic regions in the chicken.

**Supplementary Information:**

The online version contains supplementary material available at 10.1186/s12711-021-00628-z.

## Background

Domesticated chickens (*Gallus gallus domesticus*) are one of the most widely distributed domestic animal species in the world. This is in part because of their portability and flexibility of transportation through human migration, stock trading, and expansion in the agricultural practices [1, 2], and in part because their use for nutrition does not suffer from any religious or cultural reservations. It is commonly accepted that the current world-wide chicken populations originate predominantly from the domestication of the red jungle fowl (*Gallus gallus* species) in Asia (reviewed by [3]). From the centers of domestication, chickens have dispersed into different parts of the world. New breeds or lines have been formed as populations moved outward from ancestral territories and settled in new colonies. One expectation from such expansion processes is the increase in genetic distances (increased differentiation) of the outward populations to the original ancestors, which is expected to be associated with a loss of genetic diversity within such populations due to genetic drift and subsequent serial founder effects [4–6]. Previously, we studied the overall genetic diversity between and within chicken breeds [7]. In the current study, our aim was to investigate whether the observed genetic diversity in the chicken breeds that we analyzed in [7] resulted from their genetic expansion from their wild type populations following the theory of genetic isolation by distance [8–10] and the model of expansion from a single location such as the ‘Out of Africa’ migration model [4]. The theory of genetic isolation by distance is based on population genetic patterns and assumes that genetic differentiation increases as geographic distances between populations increases. This is because the exchange of genetic material between populations (i.e. mating opportunities) is limited by geographic distance [8, 11]. Likewise, movements of individuals away from their founders are expected to increase genetic differentiation. This has been established with the ‘Out of Africa’ theory, which asserts that modern humans originated from Africa [12] and that the genetic diversity of human populations world-wide decreases as the geographic distance from east Africa (Ethiopia) increases [4, 5, 13, 14]. Similar studies in cattle also reported that the genetic diversity of cattle populations decreases as the geographic distance to their domestication center in Southwest Asia increases [15, 16].

The loss of genetic diversity within the migrated populations that can be explained by the geographic distance from their founders, is believed to be a good measure of neutral genetic diversity as a consequence of genetic drift. However, the overall genetic diversity is also the result of population-specific events such as mutations, natural selection that favors adaptation in the current environments, and/or artificial selection (e.g. in livestock production practices), as well as population specific drift [5]. The consequences of selection are often measured by non-neutral genetic variation because it is assumed that non-neutral genomic regions with functional fitness effects evolve differently from neutral genomic regions. In this study, we used the global representative collection of chicken breeds described in [7] to investigate the pattern of the overall genetic diversity as the genetic distance from the centers of chicken domestication increases, given all the events that occurred in the genome. In addition, we investigated whether the patterns of the genetic diversity of different functional regions of the genome were similar to those of the overall genome. We hypothesized that changes in genetic diversity may be faster for some genes or single nucleotide polymorphism (SNP) classes, depending on their functions, and that changes may also differ between breeds or breed groups due to different adaptive or artificial selection targets. Therefore, the patterns of the relationship between genetic diversity and genetic distance in various functional regions may behave differently from the overall pattern due to differences in selection patterns, in addition to other population-specific events.

Studying the theory of genetic isolation by distance and/or the concept of migration from a single location by using chicken populations poses some challenges because the physical locations of chicken populations do not always represent their geographic origins (following migration from founders). For many chicken breeds, the time point when they migrated to their current locations is unknown. We also believe that, unlike humans, for which genetic evolution is mostly driven by natural circumstances, e.g. rapid migration, geographic distance may not be the best predictor of the genetic diversity for chicken populations because crossbreeding forced by man, refined breeding programs, and artificial selection for desired traits have largely shaped the evolution of domesticated chickens. The changes in genetic diversity and evolutionary rates are often rapid in domesticated livestock and the genetic architecture of chickens around the same geographic location may also differ greatly depending on the breeding practices or selection targets. Thus, instead of geographic distances, we used Reynolds’ genetic distances [17], which estimate genetic differences under the assumptions that genetic differentiation occurs by genetic drift. However, we followed similar concepts as used in the genetic isolation by distance theory and in the model of expansion from a single founder [5, 8, 9].

## Methods

### Data description and quality control

The data consisted of 3002 individuals from 162 chicken populations collected in Asia, Africa, South America, and Europe. The populations were classified into nine breed categories, which were based on their continent of origin and/or type, as described in Additional file 1: Table S1. The chickens were genotyped with the 600 K Affymetrix® Axiom™ Genome-Wide Chicken Genotyping Array [18]. We used only the SNPs from the 27 autosomal chromosomes and removed 499 SNPs with ambiguous chromosome annotations. SNPs from chromosome 16 were also removed due to incorrect annotations. The data were filtered using the SNP & Variation Suite (SVS) version 8.1 [19] based on an animal call rate of ≥ 95% and a SNP call rate of ≥ 99%. We performed linkage disequilibrium (LD) based pruning to account for ascertainment bias [20] using the PLINK software v1.9 [21, 22] with the parameters *indep 50 5 2*. After the filtering steps, 156,753 SNPs were retained for further analysis. Imputation was performed to recover missing genotypes using Beagle 3.3 [23]. A more complete description of the data is in Malomane et al. [7].

### Classification of the SNPs

We classified SNPs according to their functional consequences and assigned them to their associated genes using the Affymetrix Galgal5 annotation map [24]. SNPs were classified into the following categories: non-synonymous which included missense and nonsense (only eight) variants, synonymous, exonic (a combination of the non-synonymous and synonymous SNPs as well as other coding and non-coding exonic SNPs that were not assigned as non-synonymous or synonymous), intronic, 5′ untranslated region (5′UTR), 3′ untranslated region (3′UTR), upstream, downstream, and intergenic classes. SNP assignments were prioritized in the order shown in Table 1. For example, if one SNP is associated with two genes but has different functional consequences for each of these genes (e.g. non-synonymous for one gene and synonymous for the other gene), then a non-synonymous functional consequence was considered prior to other consequences, followed by synonymous and so forth. As for the up- and downstream variants, a SNP was assigned to the upstream class if it was located within 5 kb upstream of the gene and similarly for the downstream SNPs. The distribution of SNPs into their functional classes is in column 2 of Table 1.

**Table 1:** Comparison of the linear relationship between observed heterozygosity and genetic distances of populations to *Gallus gallus* ssp. for different classes of SNPs

SNP class	Number of SNPs	R^2^	Slope	SE of slope
Overall SNPs	156,753	0.886	− 0.706	0.010
Non-synonymous	1082	0.880	− 0.645	0.009
Synonymous	3891	0.893	− 0.708	0.010
Exonic	5959	0.891	− 0.694	0.009
Intronic	71,175	0.888	− 0.711	0.010
5′UTR	118	0.830	− 0.674	0.012
3′UTR	1383	0.875	− 0.686	0.010
Upstream	11,559	0.881	− 0.710	0.010
Downstream	8777	0.883	− 0.707	0.010
Intergenic	57,782	0.884	− 0.701	0.010

The number of exonic SNPs is the sum of non-synonymous and synonymous SNPs plus the coding and non-coding exonic SNPs, which were assigned to neither the non-synonymous nor the synonymous classes. All R^2^ values are significant, p < 0.001. *SE * standard error

To assign SNPs to individual genes, the 156 K SNPs were mapped to 10,456 associated genes [24].

### Estimation of genetic diversity outward from wild populations

Two subspecies of the wild red jungle fowl (RJF) populations, *G. gallus spadiceus* and *G. gallus gallus,* which were sampled about 20 years ago, were used as the reference for original founders and were assumed to reflect the genetic diversity in the centers of domestication.

We estimated the pairwise Reynolds’ genetic distances [17] between the two wild populations (*G. gallus* ssp.) and the domesticated populations (*G. gallus domesticus*), and then calculated the mean genetic distance of each domesticated population to the two wild populations. Observed heterozygosity (${\mathrm{H}}_{\mathrm{o}}$) was also estimated for each population. Then, we estimated the linear relationship between the overall genetic diversity (as measured by ${\mathrm{H}}_{\mathrm{o}}$) within the domesticated populations and their mean genetic distances to the two wild populations. The amount of variation in ${\mathrm{H}}_{\mathrm{o}}$ within the populations that could be explained by the genetic distance was measured by the R^2^ value of the linear model. To investigate if the patterns of genetic diversity for the different classes of SNPs and genes were similar to those of the overall genome (when using all SNPs), we also estimated ${\mathrm{H}}_{\mathrm{o}}$ for each SNP class and gene, and subsequently estimated its linear relationship with the genetic distances of the populations to the wild populations.

Because some genes were annotated with only one or very few associated SNPs and some were annotated with more SNPs, we considered only genes with at least 10 associated SNPs (i.e. 6303 genes) for comparisons with the overall pattern. Then, we evaluated the rate of change in genetic diversity within the genes due to the change in genetic distances of populations to the wild populations using the regression coefficient of the linear relationship between the two parameters (${\mathrm{H}}_{\mathrm{o}}$ and genetic distance to wild populations).

### Functional annotation of genes

Genes with the 5% lowest and 5% highest regression coefficients for the relationship between genetic diversity within populations and genetic distance to the wild populations were grouped into functional terms using the ClueGO (v2.5.7) [25] ontology enrichment package in Cytoscape (v3.8.0) [26]. In addition, individual gene functions were annotated using the DAVID functional annotation tool (v6.8) [27].

## Results and discussion

### Relationship between overall genetic diversity and genetic distance to the wild populations

**Fig. 1:**
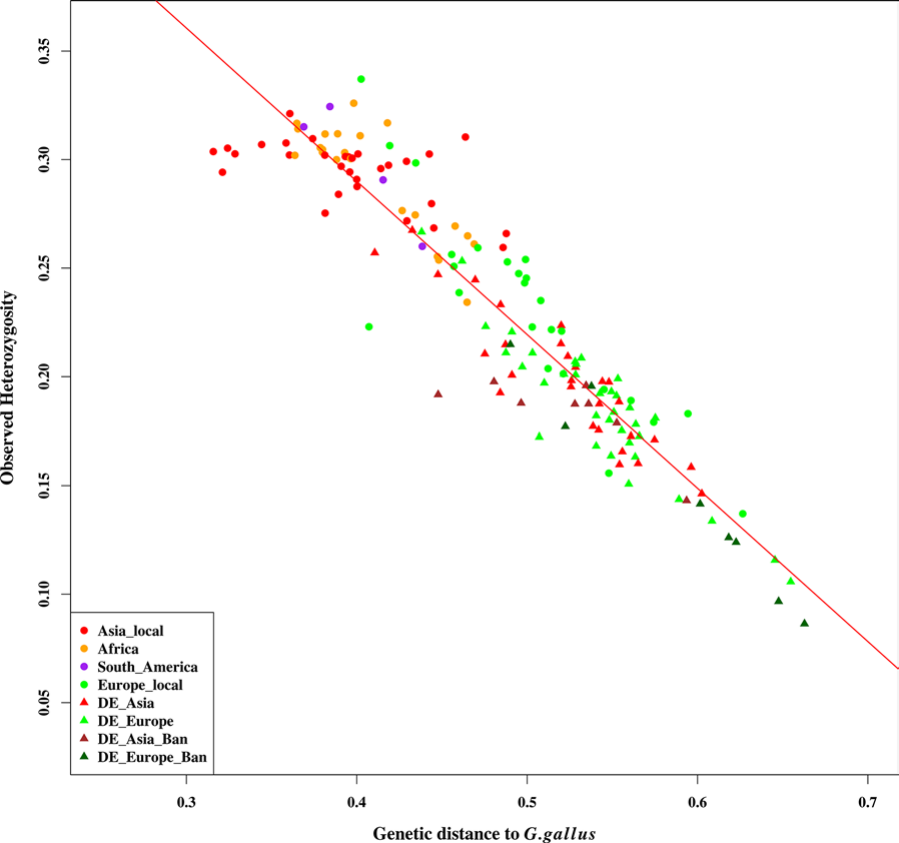
Relationship between heterozygosity within populations and their genetic distances to *Gallus gallus*. The full names of the categories and description are in Additional file 1: Table S1). The different breed categories are represented by symbols of different colors and shapes. The fitted regression line, with the equation heterozygosity = 0.572–0.706 × (genetic distance to *G. gallus*), is drawn in red. The R^2^ for the linear regression was 0.886 (p < 0.001)

A strong inverse relationship was found between the genetic diversity (${\mathrm{H}}_{\mathrm{o}}$) within populations and their genetic distances to the wild populations (*Gallus gallus*), as shown in Fig. 1. This relationship was similar even when using only neutral markers (intergenic SNPs, see Fig. 3). Across these chicken populations, 88.6% (Table 1) of the total variation in ${\mathrm{H}}_{\mathrm{o}}$ was explained by the genetic distance to the wild populations, which is slightly higher than the percentage obtained in several human studies based on geographic distances. Geographic distances of humans out of Africa explained 76.3% of the microsatellite heterozygosity and 78.4% of the variation in fixation index (${F}_{ST}$) in [5] and explained 85% of the microsatellite heterozygosity in [14]. In [28], geographic distances of humans out of Africa had a correlation of − 0.91 with SNP haplotype heterozygosity and of − 0.87 with microsatellite heterozygosity. Studies in humans have also shown that there is a high correlation (e.g. from 0.77 to 0.89 [5]) between genetic distance (using different genetic distance measures) and geographic distance. However, correlations reported for domesticated cattle were not as high, e.g. 0.62 in [29] and 0.75 and 0.54 in [15] for ancient and modern cattle samples, respectively. It has been suggested that the weaker relationship between geographic and genetic distance in modern domesticated cattle is, among other reasons, due to the human manipulation of genetic diversity, as is the case for many domesticated livestock [15].

Since we had different sample sizes, with some populations having less than 15 sampled individuals, we checked whether this affected the estimates. We estimated ${\mathrm{H}}_{\mathrm{o}}$ when only populations of 15 or more individuals were considered and found that number of individuals did not affect the estimates. We also sampled 1000 SNPs in 100 replicates to validate that the relationship between ${\mathrm{H}}_{\mathrm{o}}$ and genetic distance was not due to chance. The percentages of variation explained in the 100 replicates ranged from 86.1 to 88.9% with a mean of 87.9%. Additional file 2: Figure S1 shows the regression plots of the 100 replicates with their 95% confidence intervals. We also used ${F}_{\mathrm{ST}}$ as an alternative measure of differentiation, and found a Mantel correlation coefficient of 0.97 between pairwise ${F}_{\mathrm{ST}}$ values and the corresponding Reynolds’ distances. Reynolds’ genetic distances of populations to the wild populations (*G. gallus*) and the ${F}_{\mathrm{ST}}$ values were highly correlated, with a Pearson’s correlation coefficient of 0.99 and their relationship is shown in Additional file 2: Figure S2 with an R^2^ value of 0.99. When using ${F}_{\mathrm{ST}}$, the genetic differentiation of the breeds from the wild populations (*G. gallus*) explained 88.5% of the variation in ${\mathrm{H}}_{\mathrm{o}}$ (see Additional file 2: Figure S2).

Since heterozygosity and genetic distance are not entirely independent of each other, in the next steps, we investigated the possibility of a structural link behind the observed relationship. First, we permuted the SNPs to ensure that the decreasing heterozygosity was not an artefact of the Reynolds’ distance and found that there was nearly no relationship between ${\mathrm{H}}_{\mathrm{o}}$ and genetic distance based on permutated SNPs (R^2^ = 0.01). Second, we estimated the regression of ${\mathrm{H}}_{\mathrm{o}}$ on genetic distance when starting from each of the 160 populations (this time not from the two wild populations). Then, we investigated whether the R^2^ value of the linear regression was directly associated with the ${\mathrm{H}}_{\mathrm{o}}$ within the populations, i.e. whether populations with higher ${\mathrm{H}}_{\mathrm{o}}$ automatically resulted in higher R^2^ values and vice versa. We found that although there was some association between the populations’ ${\mathrm{H}}_{\mathrm{o}}$ and R^2^ values (Pearson’s correlation of 0.35), it was not very strong. R^2^ values for the regression of ${\mathrm{H}}_{\mathrm{o}}$ on the genetic distances for the populations from Asia (Asian fancy and local populations) ranged from 0.71 to 0.91 and those on the genetic distances for the other populations of non-Asian origin ranged from 0.57 to 0.90. Of the 160 populations, 29 yielded higher R^2^ values than the two wild populations, of which 25 of them are of Asian origin and only four (Albanian Crowers (ALxx), Hungarian Yellow (YH), Schweizer Huhn (SCw) and African Kuroiler (KUR)) are of non-Asian origin, based on our classification (3 European and 1 African). However, the four were all clustered with Asian populations [7]. Furthermore, KUR is believed to have originated from South Asia (India) and arrived recently in Africa. More information on these populations can be found in [7]. Generally, higher explanatory power of the populations’ ${\mathrm{H}}_{\mathrm{o}}$ lies with the Asian populations. A human-based study that used geographic distance as the explanatory factor of heterozygosity reported that, although some locations outside of Africa had reasonably high R^2^ values (e.g. 0.74), none of them were higher than the regression based on geographic distance from Ethiopia (the possible founder origin) with an R^2^ = 0.76. However, other locations in Africa had R^2^ values higher than Ethiopia (e.g. R^2^ as high as 0.87) [5]. Therefore, our results do not contradict those found in the literature. Nonetheless, one of the shortfalls with the *Gallus gallus* subspecies that we included in our study is that the samples were not from naturally existing populations in the wild. Instead, they were sampled from small populations that have been isolated for years, therefore they may have a reduced genetic diversity, which reduces their explanatory power of the genetic diversity (${\mathrm{H}}_{\mathrm{o}}$) within the domesticated chickens.

**Fig. 2:**
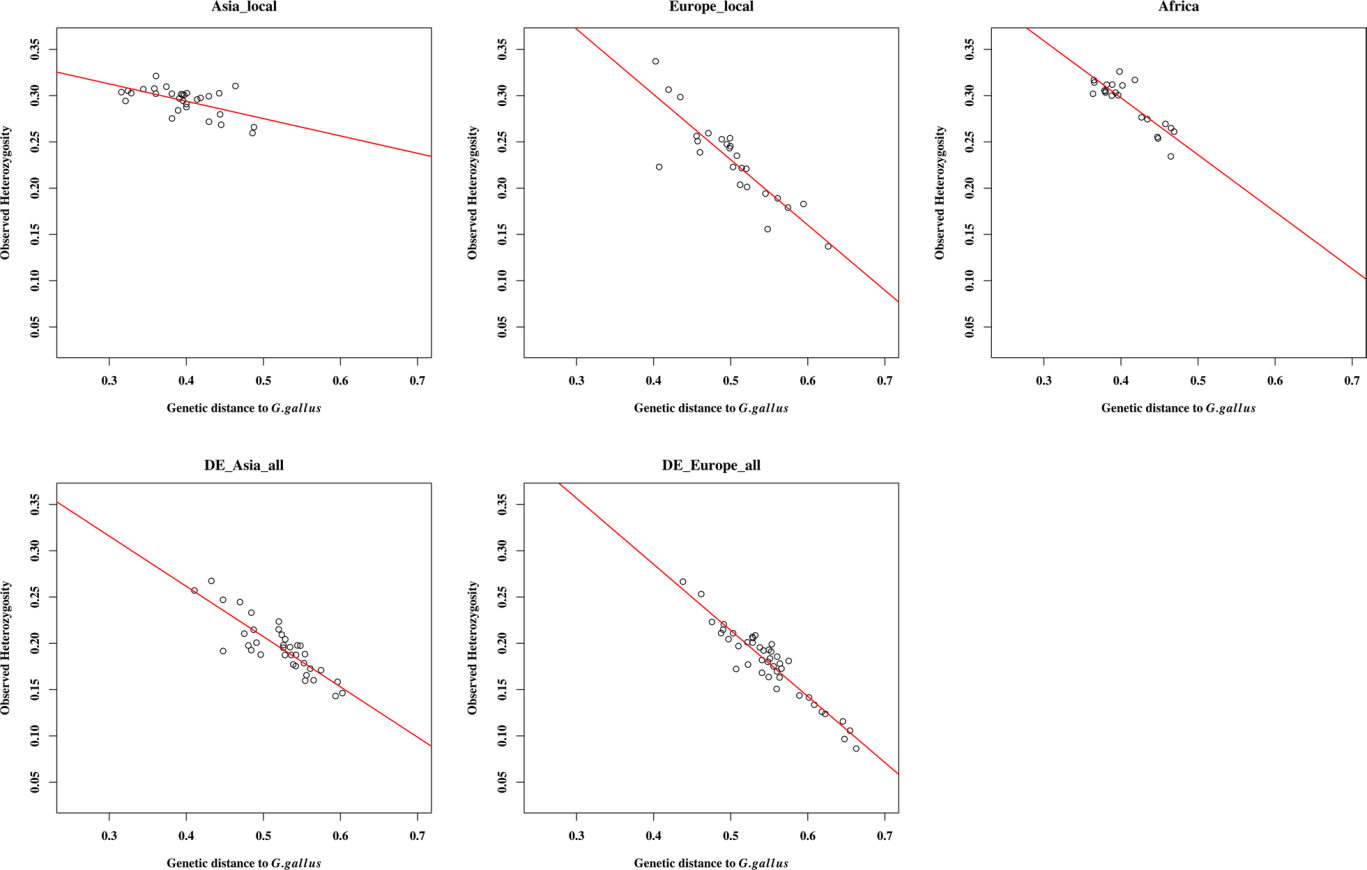
Comparison of the relationship between heterozygosity and genetic distance to *Gallus gallus* for different groups of populations. DE_Asia_all and DE_Europe_all consist of fancy breeds, including the bantamised breeds of Asian and European backgrounds, respectively

Since we studied populations with different histories, including local and fancy populations from different management and breeding backgrounds, we also investigated whether the groups of populations from different backgrounds resulted in different patterns of genetic diversity. Figure 2 shows that the association between ${\mathrm{H}}_{\mathrm{o}}$ and the genetic distances of populations to the wild populations (*G. gallus*) was weaker within the Asia_local group than within the rest of the other groups, see Additional file 1: Table S2 for R^2^ values and regression coefficients. The South_America category was not included in this analysis since it consisted of only four populations. Genetic distances of the Asian local populations to the wild populations were generally shorter than those of the other groups, in particular the European breeds and Asian fancy breeds. Not only is intercrossing common among Asian local breeds, which promotes high genetic diversity, but there is also a high probability of gene flow between the wild populations (RJF) and local chickens in Asia, as reported in some studies e.g. [30]. This gene flow limits variations in genetic diversity due to genetic drift. However, the Asian fancy populations have been separated from the wild populations for quite some time, the same applies for the European and African breeds, thus the probability of exchange of genetic material with the wild populations is very low for these populations [31]. Consequently, the fancy breeds (both Asian and European), the European local, and the African categories all show a high association of the ${\mathrm{H}}_{\mathrm{o}}$ within each of these populations with their genetic distances to the wild populations. Figure 2 clearly shows that the groups of fancy breeds comply with the concept of genetic isolation by genetic distance; however, in these breeds the rate of change in ${\mathrm{H}}_{\mathrm{o}}$ due to genetic distance to the wild *G. gallus* is more rapid compared to their respective local populations (see Additional file 1: Table S2). In addition to the historic separation from the wild populations, the effects of drift are also strong within the fancy categories as a result of other factors, such as small effective population size and consequent inbreeding.

Based on our results, we can conclude that the variance in ${\mathrm{H}}_{\mathrm{o}}$ within the domesticated chicken populations can be explained well by genetic distance to the wild populations (*G gallus*). Although our results may not directly prove this, because of the lack of geographic sampling coordinates, given the whole dataset (Fig. 1), it is clear that geographic distance alone may not predict well the observed genetic variations in the chickens for two reasons, as described in the following.

(i) Breeds of the same geographic origin are scattered across the genetic diversity spectrum, in particular, the European (green symbols) and Asian (red symbols) type breeds, as shown in Fig. 1 and highlighted in [7]. The European chickens sampled from the German fancy breeders (denoted with prefix DE_) have a much reduced genetic diversity and their genetic distances to the wild populations (*G.gallus*) are much larger than those of their respective local breeds. However, when considering the sampling areas, the genetic diversity may correlate to the geographic distances to the *G. gallus* wild chicken within the Asian breed categories. Presumably, many of the fancy breeds originated from a small number of breeding birds that were imported from Asia to Europe, after which they were subjected to strong phenotypic selection, with small effective population sizes, population bottlenecks, and intensional inbreeding to keep the desired traits. These practices are likely responsible for most of the differences in the genetic diversity of the fancy Asian and European type breeds vs. their respective local types.
(ii) The concept of isolation by distance assumes that individuals from nearby locations are likely related because mating between them is possible. This is often the case for traditional breeding systems but not for breeding and management practices for fancy breeds. In fancy breeds, gene flow between small stocks may occur based on personal contacts or personal relationships between breeders, but is not related to geographic distance forming a subpopulation structure within the breed. Actually, such gene flow between fancy breeds is also very limited. Furthermore, if geographic distance was a better predictor for the loss of genetic diversity and increased differentiation of domesticated breeds to the wild populations, then the African and South American breeds would be expected to have a much reduced genetic diversity due to their geographic distances and to show large genetic distances to the wild populations and to the rest of the Asian populations. However, we found that both these expectations were not fulfilled and that some of the African populations were clustered with the wild type populations [7].

Therefore, the observed differences in genetic diversity between domesticated breeds may not be predicted by geographic expansion only, but rather by a combination with other aspects or subsequent events, such as effective population size, type of breeding practices, and possibly subsequent series of founder events following the geographic expansion, as previously suggested [5, 6]. Such events that took place after the geographic expansion have definitely contributed to the differences in allele frequencies between populations and thus to the genetic distances between the domestic chickens and the wild populations. In addition, equilibrium between genetic drift, migration, and mutation was probably not reached in all the studied populations, which would be compatible with the theory of genetic isolation by distance [5, 8, 9]. The theoretical expansion models are also based on ‘natural’ expansion through migration, while chickens and other livestock were actively transported by humans (e.g. with ships) to distant places.

### Comparisons of the patterns of heterozygosity between the overall genome and different functional SNP classes

**Fig. 3:**
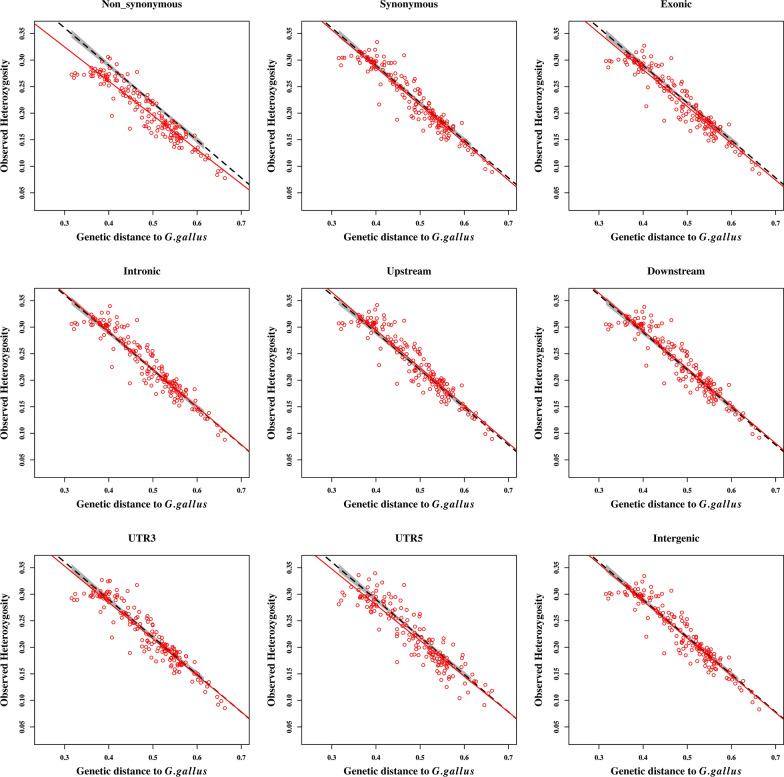
Heterozygosity within populations estimated from different SNP classes vs. Reynolds’ genetic distances of populations to the *Gallus gallus* ssp. The red circles represent the 160 domesticated populations for the corresponding SNP class. Dashed black lines represent the regression lines for the relationship between observed heterozygosity and the genetic distance to *G. gallus* for the overall pattern and the red lines are for the SNP classes. The areas shaded in gray represent a 95% confidence interval. The R^2^ values and slopes of the linear relationships are in Table 1. UTR5 and UTR3 refer to the 5′ and 3′UTR SNP classes, respectively

We compared the patterns of the relationship between ${\mathrm{H}}_{\mathrm{o}}$ and genetic distances of populations to the wild *Gallus gallus* obtained by using the whole set of SNPs to those obtained by using different SNP classes, as shown in Fig. 3 and Table 1. The rate of change in ${\mathrm{H}}_{\mathrm{o}}$ associated with the genetic distance to the wild populations is represented by the slope in column 4 of Table 1. Among the SNP classes, the class of non-synonymous SNPs showed a relevant deviation from the overall pattern, i.e. the ${\mathrm{H}}_{\mathrm{o}}$ across breeds was lower for this class of SNPs than for the whole genome and had the most deviating slope (− 0.645 compared to − 0.706 for all SNPs). To investigate if this different pattern of the non-synonymous SNP class was not due to the smaller number of SNPs, we resampled the same number of SNPs as in this class (1082 SNPs) from the overall set (156 K SNPs) 100 times. We estimated the ${\mathrm{H}}_{\mathrm{o}}$ for each sample and compared these with the H_o_ for the set of non-sysnonymous SNPs in Additional file 2: Figure S3, which shows that the original difference was not due to sample size.

The intergenic and intronic classes had the highest proportion of SNPs among all SNP classes (Table 1). In order to validate that the similarities of these two classes to the overall genome (whole set of SNPs) are not an artifact of the number of SNPs, we sampled 1000 SNPs 100 times from the intergenic and intronic classes (separately). Then, we estimated ${\mathrm{H}}_{\mathrm{o}}$ and compared the results to the whole set of SNPs and found that the observed similarities are not due to larger numbers of SNPs (see Additional file 2: Figures S4 and S5). All SNP classes showed a reduction in ${\mathrm{H}}_{\mathrm{o}}$ across populations as genetic distance to the wild type chickens increased, with R^2^ values ranging from 83.0 to 89.3%. The results indicated that 89.3% and 88.0% of the variation in ${\mathrm{H}}_{\mathrm{o}}$ across populations was explained by their genetic distance to the wild *G. gallus* for the synonymous and non-synonymous SNPs, respectively, while this percentage was lowest (83.0%) for the class of 5′UTR SNPs. However, it is important to note that the 5′UTR class included only 118 SNPs, which could explain the differences. To test this, we sampled 118 random SNPs in 100 replicates from the overall set and estimated the relationship, as we have done for the non-synonymous SNPs. The R^2^ for the 100 replicates ranged from 76.0 to 86.6%, with a mean of 82.8%, which suggests that the result for the 5′UTR class is most likely an artifact due to the small number of SNPs.

**Fig. 4:**
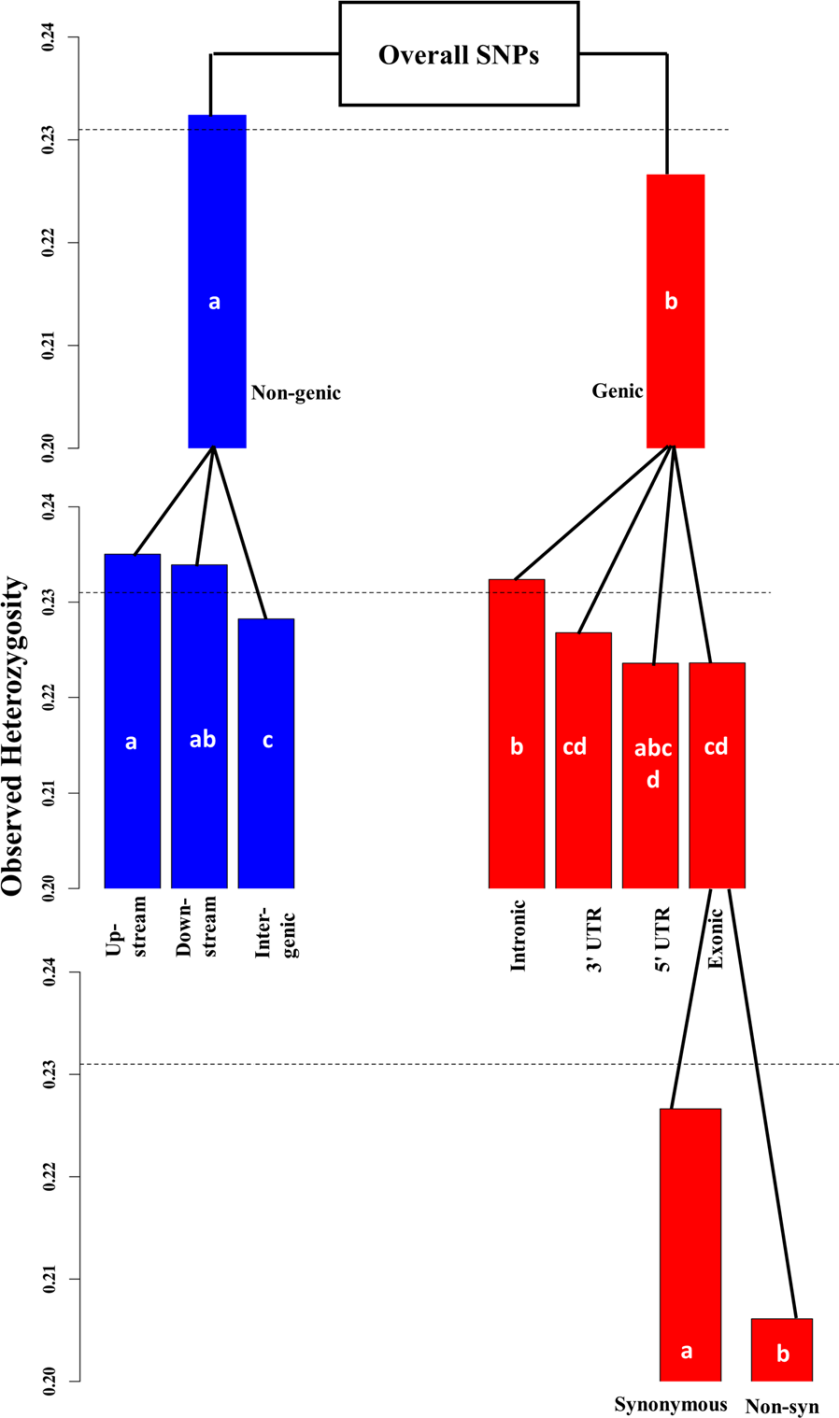
Mean heterozygosity in different SNP classes. The gray dotted lines represent the overall mean observed heterozygosity based on all genotyped SNPs. Non-syn stands for Non-synonymous. The mean heterozygosities of the SNP classes differed significantly from the overall mean (Welch two sample t-test p < 0.05), except for the 3′UTR and 5′UTR classes. Standard errors (SE) of the means were lower than 0.004 for the overall SNPs and for all different SNP classes, except for the 5′UTR class, which had SE = 0.009. Bars with different letters represent a significant difference in the mean heterozygosity within the same level, e.g. difference between ‘Non-genic’ and ‘Genic’ classes on the first level or difference between ‘Non-synonymous’ and ‘Synonymous’ classes on the third level

Figure 4 shows the mean ${\mathrm{H}}_{\mathrm{o}}$ for the different SNP classes. Generally, the ${\mathrm{H}}_{\mathrm{o}}$ was lower in the genic than in the non-genic SNP class. Within the genic class, a lower ${\mathrm{H}}_{\mathrm{o}}$ was observed in exonic than in intronic SNPs. Consistent with the results in Fig. 3, the non-synonymous SNPs presented the lowest ${\mathrm{H}}_{\mathrm{o}}$ among all SNP classes. This was expected since non-synonymous changes can present favourable or disadvantagous consequences. The theoretical assumption is that selection acts rapidly towards fixation of favourable alleles and purging of non-favourable alleles, thus leading to more homozygosity in these protein altering variants. The classes of exonic and 5′UTR SNPs followed the non-synonymous class with the lowest mean ${\mathrm{H}}_{\mathrm{o}}$. UTR variants play a role in the regulation of gene expression and translation. For example, 3′UTR variants can interfere with microRNA to facilitate the translation of critical disease genes (e.g. cancer genes in humans) [32, 33]. It is also claimed that positive selection for the adaptation of humans in different habitats has been achieved with high differentiation in the 5′UTR gene variants [34]. Such examples highlight the importance of UTR variants as possible targets for selection.

### Patterns of observed heterozygosity in genes

We investigated the patterns of ${\mathrm{H}}_{\mathrm{o}}$ in the 6303 chicken genes to which at least 10 SNPs were mapped and compared them to the overall ${\mathrm{H}}_{\mathrm{o}}$ pattern (see Additional file 3: Table S3), in order to determine whether the decrease in ${\mathrm{H}}_{\mathrm{o}}$ is faster or slower in certain genes. Coefficients of determination (R^2^) from the linear regression of the genetic distance from the wild ancestor on ${\mathrm{H}}_{\mathrm{o}}$ for each gene ranged from 0.04 to 0.73, with a mean R^2^ of 0.47, and the regression coefficients (slopes) ranged from − 0.11 to − 1.19. However, the R^2^ values were correlated with the number of SNPs genotyped within the gene, with a correlation of 0.57. The regression coefficients were independent of the number of SNPs within genes, with a correlation of 0.03. The correlation between the regression coefficients and R^2^ values was − 0.52.

We evaluated the regression coefficients of the relationship between ${\mathrm{H}}_{\mathrm{o}}$ and genetic distance for the genes with the 5% highest and lowest regression coefficients, i.e. 32 genes at each end. Regression coefficients ranged from − 0.11 to − 0.34 for the 5% of genes with the lowest regression coefficients and from − 0.99 to − 1.19 for the 5% of genes with the highest regression coefficients (see Additional file 1: Table S4 and Additional files 4 and 5 for the top and lowest 5% ranges, respectively). The genes in the top 5% showed rapid changes in ${\mathrm{H}}_{\mathrm{o}}$ with genetic distance of the breeds to the *G. gallus* wild chicken and those in the lowest 5% showed very slow changes in ${\mathrm{H}}_{\mathrm{o}}$ with genetic distance. We used the DAVID annotation platform [27] to identify the function of each gene in the lowest and top ranges (see Additional file 1: Table S4). In addition, using the ClueGo package [25], functional annotations of the genes were obtained for the combination of molecular function, biological, and immune system processes, as well as KEGG pathways. Functions of the individual genes in the top 5% range included transmembrane transport, protein transport and protein metabolic processes, and lipid metabolic processes among other functions. However, none of these top 5% genes formed any functional cluster. Most of the genes in the lowest 5% range had consistently lower H_o_ across the breeds, regardless of the genetic distance to the *G. gallus* wild chicken (see Additional file 5), and they were mainly related to critical functions for normal functioning of the individuals. Common functions among the individual genes in the lower 5% range included brain morphogenesis and development, axon development, positive regulation of cell proliferation, positive regulation of reactive oxygen species metabolic process, regulation of cell death, cell and structure morphogenesis, salivary gland morphogenesis, and lung morphogenesis, among other functions. However, functional classification revealed only three functional clusters, namely: brain development (*EGFR*, *PAFAH1B1*, *PTPRS* and *RTN4*), regulation of axon extension (*DPYSL2*, *PTPRS* and *RTN4*), and morphogenesis of salivary gland (*EGFR*, *ESRP2* and *FGFR1*).

The consistent lower genetic diversity (${\mathrm{H}}_{\mathrm{o}}$) for genes with the lowest 5% regression coefficients and the limited or lack of relationship of ${\mathrm{H}}_{\mathrm{o}}$ with genetic distance to *G. gallus* can be the result of several factors, as described in the following.

(i) On the one hand, some genes may be under evolutionary constraints such that the genetic make-up of these genes may be critical for normal development or functioning of the animal and changes within the genes may have detrimental effects. For example, the *GRB2* gene, which had both the lowest slope, i.e. closest to zero (− 0.112) and the lowest R^2^ value (0.036), is highly conserved and under very strong evolutionary constraints in both chickens and in humans [35].
(ii) On the other hand, the genetic diversity of some genes is probably reduced from the founders i.e. selection and fixation of the preferred variants took place prior to domestication, such that there is either no or less possibility for further reductions in genetic diversity. For example, Qanbari et al. [36] reported putative selective sweeps for the genes *EGFR* and *STK17A* across chicken populations, with reduced nucleotide diversity across populations but without significant genetic differentiation between the populations. Previously, a study of these two genes, along with *NDUFA9* and *NTF3,* in different Mexican chickens suggested an influence of natural (adaptive) selection pressure rather than artificial selection [37].
(iii) If an adaptive selection event did not occur prior to domestication, a third explanation is that purifying selection may have continued post-domestication and removed the non-favorable alleles across populations, which led to rapid fixation of the other allele.

## Conclusions

We have analyzed the patterns of genetic diversity (using ${\mathrm{H}}_{\mathrm{o}}$) within a wide range of chicken breeds according to their genetic distances from the chicken wild types. Given the various forces that act on the genome, we conclude that the overall (across the genome) genetic diversity in the chicken can be explained well by the genetic distance to the wild populations. However, evolutionary dynamics have shaped the various functional genomic regions, genes, and pathways in different ways across the breeds, resulting in different patterns of the genetic diversity compared to the overall genome and neutral loci. In particular, non-synonymous sites deviated most from the overall pattern of genetic diversity compared to all other genomic sites. The genes that show rapid changes in genetic diversity may be due to their flexibility to changes according to the populations’ needs, e.g. genes involved in energy metabolism. However, genes that show resistance to change in genetic diversity were associated with critical vital functions e.g. brain development, which is crucial for normal functioning of individuals. Such genes are believed to have maintained low levels of genetic diversity across populations by selection or by evolutionary constraints, and thus the differences or the absence of differences in genomic diversity between breeds (within these genes) do not reflect the genetic distance to the wild type populations for such genes. This study contributes to the knowledge of evolutionary dynamics of different functional genomic regions in the chicken.

## Supplementary Information

**Additional file 1: Table S1. **Categories of chicken breeds.** Table S2. **Relationship between observed heterozygosity and genetic distance to *Gallus gallus* for different groups of populations. **Table S4. **List and functions of the genes in the top and lowest 5% slope ranges.

**Additional file 2: Figure S1.** Observed heterozygosity vs. Reynolds’ genetic distance to the *Gallus gallus* estimated from 1000 SNP samples in 100 replicates. The dashed lines represent the 100 sample sets and the gray area shows the 95% confidence interval. **Figure S2. **Relationship between the observed heterozygosity and genetic differentiation (${F}_{\mathrm{ST}})$ from *G. gallus* (left), and the relationship between ${F}_{\mathrm{ST}}$ and Reynolds’ genetic distance to *G. gallus* (right). The regression lines of the relationships are drawn in red. The R^2^ is equal to 0.885 and 0.988, for the left and right figures, respectively. Different breed categories are denoted in different colors and/or shapes. **Figure S3.** Comparison of the relationship between the genetic distance to *G. g*allus and the observed heterozygosity estimated from the non-synonymous class vs. 100 random samples of the same number of SNPs as the non-synonymous class from the overall SNPs. The black dotted lines represent estimations with the overall SNPs, the red solid line represents the non-synonymous SNPs. The shaded areas represent the 95% confidence intervals of the regression lines. The mean R^2^ of the 100 samples is 0.880 and the mean slope is − 0.708. **Figure S4. **Comparison of the relationship between the genetic distances to *G. gallus* and the observed heterozygosity estimated from intronic SNPs vs. the overall set. The black dashed lines represent estimations with the 100 replicates of 1000 randomly samples SNPs from the intronic SNPs and the red solid line represents overall SNPs. The 95% confidence intervals are shaded in gray. The mean R^2^ and slope of the 100 samples are 0.880 and − 0.711, respectively. **Figure S5. **Comparison of the relationship between the genetic distance to *G. gallus* and the observed heterozygosity estimated from intergenic SNPs vs. the overall set. The black dashed lines represent estimations with the 100 replicates of 1000 randomly sampled SNPs from the intergenic SNPs and the red solid line represents overall SNPs. The 95% confidence intervals are shaded in gray. The mean R^2^ and slope of the 100 samples are 0.878 and − 0.701, respectively.

**Additional file 3: Table S3.** Title: R^2^ and slope values of the relationship between observed heterozygosity and genetic distance to *Gallus gallus *ssp. estimated from the 6303 genes.

**Additional file 4. **Relationships between observed heterozygosity and genetic distance to *G. gallus* for genes in the top 5% slope range.

**Additional file 5. **Relationships between observed heterozygosity and genetic distance to *G. gallus* for genes in the lowest 5% slope range.

## Data Availability

The data used is already published in a previous study [7] and is deposited in the figshare repository and can be accessed through this link: https://doi.org/10.6084/m9.figshare.8003909.
