# Supplementary material for: Genetic diversity in global chicken breeds in relation to their genetic distances to wild populations
Source: Genet Sel Evol. 2021 Apr 14;53:36. doi: 10.1186/s12711-021-00628-z (PMC8048360; doi:10.1186/s12711-021-00628-z)
Supplement: Supplementary file 1 — Additional file 1: Table S1. Categories of chicken breeds. Table S2. Relationship between observed heterozygosity and genetic distance to Gallus gallus for different groups of populations. Table S4. List and functions of the genes in the top and lowest 5% slope ranges. [file 12711_2021_628_MOESM1_ESM.pdf]

Table S1 Categories of chicken breeds

Category	Full name	Number of breeds	Number of individuals
**Wild**	Wild type chicken	2	38
**DE_Europe_Ban**	European bantams sampled in Germany	8	156
**DE_Europe**	European breeds sampled in Germany	35	660
**DE_Asia_Ban**	Asian bantams sampled in Germany	8	177
**DE_Asia**	Asian breeds sampled in Germany	28	531
**Europe_local**	European local breeds sampled across Europe	25	443
**Asia_local**	Asian local breeds sampled across Asia	30	509
**South_America**	South American breeds	4	78
**Africa**	African breeds	22	410
**Overall**		**162**	**3002**

Source: Malomane *et al.* [1]

Table S2 Relationship between observed heterozygosity and genetic distance to *Gallus gallus* for different groups of populations.

	R^2	**Slope**	**SE of slope**
Asia_local	0.343	-0.187	0.049
DE_Asia_all	0.728	-0.543	0.057
Europe_local	0.754	-0.706	0.084
DE_Europe_all	0.898	-0.714	0.038
Africa	0.744	-0.616	0.081

DE_Asia_all and DE_Europe_all consists of all fancy breeds including the bantamised breeds of Asian and European background, respectively. SE – standard error.

Table S4 List and functions of the genes in the top and lowest 5% slope ranges

Gene	Slope	R^2	SNP no	Function
**Top 5% genes**				
SLC25A6	-1.185	0.607	14	Transmembrane transport, mitochondrial inner membrane, integral component of membrane.<br>Calcium signaling pathway.
PLCXD1	-1.098	0.543	10	Lipid metabolic process.
CCM2	-1.095	0.550	10	Vasculogenesis, endothelial cell development, multicellular organism growth, cell-cell junction organization, inner ear development, venous blood vessel morphogenesis, pericardium development, blood vessel endothelial cell differentiation, endothelial tube morphogenesis.
FAM46D	-1.078	0.538	11	Domain of unknown function DUF1693.
SCGN	-1.073	0.547	10	Regulation of cytosolic calcium ion concentration.
MIR33	-1.068	0.577	21	Lipid metabolism and energy homeostasis [2].
MAEL	-1.057	0.444	11	Negative regulation of transcription from RNA polymerase II promoter, cell morphogenesis, synapsis, male meiosis, spermatogenesis, intrinsic apoptotic signaling pathway in response to DNA damage, fertilization, gene silencing by RNA, piRNA metabolic process, DNA methylation involved in gamete generation, negative regulation of apoptotic process, negative regulation of transcription, DNA-templated, regulation of organ growth, sequence-specific DNA binding.
IPCEF1	-1.042	0.459	10	Pleckstrin homology domain, Pleckstrin homology-like domain.
PTGFRN	-1.038	0.517	14	Immunoglobulin subtype, immunoglobulin-like domain, immunoglobulin V-set, immunoglobulin-like fold, lipid particle organization.
GTF3C1	-1.038	0.536	17	Transcription factor TFIIIC complex, nucleolus, membrane, intracellular ribonucleoprotein complex.
SLMO1	-1.030	0.575	29	Positive regulation of protein targeting to mitochondrion.
P2RY8	-1.026	0.607	22	G-protein coupled receptor activity, thrombin receptor activity, G-protein coupled purinergic nucleotide receptor activity, blood coagulation, positive regulation of Rho protein signal transduction, G-protein coupled purinergic nucleotide receptor signaling pathway, positive regulation of cytosolic calcium ion concentration involved in phospholipase C-activating G-protein coupled signaling pathway, thrombin receptor signaling pathway.
TRAF3IP2	-1.023	0.496	14	B cell apoptotic process, positive regulation of defense response to virus by host, humoral immune response, positive regulation of I-kappaB kinase/NF-kappaB signaling, immunoglobulin secretion.
ACP6	-1.022	0.554	18	Hematopoietic progenitor cell differentiation, acid phosphatase activity.
GJA5	-1.017	0.478	14	Skeletal system development, angiogenesis, renal system process involved in regulation of systemic arterial blood pressure, outflow tract morphogenesis, cardiac conduction system development, mitral valve development, pulmonary valve formation, septum primum development,

				atrial ventricular junction remodeling, cell communication, gap junction assembly, embryonic limb morphogenesis, embryonic heart tube development, foramen ovale closure, regulation of vasodilation, artery morphogenesis, regulation of ventricular cardiac muscle cell membrane repolarization, regulation of atrial cardiac muscle cell membrane depolarization, regulation of ventricular cardiac muscle cell membrane depolarization, ventricular septum morphogenesis, atrial septum morphogenesis, ventricular cardiac muscle cell action potential, SA node cell action potential, bundle of His cell to Purkinje myocyte communication by electrical coupling, regulation of heart rate by cardiac conduction, regulation of AV node cell action potential, regulation of bundle of His cell action potential, regulation of Purkinje myocyte action potential, regulation of atrial cardiac muscle cell action potential, regulation of renin secretion into blood stream, regulation of membrane depolarization during cardiac muscle cell action potential, gap junction hemi-channel activity, gap junction channel activity involved in AV node cell-bundle of His cell electrical coupling.
C1GALT1	-1.015	0.515	17	Angiogenesis, kidney development, O-glycan processing, core 1, intestinal epithelial cell development.
HCLS1	-1.013	0.531	18	Tight junction, negative regulation of transcription from RNA polymerase II promoter, positive regulation of cell proliferation, response to hormone, positive regulation of phosphatidylinositol 3-kinase signaling, actin filament polymerization, erythrocyte differentiation, regulation of actin filament polymerization, positive regulation of granulocyte differentiation, positive regulation of peptidyl-serine phosphorylation, positive regulation of tyrosine phosphorylation of STAT protein, positive regulation of transcription factor import into nucleus, positive regulation of macrophage differentiation, positive regulation of transcription from RNA polymerase II promoter, positive regulation of protein kinase B signaling, cellular response to cytokine stimulus, negative regulation of leukocyte apoptotic process.
NHSL1	-1.011	0.534	10	Not well known.
XRCC6	-1.010	0.589	24	Non-homologous end-joining, telomere maintenance, double-strand break repair via nonhomologous end joining, DNA recombination, transcription, DNA-templated, DNA duplex unwinding, negative regulation of transcription, DNA-templated, positive regulation of transcription from RNA polymerase II promoter, regulation of smooth muscle cell proliferation, protein heterotetramerization, cellular response to gamma radiation, cellular response to X-ray, double-strand break repair via classical nonhomologous end joining, damaged DNA binding, double-stranded DNA binding, double-stranded telomeric DNA binding, ATP-dependent DNA helicase activity, ATP binding, telomeric DNA binding, transcription regulatory region DNA binding, poly(A) RNA binding, macromolecular complex binding, 5'-

				deoxyribose-5-phosphate lyase activity.
SLC22A15	-1.009	0.508	12	Substrate-specific transmembrane transporter activity, integral component of membrane.
UCHL5	-1.005	0.591	17	Ubiquitin-dependent protein catabolic process, protein deubiquitination, Ino80 complex.
LOC100858381	-1.004	0.446	10	Not well known.
LSM6	-1.003	0.544	11	mRNA splicing, via spliceosome, maturation of SSU-rRNA.<br>RNA degradation and spliceosome pathways.
TERF1	-1.002	0.632	44	Double-stranded telomeric DNA binding, telomeric DNA binding, telomere maintenance, telomeric loop formation, meiotic telomere clustering.
MIR1755	-1.001	0.492	10	Not well known.
PPPDE1	-0.999	0.536	11	Not well known.
PDSS2	-0.998	0.510	25	Protein deubiquitination.
MON2	-0.996	0.507	11	Vesicle-mediated transport.
WWC2	-0.995	0.601	17	Negative regulation of transcription from RNA polymerase II promoter, negative regulation of hippo signaling, negative regulation of organ growth.
RPL7	-0.994	0.543	16	Ribosome, maturation of LSU-rRNA from tricistronic rRNA transcript (SSU-rRNA, 5.8S rRNA, LSU-rRNA), cytoplasmic translation, rRNA processing, ribosomal large subunit biogenesis, DNA binding, mRNA binding, structural constituent of ribosome, protein homodimerization activity, poly(A) RNA binding.
TBC1D2B	-0.990	0.492	16	GTPase activator activity, Rab GTPase binding, intracellular protein transport, regulation of vesicle fusion, activation of GTPase activity.
LOC420419	-0.989	0.562	28	MAPK signaling pathway, GnRH signaling pathway.
**Lowest 5% genes**				
MTA3	-0.342	0.122	19	Chromatin binding, sequence-specific DNA binding.
PTPRS	-0.336	0.333	44	Spinal cord development, cerebellum development, hippocampus development, cerebral cortex development, corpus callosum development, extracellular matrix organization, establishment of endothelial intestinal barrier.
CKMT1A	-0.332	0.282	15	Phosphorylation, arginine and proline metabolism, metabolic pathways.
RAP1GAP2	-0.332	0.215	16	GTPase activator activity, regulation of cell size, negative regulation of neuron projection development, positive regulation of GTPase activity, regulation of small GTPase mediated signal transduction.
RPS6KA1	-0.332	0.155	24	Magnesium ion binding, protein serine/threonine kinase activity, protein serine/threonine/tyrosine kinase activity, ATP binding, cysteine-type endopeptidase inhibitor activity involved in apoptotic process, protein phosphorylation, apoptotic process, intracellular signal transduction, negative regulation of cysteine-type endopeptidase activity involved in apoptotic process, positive regulation of transcription from RNA polymerase II promoter, positive regulation of hepatic stellate cell

				activation.
KIF1B	-0.325	0.132	16	Microtubule-based movement, neuron-neuron synaptic transmission, neuromuscular synaptic transmission, anterograde axonal transport, cytoskeleton-dependent intracellular transport, mitochondrion transport along microtubule.
STXBP6	-0.324	0.242	25	Exocytosis, Golgi to plasma membrane transport, regulation of SNARE complex assembly, exocyst localization.
PRMT7	-0.319	0.231	18	Spliceosomal snRNP assembly, regulation of gene expression, DNA-templated, regulation of transcription, cell differentiation, DNA methylation involved in gamete generation, histone H4-R3 methylation.
TMEM39B	-0.314	0.163	10	Not well known
C20H20ORF4	-0.313	0.183	15	Not well known.
FGFR1	-0.311	0.268	28	Negative regulation of transcription from RNA polymerase II promoter, angiogenesis, ureteric bud development, organ induction, positive regulation of mesenchymal cell proliferation, chondrocyte differentiation, epicardial cell to mesenchymal cell transition, vacuolar phosphate transport, sensory perception of sound, positive regulation of cell proliferation, fibroblast growth factor receptor signaling pathway, positive regulation of phospholipase C activity, positive regulation of neuron projection development, peptidyl-tyrosine phosphorylation, ventricular zone neuroblast division, embryonic limb morphogenesis, midbrain development, fibroblast growth factor receptor signaling pathway involved in orbitofrontal cortex development, inner ear morphogenesis, outer ear morphogenesis, middle ear morphogenesis, positive regulation of MAP kinase activity, positive regulation of cell cycle, positive regulation of transcription, DNA-templated, protein autophosphorylation, paraxial mesoderm development, regulation of lateral mesodermal cell fate specification, cell maturation, mesenchymal cell differentiation, positive regulation of cardiac muscle cell proliferation, auditory receptor cell development, branching involved in salivary gland morphogenesis, lung-associated mesenchyme development, regulation of branching involved in salivary gland morphogenesis by mesenchymal-epithelial signaling, vitamin D3 metabolic process, positive regulation of MAPKKK cascade by fibroblast growth factor receptor signaling pathway, negative regulation of fibroblast growth factor production, positive regulation of endothelial cell chemotaxis to fibroblast growth factor, positive regulation of parathyroid hormone secretion, regulation of extrinsic apoptotic signaling pathway in absence of ligand.<br>MAPK signaling pathway, adherens junction, and regulation of actin cytoskeleton pathways.
PAFAH1B1	-0.307	0.299	12	Establishment of mitotic spindle orientation, ameboidal-type cell migration, acrosome assembly, neuron migration, positive regulation of cytokine-mediated signaling pathway, mitotic nuclear division, nuclear migration,

				chemical synaptic transmission, germ cell development, neuroblast proliferation, learning or memory, retrograde axonal transport, adult locomotory behavior, protein secretion, transmission of nerve impulse, corpus callosum morphogenesis, hippocampus development, layer formation in cerebral cortex, neurogenesis, actin cytoskeleton organization, microtubule organizing center organization, osteoclast development, positive regulation of embryonic development, establishment of planar polarity of embryonic epithelium, regulation of GTPase activity, cortical microtubule organization, negative regulation of JNK cascade, vesicle transport along microtubule, brain morphogenesis, neuromuscular process controlling balance, nuclear envelope disassembly, cell division, maintenance of centrosome location, auditory receptor cell development, positive regulation of dendritic spine morphogenesis, regulation of microtubule cytoskeleton organization, cochlea development, microtubule cytoskeleton organization involved in establishment of planar polarity, regulation of microtubule motor activity.
RAB8A	-0.307	0.311	33	Vesicle docking involved in exocytosis, small GTPase mediated signal transduction, axonogenesis, protein secretion, cilium assembly, Golgi vesicle fusion to target membrane.<br>Endocytosis pathway.
NEFM	-0.303	0.340	33	Axon development, mitophagy in response to mitochondrial depolarization.
MYL9	-0.298	0.126	12	Signal transduction mechanisms, cytoskeleton, cell division and chromosome partitioning, calcium ion binding, myosin heavy chain binding, platelet aggregation.
EGFR	-0.297	0.135	14	Cell, digestive tract and salivary gland morphogenesis, transmembrane receptor protein tyrosine kinase and epidermal growth factor receptor signaling pathways, learning or memory, cell proliferation, epidermis development, single organismal cell-cell adhesion, cerebral cortex cell migration, positive regulation of cell growth, positive regulation of cell migration, positive regulation of cyclin-dependent protein serine/threonine kinase activity involved in G1/S transition of mitotic cell cycle, positive regulation of catenin import into nucleus, negative regulation of protein catabolic process, negative regulation of apoptotic process, positive regulation of MAP kinase activity, positive regulation of nitric oxide biosynthetic process, positive regulation of DNA repair and replication, positive regulation of transcription, protein autophosphorylation, positive regulation of fibroblastand epithelial cell proliferation, regulation of peptidyl-tyrosine phosphorylation, regulation of nitric-oxide synthase activity, positive regulation of protein kinase B signaling, eyelid development in camera-type eye, response to UV-A, positive regulation of ERK1 and ERK2 cascade, cellular response to amino acid stimulus, epidermal growth factor stimulus and estradiol stimulus, positive regulation of production of miRNAs involved in gene silencing by miRNA. MAPK signaling pathway,

				ErbB signaling pathway, Calcium signaling pathway, FoxO signaling pathway, Endocytosis, Dorso-ventral axis formation, Focal adhesion, Adherens junction, Gap junction, Regulation of actin cytoskeleton, GnRH signaling pathway.
BRIP1	-0.280	0.261	18	DNA repair, regulation of transcription from RNA polymerase II promoter, DNA duplex unwinding.
SPTBN1	-0.279	0.168	15	Mitotic cytokinesis, common-partner SMAD protein phosphorylation, SMAD protein import into nucleus, Golgi to plasma membrane protein transport, membrane assembly, protein targeting to plasma membrane, positive regulation of interleukin-2 secretion, positive regulation of protein localization to plasma membrane.
DOCK5	-0.276	0.192	12	Small GTPase mediated signal transduction, negative regulation of vascular smooth muscle contraction, positive regulation of vascular associated smooth muscle cell migration.
NDUFA9	-0.271	0.131	14	Cell envelope biogenesis, outer membrane, carbohydrate transport and metabolism. Oxidative phosphorylation, Metabolic pathways.
ADAM28	-0.267	0.200	13	Integral component of membrane, metalloendopeptidase activity, epidermal growth factor-like domain, peptidase M12B, ADAM/reprolysin, blood coagulation inhibitor EGF-like conserved site, disintegrin conserved site.
KCTD9	-0.265	0.239	11	Protein homooligomerization.
RTN4	-0.260	0.129	13	Axonogenesis, axonal fasciculation, endoplasmic reticulum membrane, endomembrane system, integral component of membrane.
LOC415713	-0.114	0.143	11	Not well known.
GNRH1	-0.252	0.159	14	Reproduction, multicellular organism development, regulation of gene expression, positive regulation of luteinizing hormone secretion, response to alkaloid, response to ethanol, regulation of hormone biosynthetic process, response to steroid hormone, response to serotonin, regulation of ovarian follicle development, regulation of testosterone secretion, negative regulation of neuron migration. GnRH signaling pathway.
EBF2	-0.251	0.305	31	Cell fate determination, transcription, DNA-templated, regulation of transcription, DNA-templated, multicellular organism development, positive regulation of chromatin binding, positive regulation of transcription from RNA polymerase II promoter, brown fat cell differentiation, adipose tissue development.
ESRP2	-0.235	0.118	15	mRNA processing, regulation of RNA splicing, positive regulation of epithelial cell proliferation, epithelial tube branching involved in lung morphogenesis, branching involved in salivary gland morphogenesis.
WHSC1L1	-0.232	0.124	11	Chromatin structure and dynamics, zinc ion binding, histone-lysine N-methyltransferase activity. Lysine degradation.
STK17A	-0.232	0.089	13	Intracellular signal transduction, positive regulation of fibroblast apoptotic process, regulation of reactive oxygen species metabolic process.
NTF3	-0.174	0.056	14	Activation of MAPK activity, positive regulation of receptor internalization, transmembrane receptor protein

				tyrosine kinase signaling pathway, cell-cell signaling, positive regulation of cell proliferation, positive regulation of cell migration, activation of protein kinase B activity, positive regulation of peptidyl-serine phosphorylation, regulation of apoptotic process, negative regulation of neuron apoptotic process, regulation of neuron differentiation and morphogenesis, regulation of peptidyl-tyrosine phosphorylation, induction of positive chemotaxis, activation of GTPase activity, positive regulation of actin cytoskeleton reorganization. MAPK pathway.
DPYSL2	-0.141	0.057	10	Endocytosis, cytoskeleton organization, axon guidance, brain development, regulation of axon extension.
GRB2	-0.112	0.036	16	Insulin receptor signaling pathway, positive regulation of signal transduction, cell differentiation, positive regulation of actin filament polymerization, receptor internalization, signal transduction in response to DNA damage, regulation of MAPK cascade, anatomical structure formation involved in morphogenesis, cellular response to ionizing radiation, positive regulation of reactive oxygen species metabolic process. MAPK signaling pathway, ErbB signaling pathway, FoxO signaling pathway, Dorso-ventral axis formation, Focal adhesion, Gap junction, Jak-STAT signaling pathway, Insulin signaling pathway, GnRH signaling pathway.

1. Malomane DK, Simianer H, Weigend A, Reimer C, Schmitt AO, Weigend S. The SYNBREED chicken diversity panel : A global resource to assess chicken diversity at high genomic resolution. BMC Genomics; 2019;20:345.

2. Shao F, Wang X, Yu J, Jiang H, Zhu B, Gu Z. Expression of miR-33 from an SREBF2 intron targets the FTO gene in the chicken. PLoS One. 2014;9.
