# Supplementary material for: Genetic diversity in global chicken breeds in relation to their genetic distances to wild populations
Source: Genet Sel Evol. 2021 Apr 14;53:36. doi: 10.1186/s12711-021-00628-z (PMC8048360; doi:10.1186/s12711-021-00628-z)
Supplement: Supplementary file 2 — Additional file 2: Figure S1. Observed heterozygosity vs. Reynolds’ genetic distance to the Gallus gallus estimated from 1000 SNP samples in 100 replicates. The dashed lines represent the 100 sample sets and the gray area shows the 95% confidence interval. Figure S2. Relationship between the observed heterozygosity and genetic differentiation (${F}_{\mathrm{ST}})$FST) from G. gallus (left), and the relationship between ${F}_{\mathrm{ST}}$FST and Reynolds’ genetic distance to G. gallus (right). The regression lines of the relationships are drawn in red. The R2 is equal to 0.885 and 0.988, for the left and right figures, respectively. Different breed categories are denoted in different colors and/or shapes. Figure S3. Comparison of the relationship between the genetic distance to G. gallus and the observed heterozygosity estimated from the non-synonymous class vs. 100 random samples of the same number of SNPs as the non-synonymous class from the overall SNPs. The black dotted lines represent estimations with the overall SNPs, the red solid line represents the non-synonymous SNPs. The shaded areas represent the 95% confidence intervals of the regression lines. The mean R2 of the 100 samples is 0.880 and the mean slope is − 0.708. Figure S4. Comparison of the relationship between the genetic distances to G. gallus and the observed heterozygosity estimated from intronic SNPs vs. the overall set. The black dashed lines represent estimations with the 100 replicates of 1000 randomly samples SNPs [file 12711_2021_628_MOESM2_ESM.pdf]

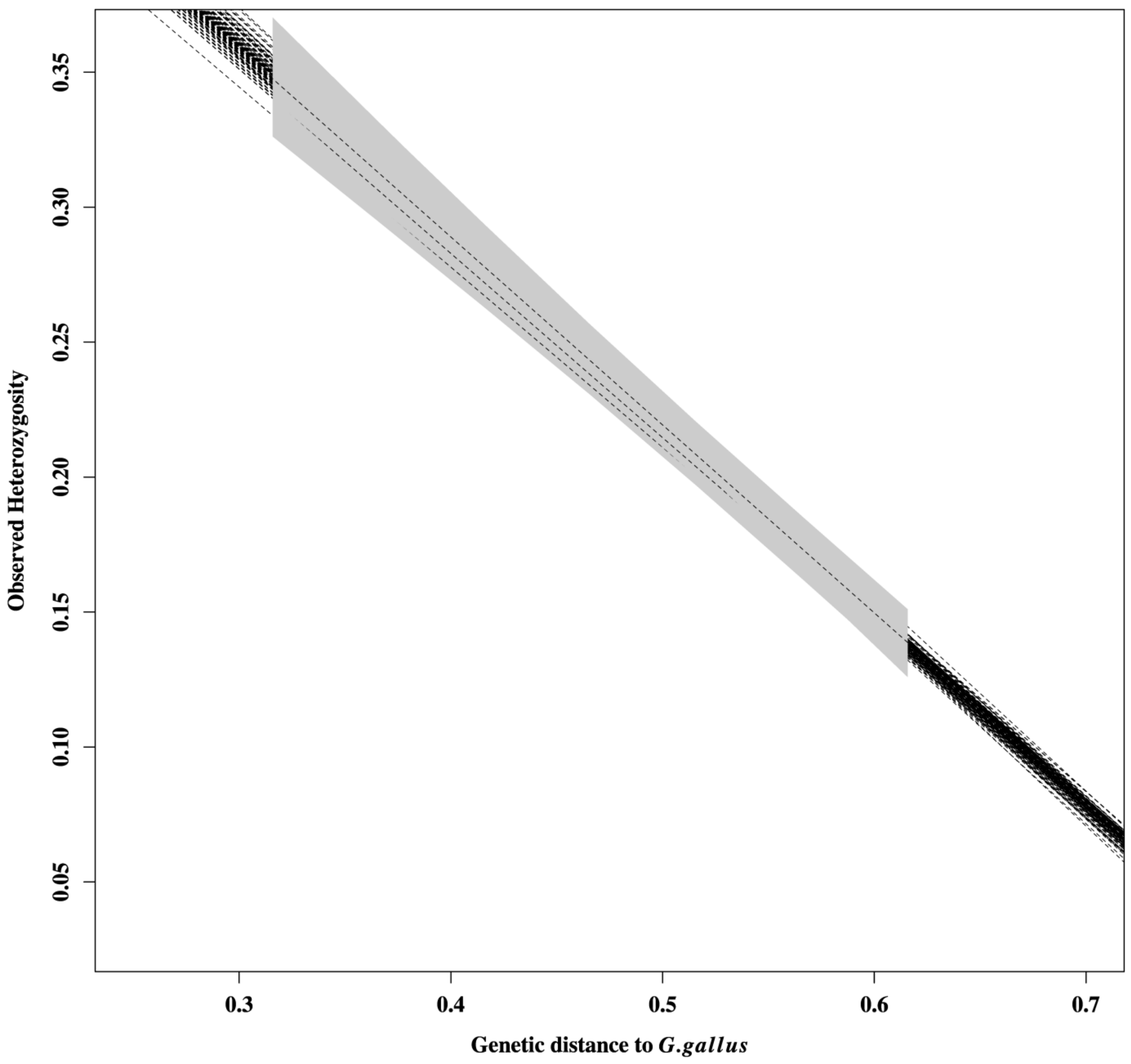

Figure S1 Observed heterozygosity vs. Reynolds' genetic distance to the *Gallus gallus* estimated from 1000 SNP samples in 100 replicates. The dashed lines represent the 100 sample sets and the gray area shows a 95% confidence interval.

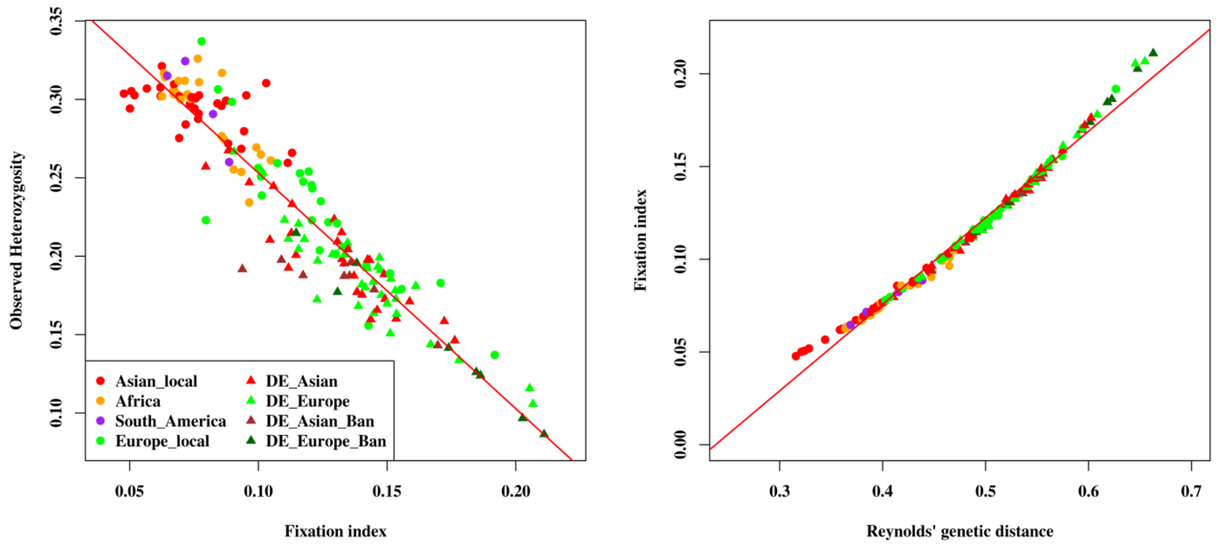

Figure S2 Title: Relationship between the observed heterozygosity and genetic differentiation (*F_{ST}*) from *G. gallus* (left), and the relationship between *F_{ST}* and Reynolds' genetic distance to *G. gallus* (right). The regression lines of the relationships are drawn in red. The R^2 is equal to 0.885 and 0.988, for the left and right figures, respectively. Different breed categories are denoted in different colors and/or shapes.

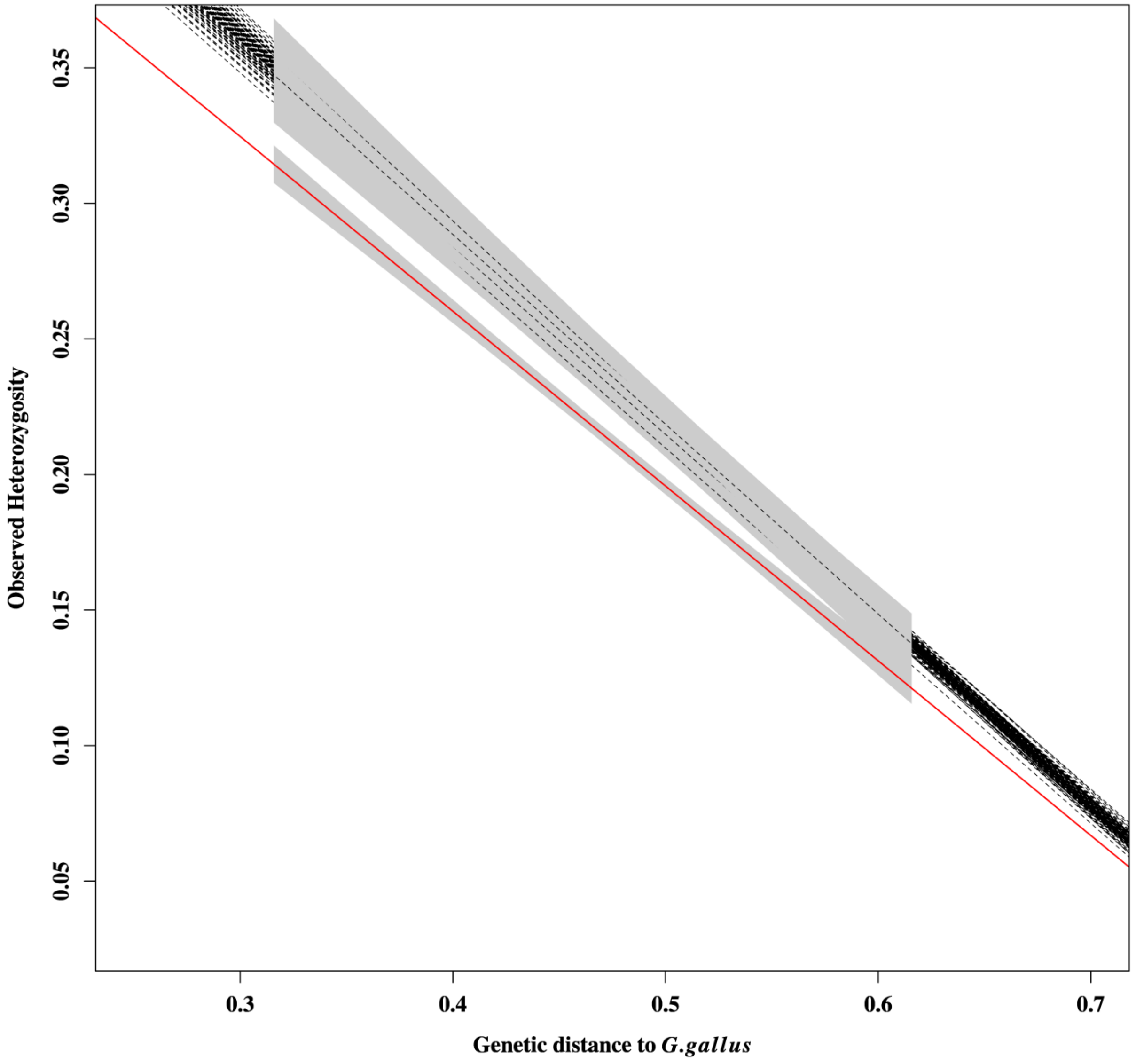

Figure S3 Comparison of the relationship between the genetic distance to *G. g*allus and the observed heterozygosity estimated from the non-synonymous class vs. 100 random samples of the same number of SNPs as the non-synonymous class from the overall SNPs. The black dotted lines represent estimations with the overall SNPs, the red solid line represents the non-synonymous SNPs. The shaded areas represent the 95% confidence intervals of the regression lines. The mean R^2 of the 100 samples is 0.880 and the mean slope is -0.708.

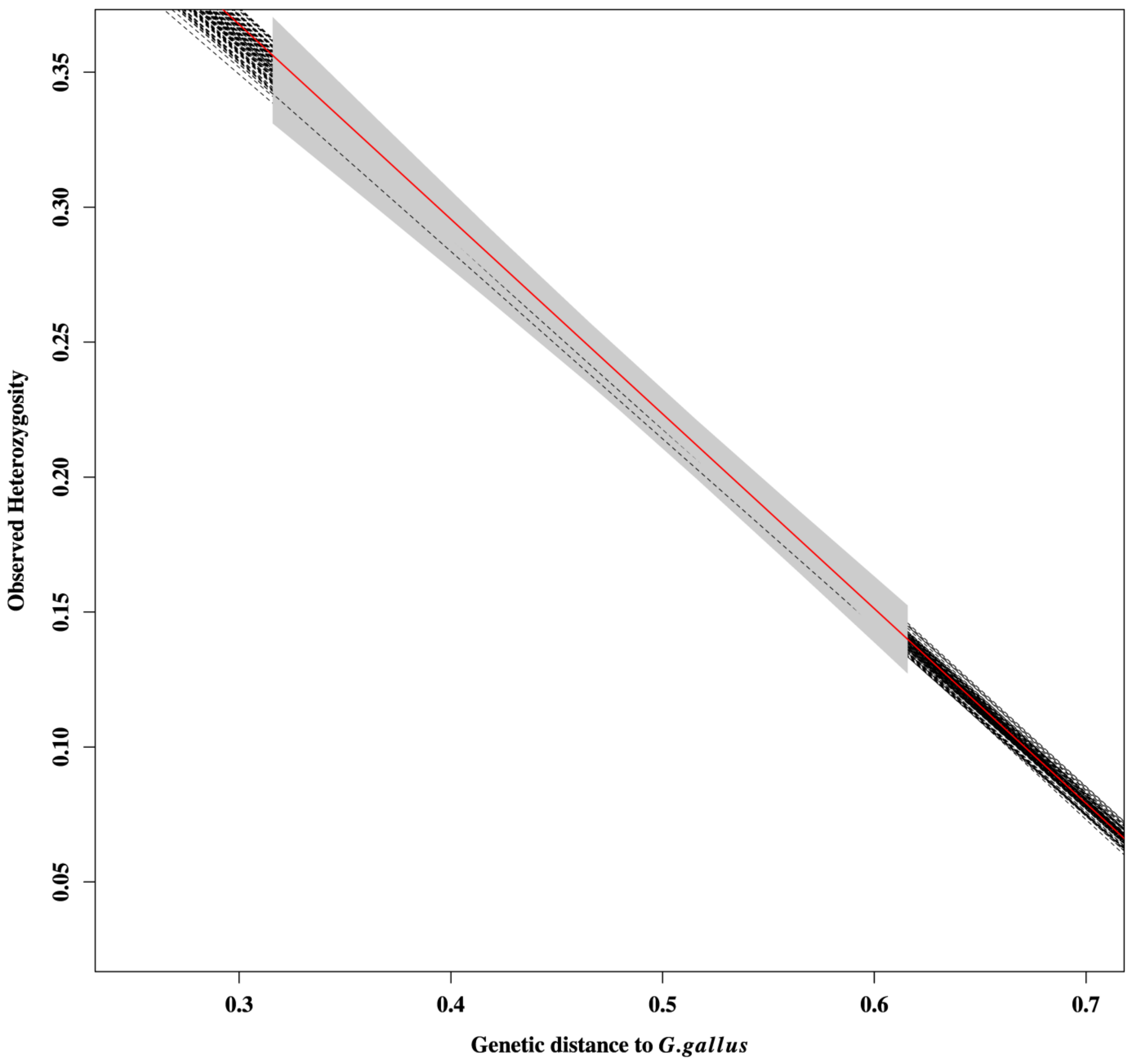

Figure S4 Comparison of the relationship between the genetic distances to *G. gallus* and the observed heterozygosity estimated from intronic SNPs vs. the overall set. The black dashed lines represent estimations with the 100 replicates of 1000 randomly samples SNPs from the intronic SNPs and the red solid line represents overall SNPs. The 95% confidence intervals are shaded in gray. The mean R^2 and slope of the 100 samples are 0.880 and -0.711, respectively.

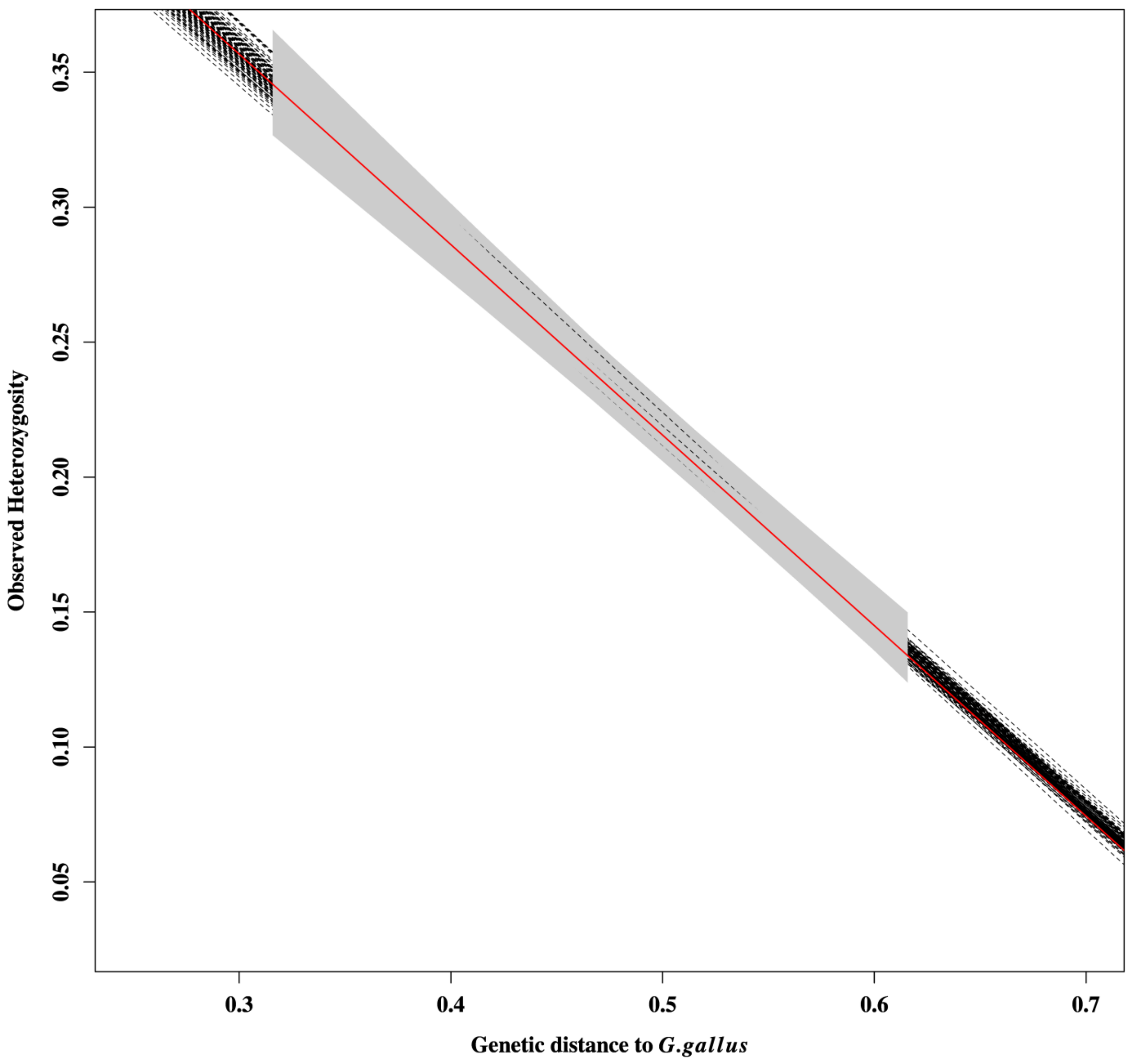

Figure S5 Comparison of the relationship between the genetic distance to *G. gallus* and the observed heterozygosity estimated from intergenic SNPs vs. the overall set. The black dashed lines represent estimations with the 100 replicates of 1000 randomly sampled SNPs from the intergenic SNPs and the red solid line represents overall SNPs. The 95% confidence intervals are shaded in gray. The mean R^2 and slope of the 100 samples are 0.878 and -0.701, respectively.
