# Supplementary figures and images for: Genetic diversity in global chicken breeds in relation to their genetic distances to wild populations
Source: Genet Sel Evol. 2021 Apr 14;53:36. doi: 10.1186/s12711-021-00628-z (PMC8048360; doi:10.1186/s12711-021-00628-z)

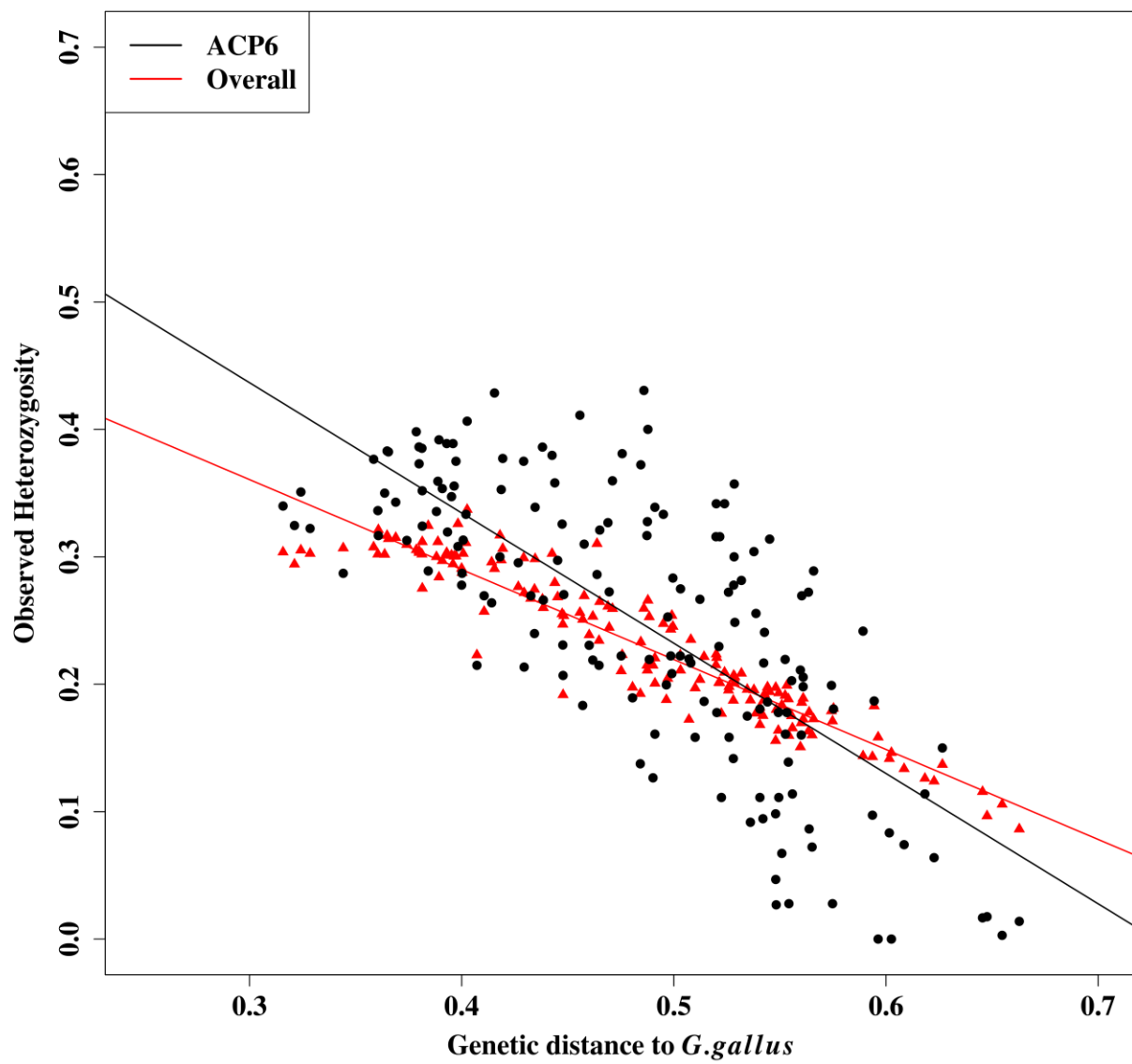

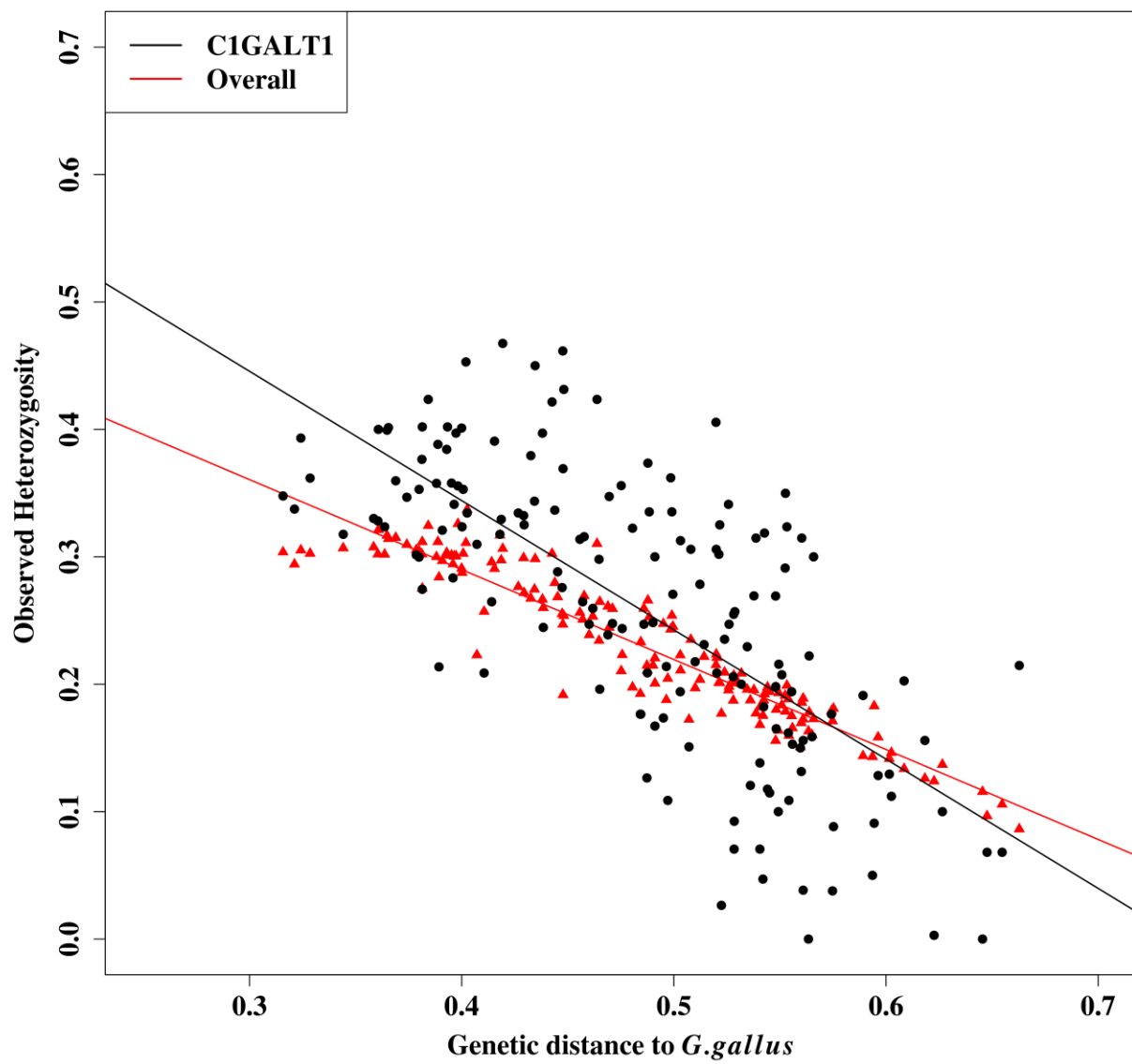

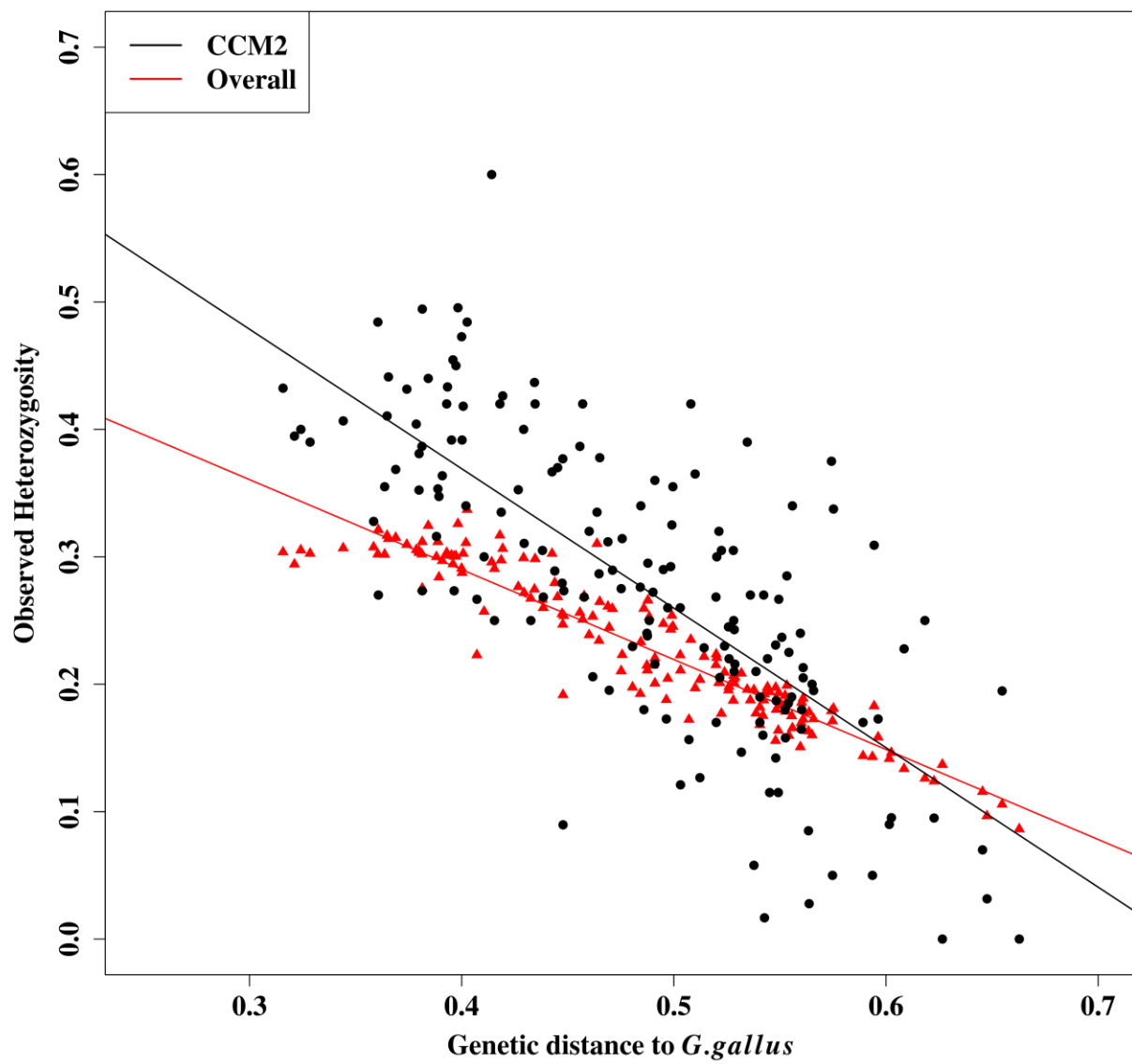

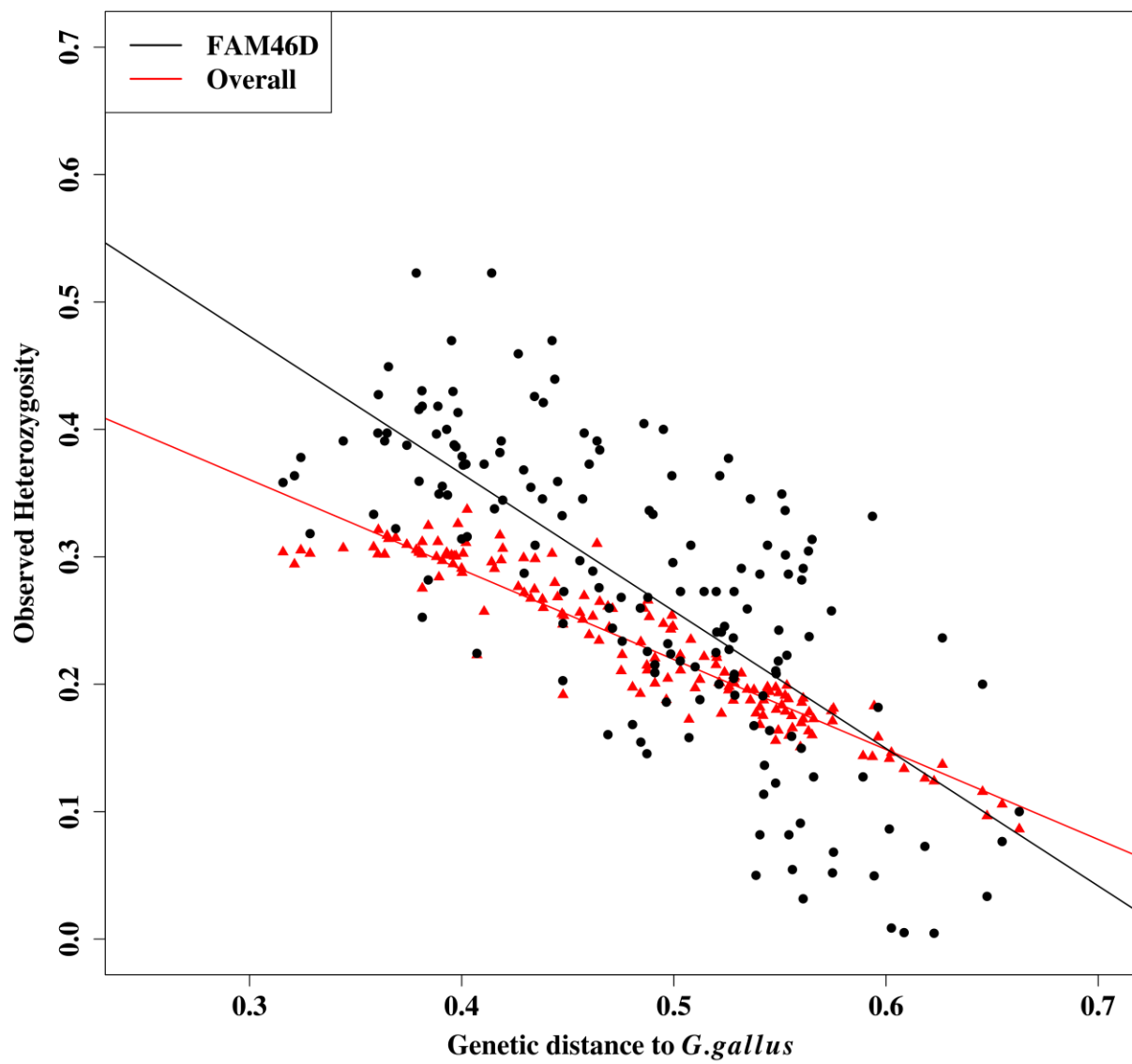

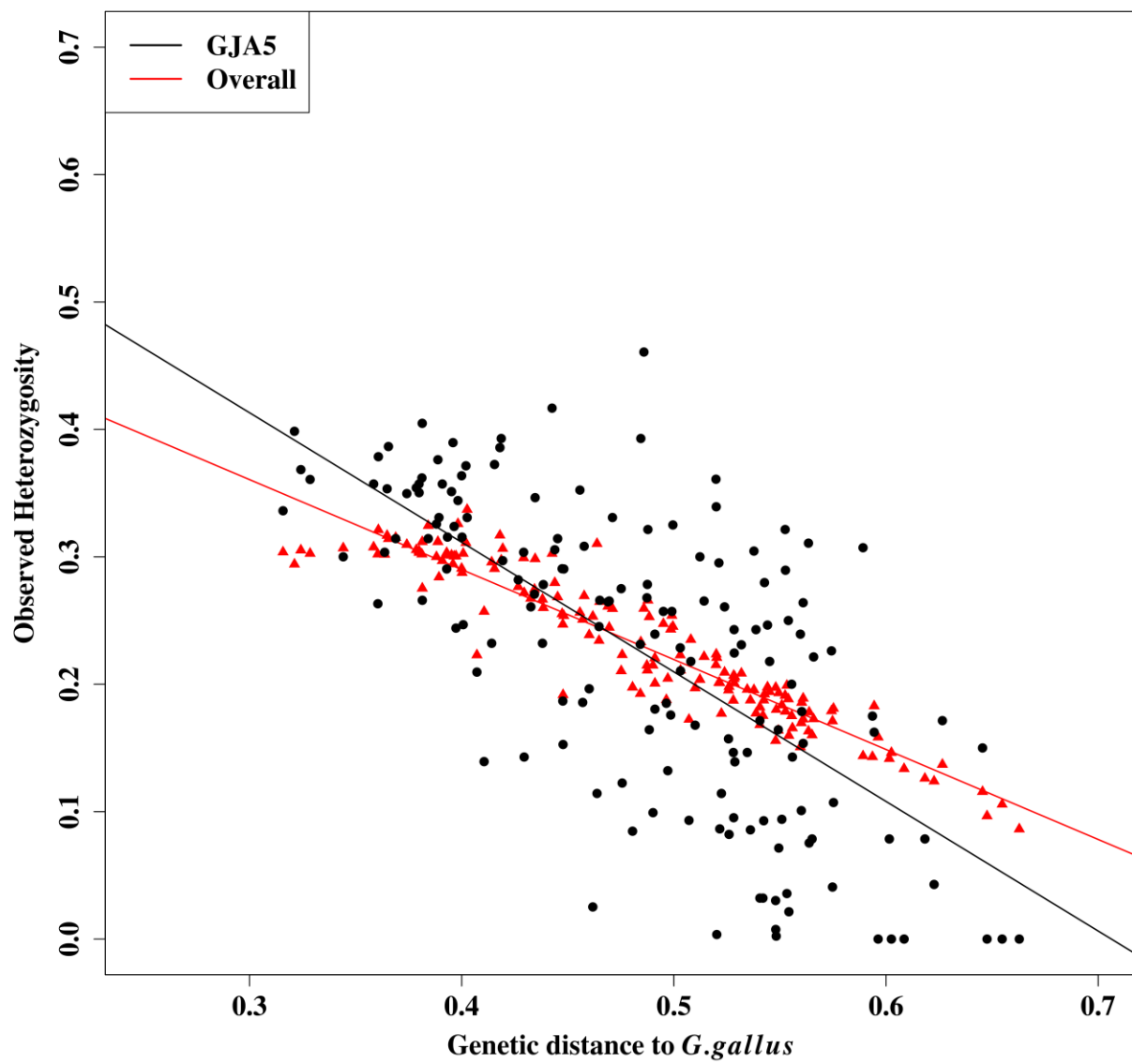

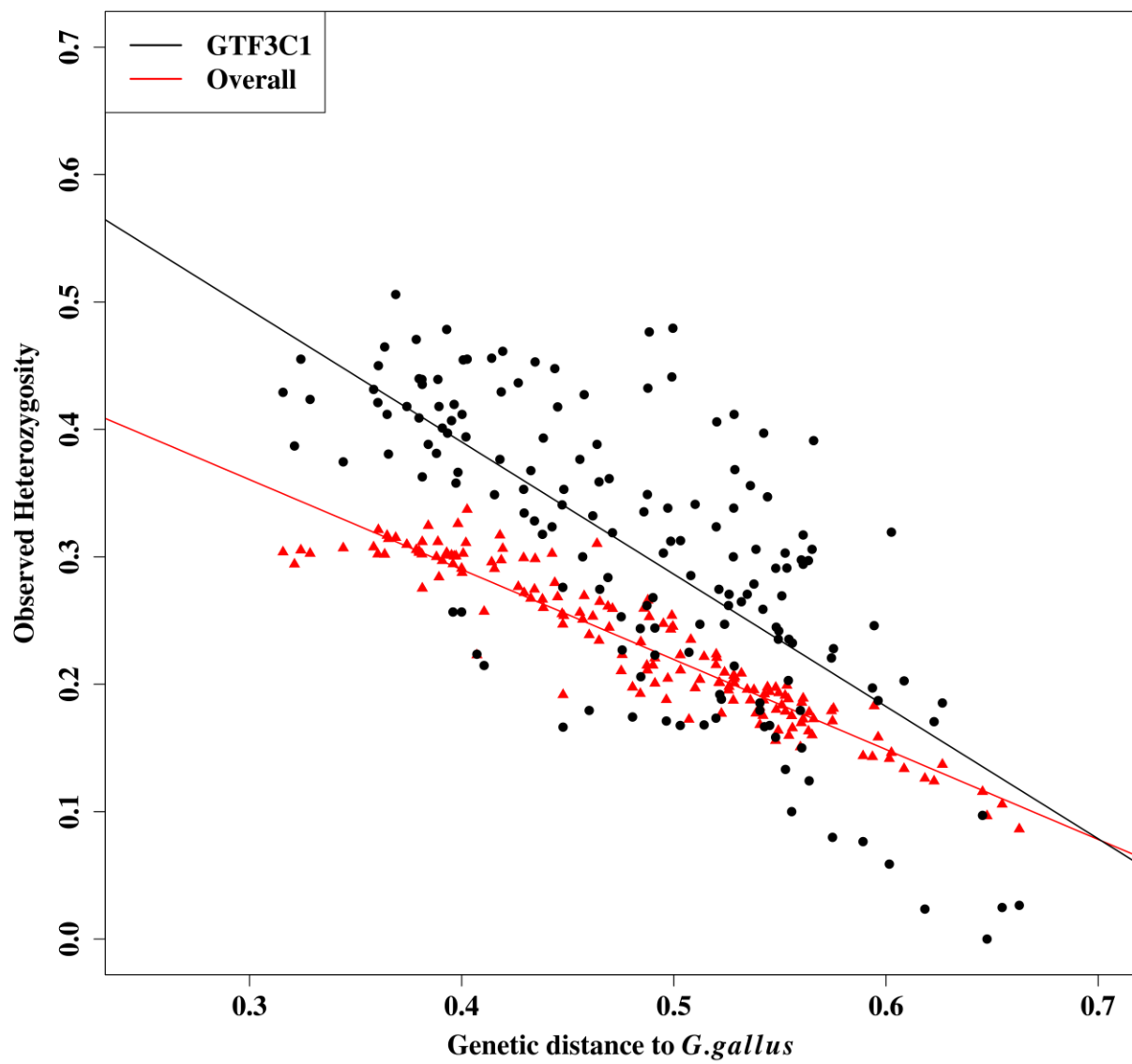

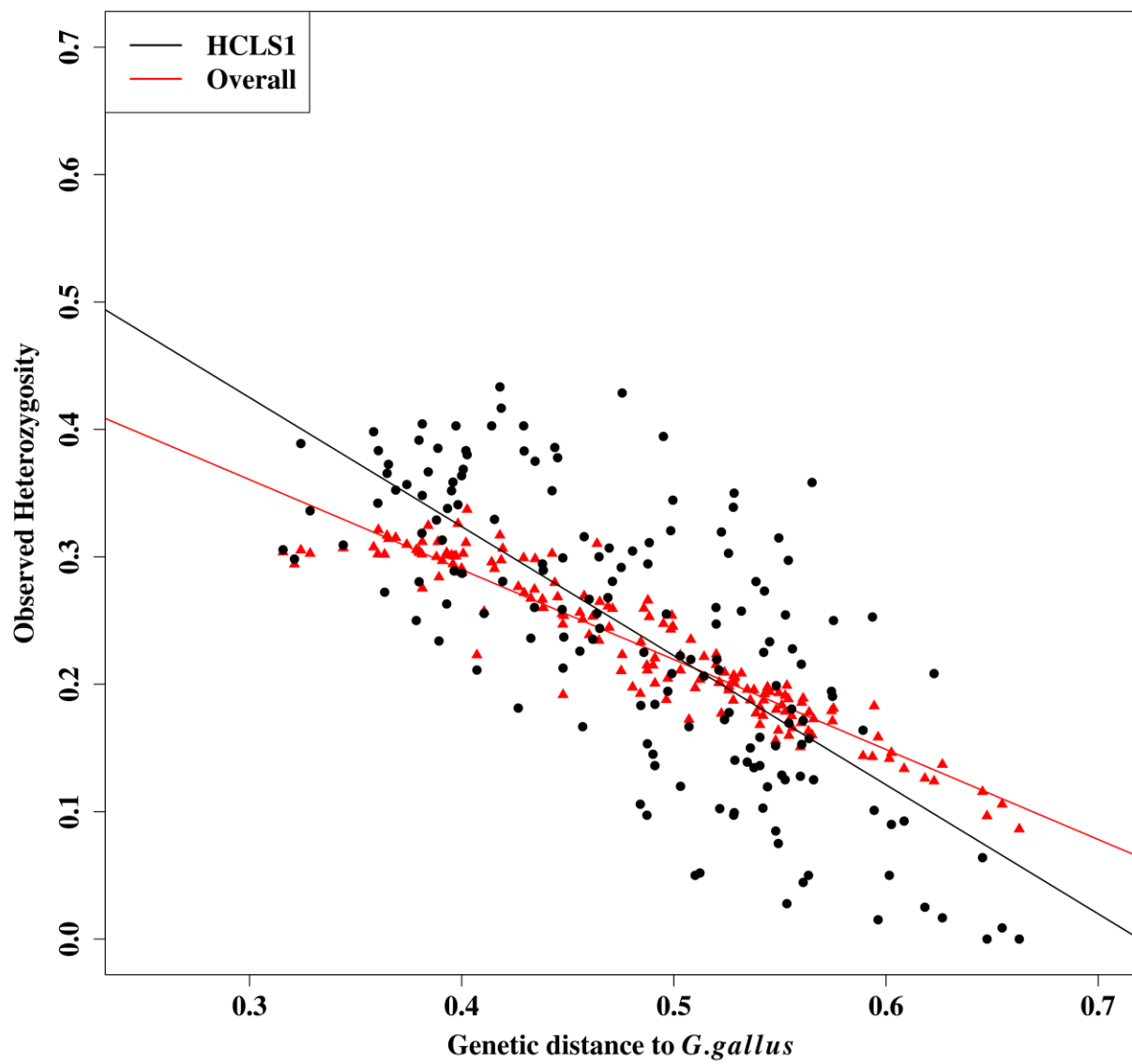

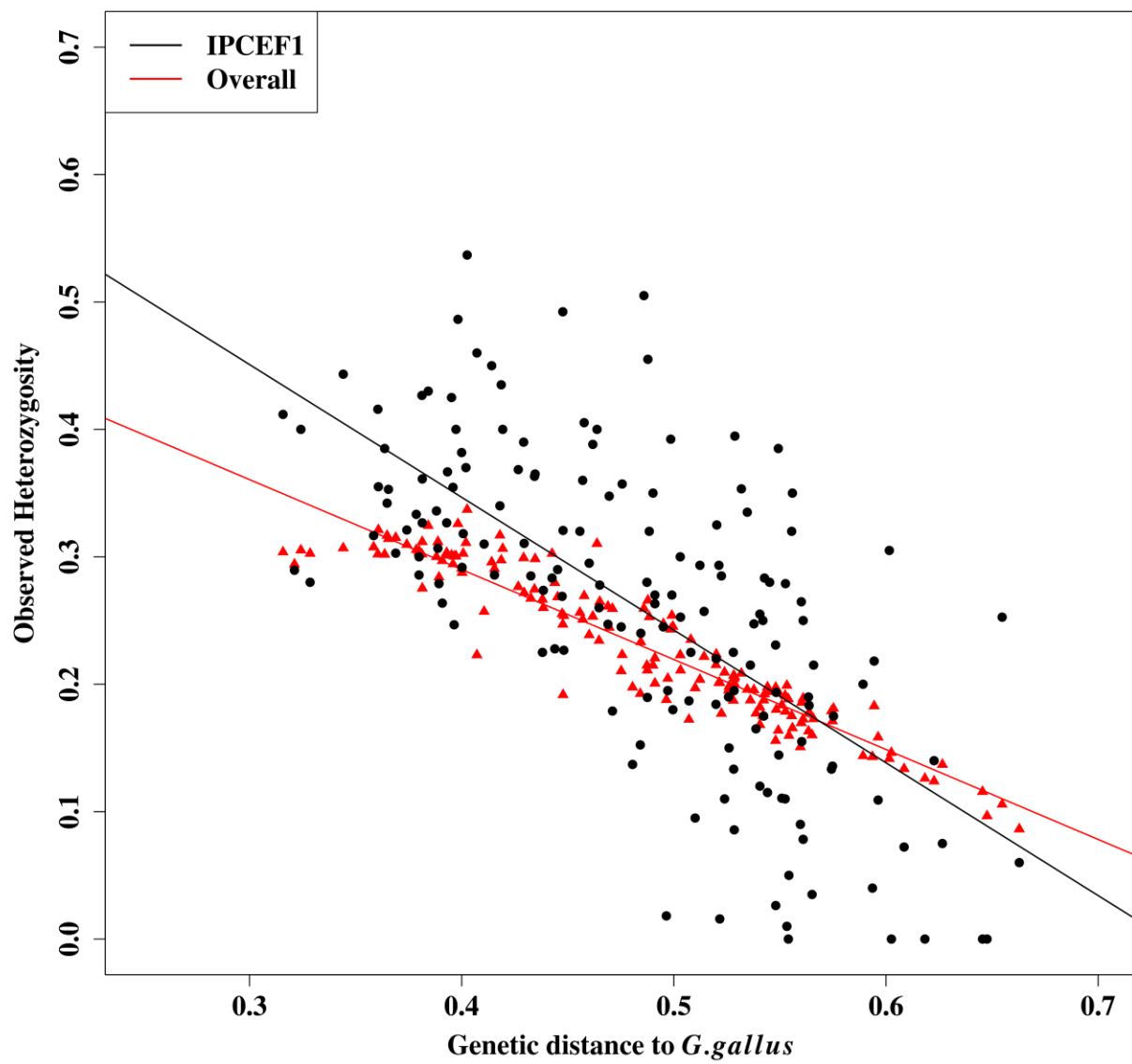

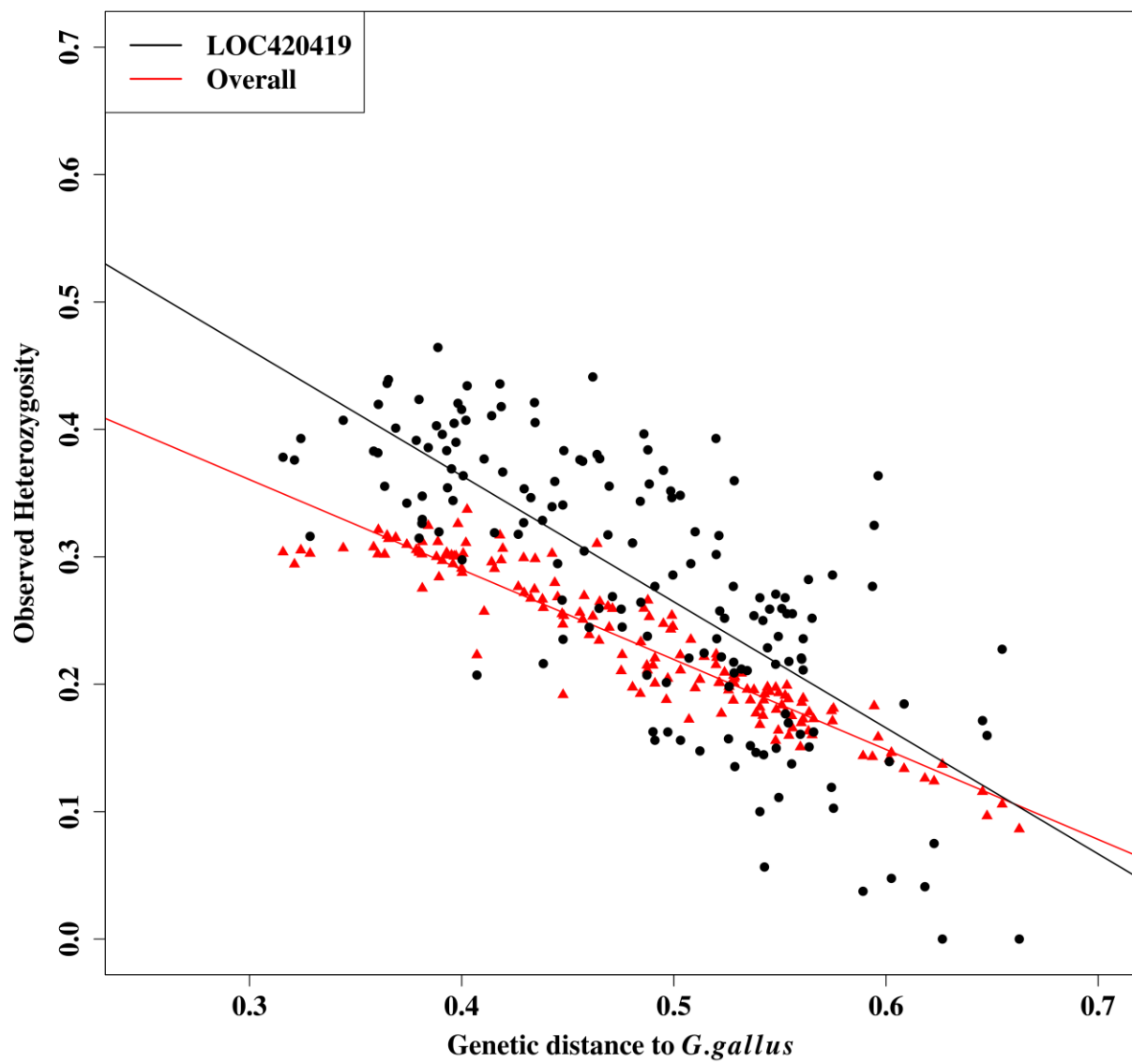

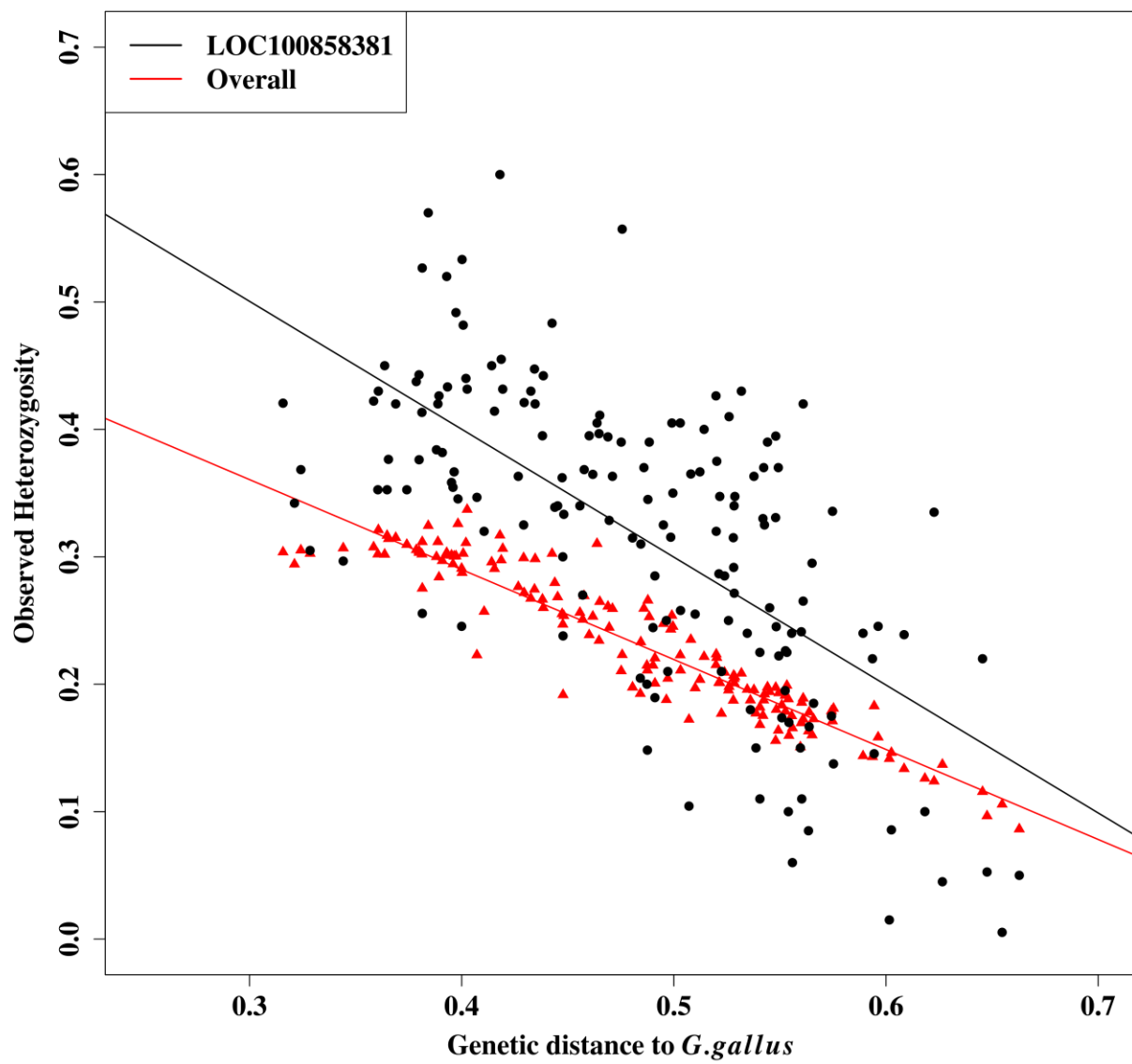

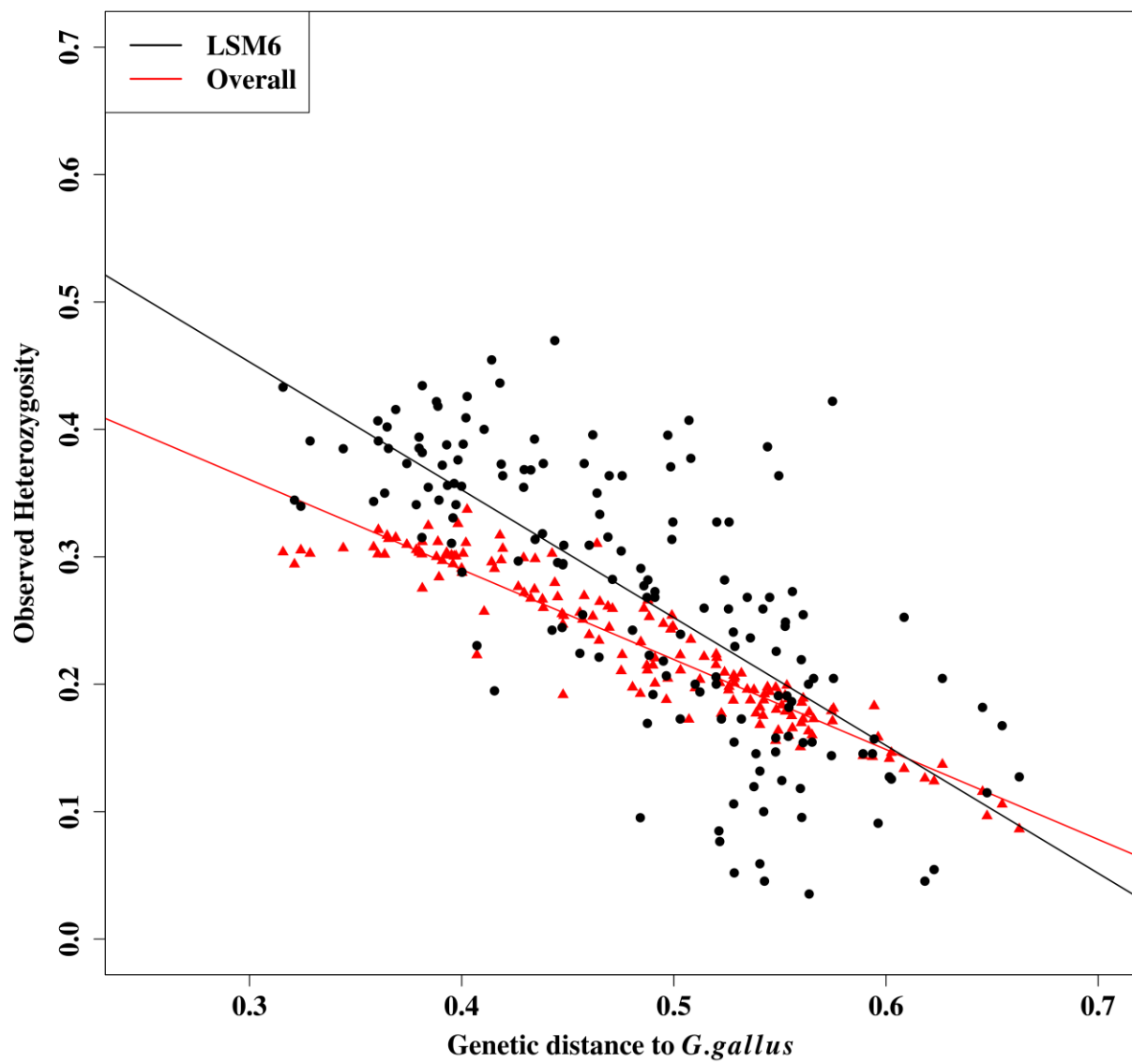

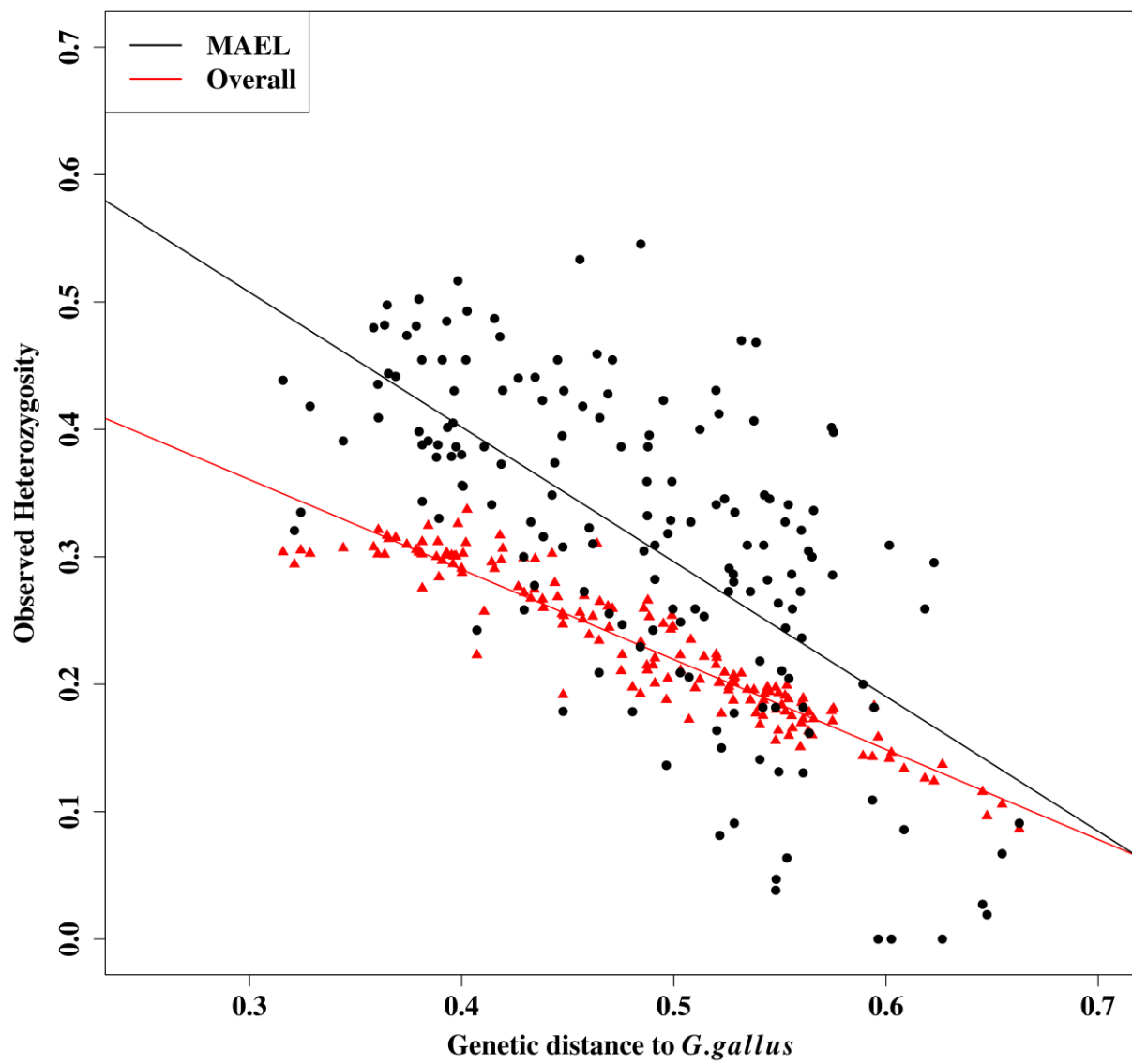

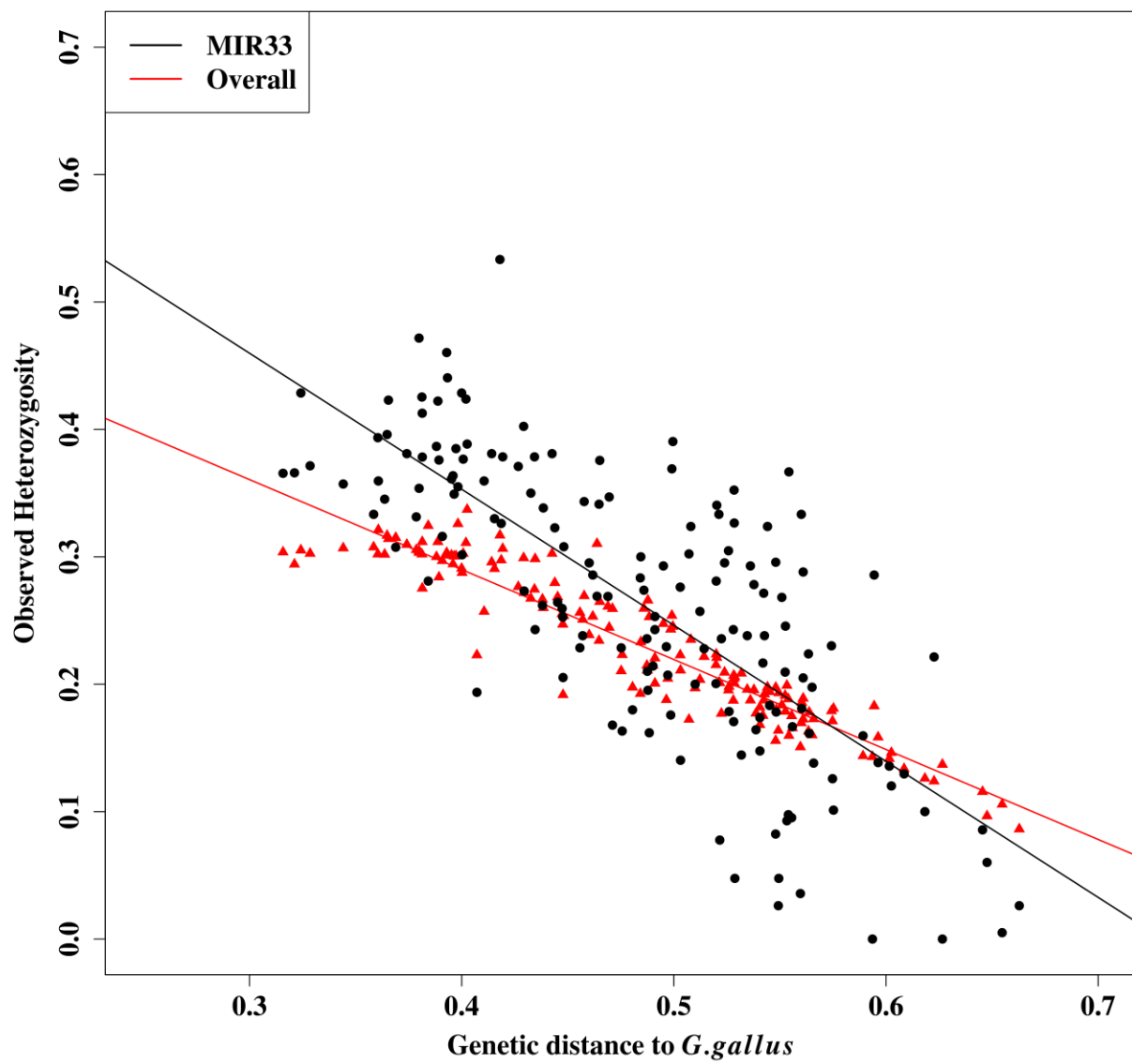

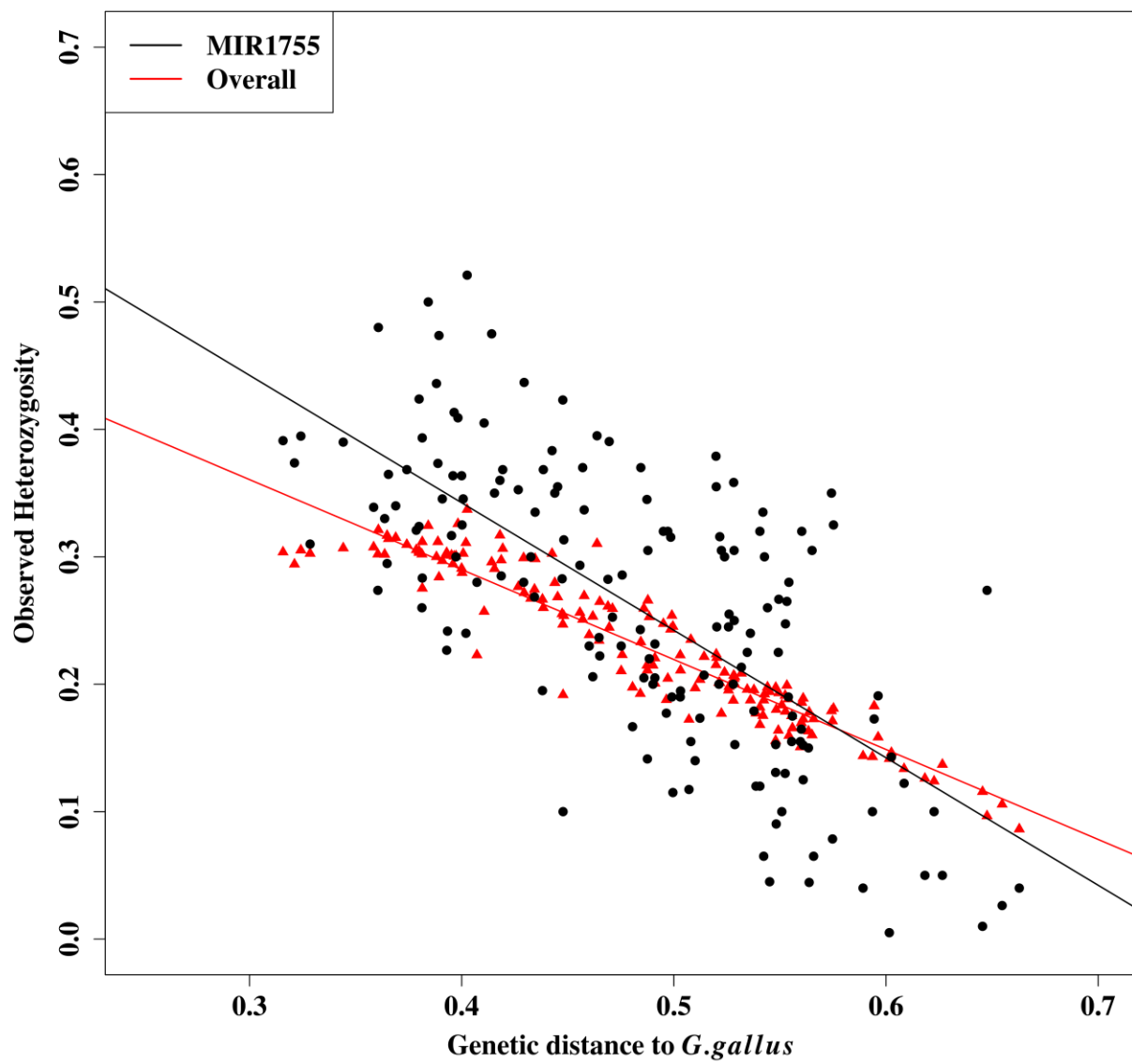

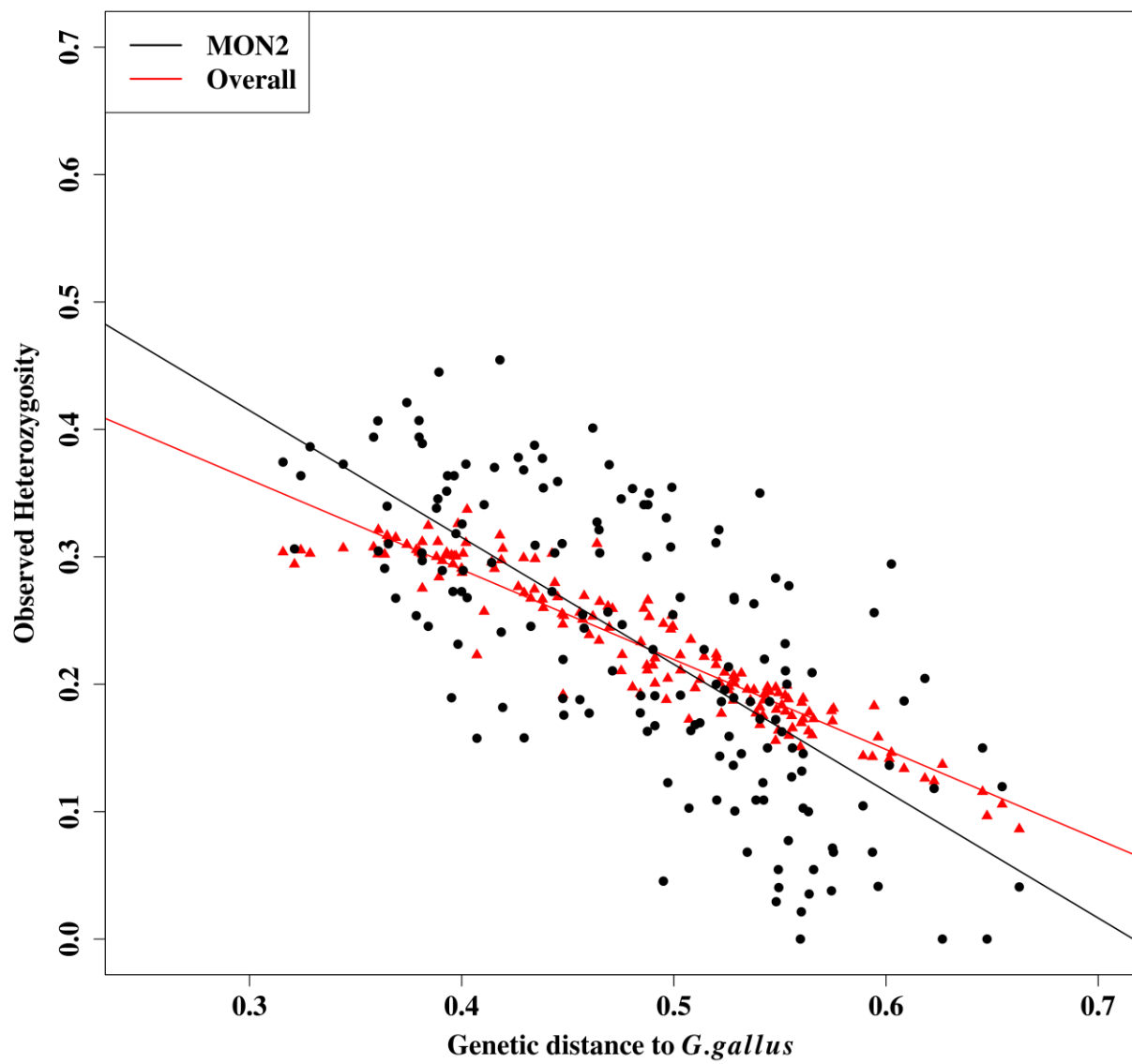

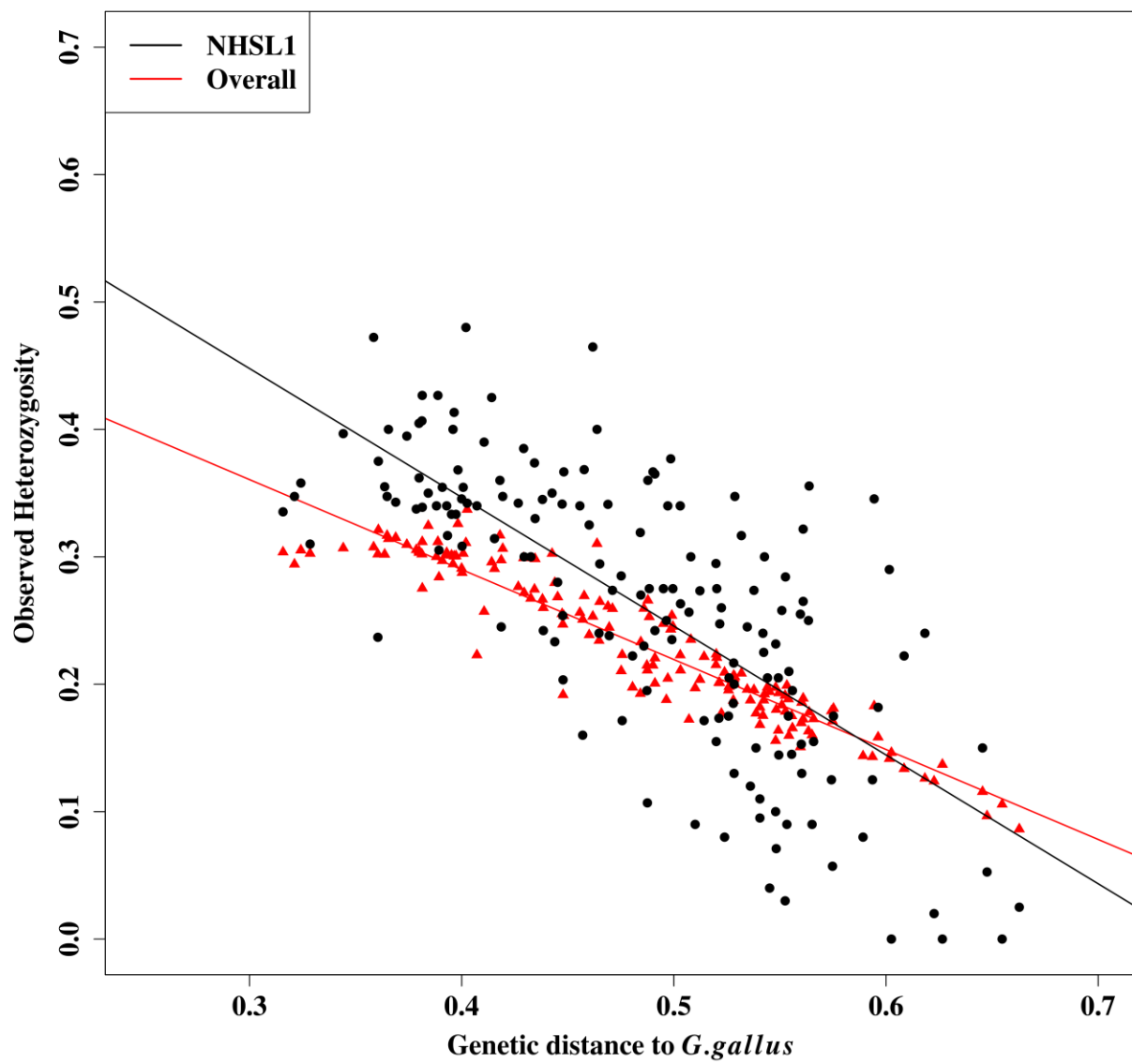

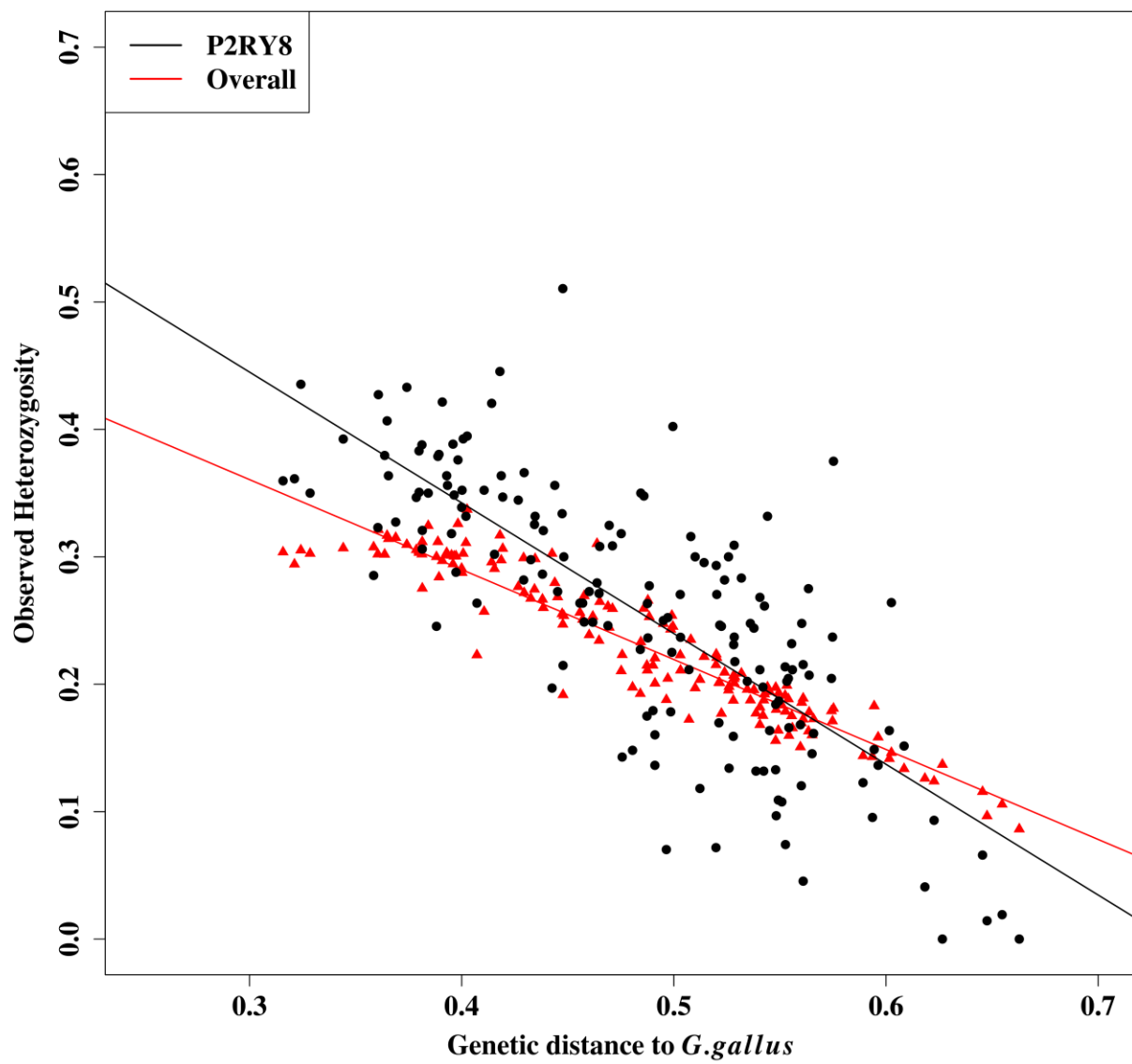

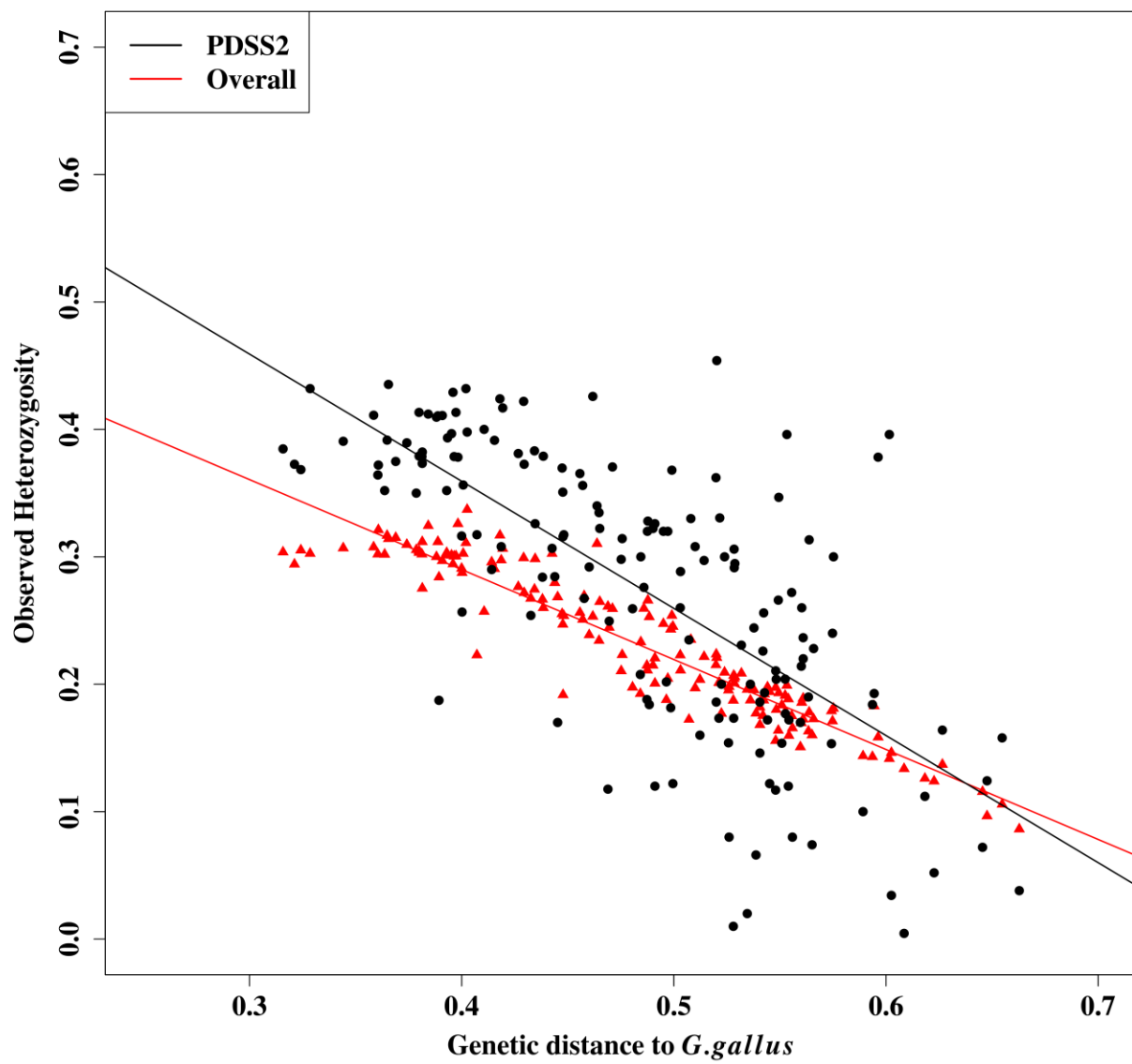

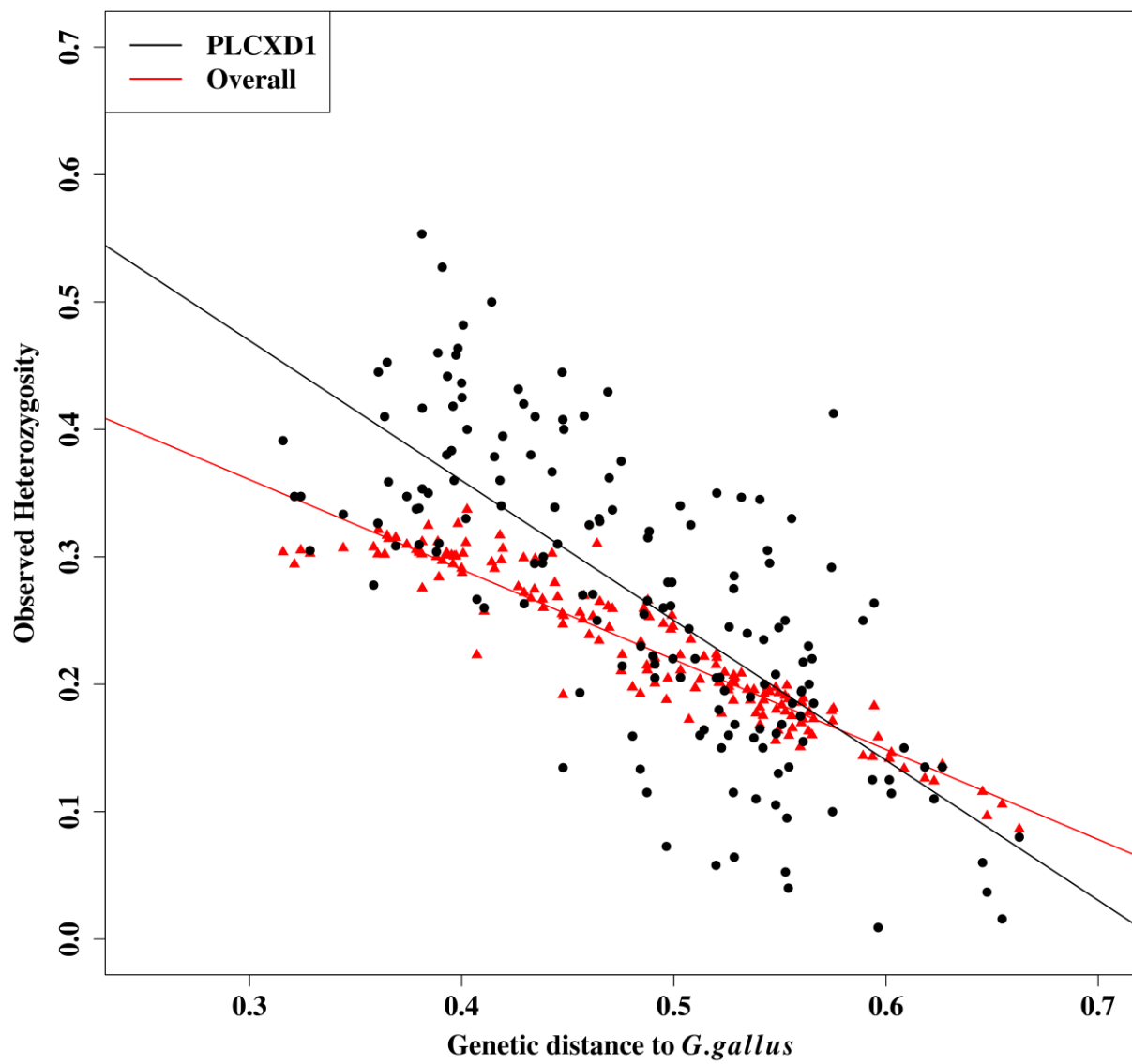

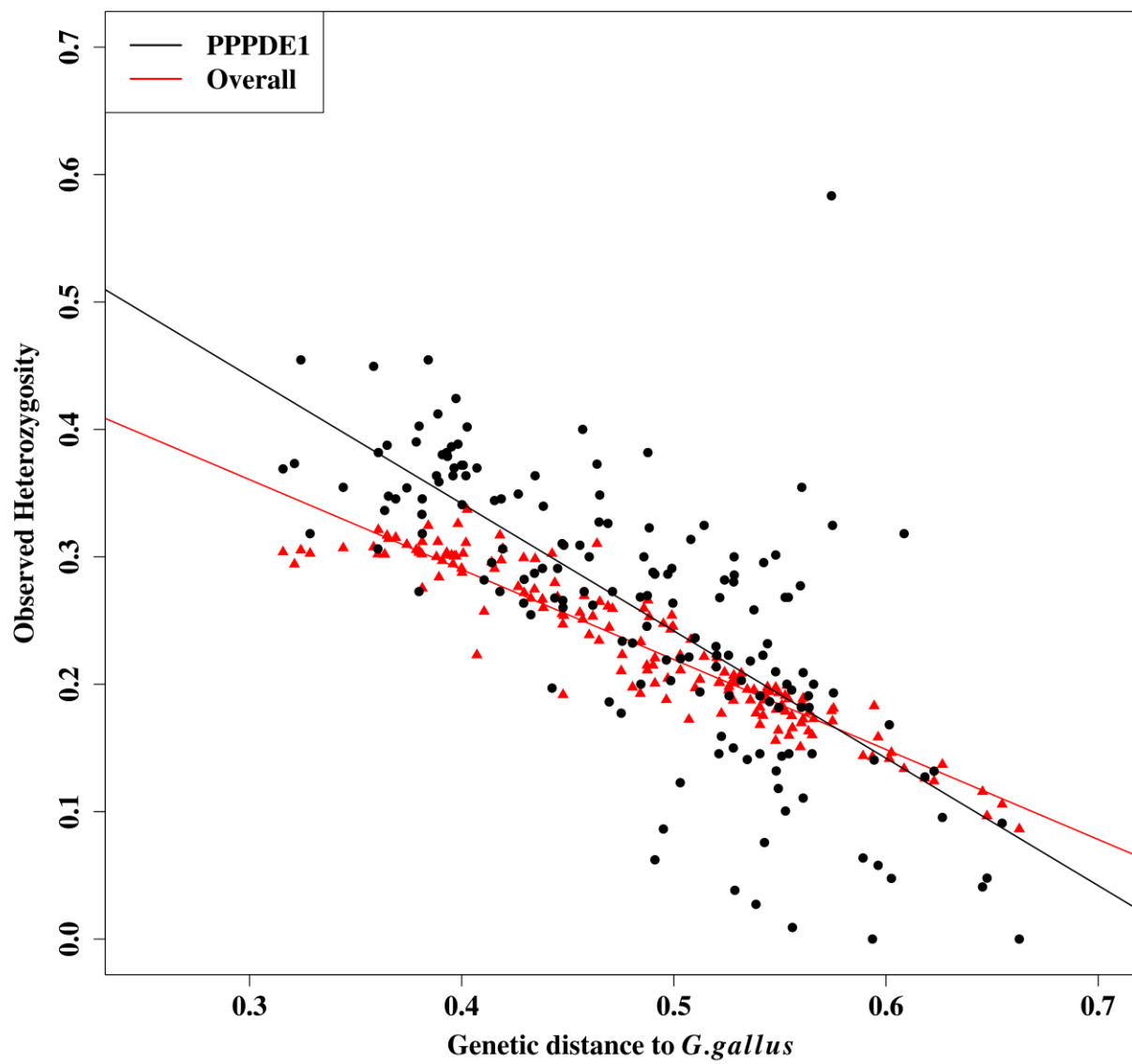

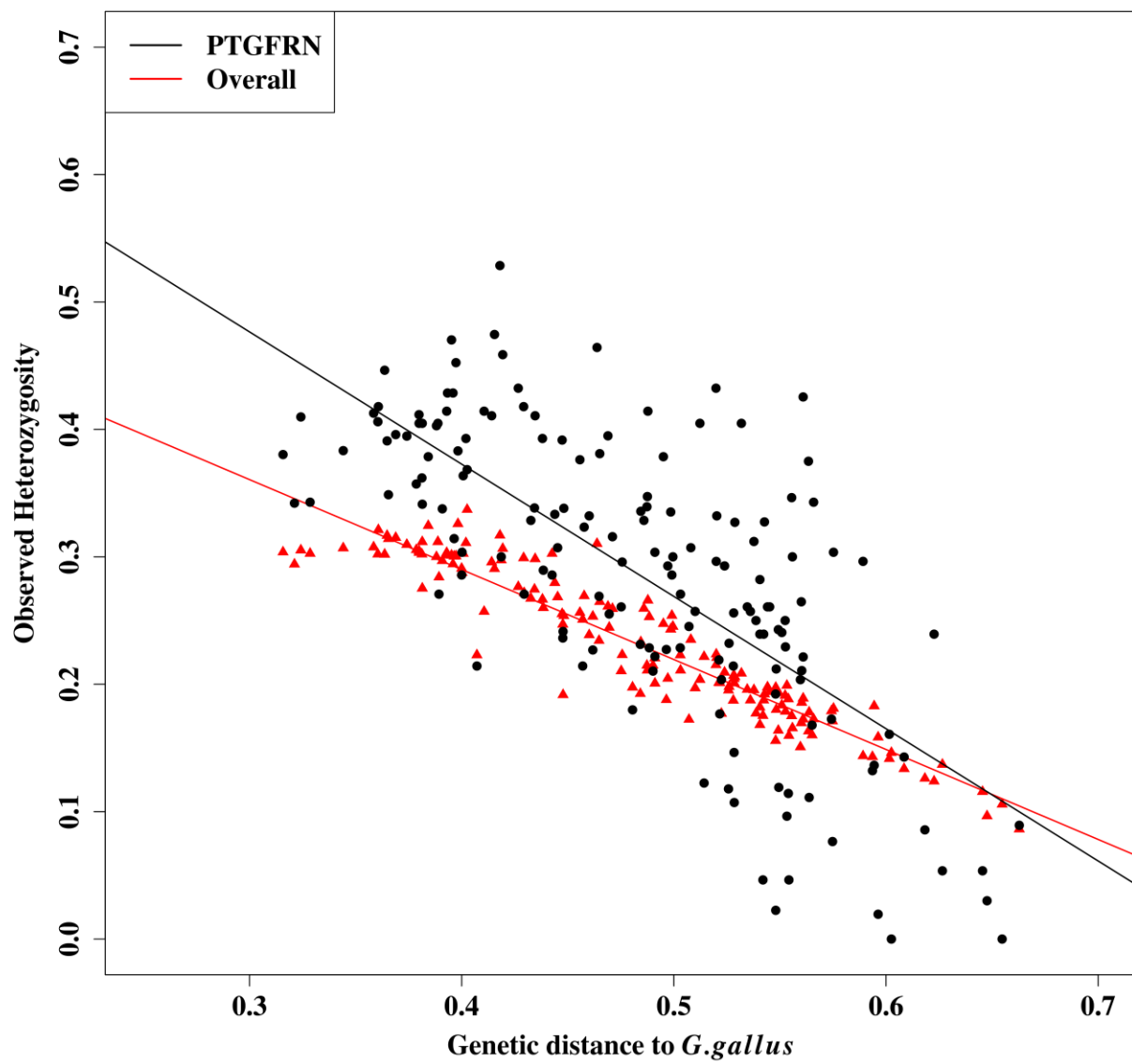

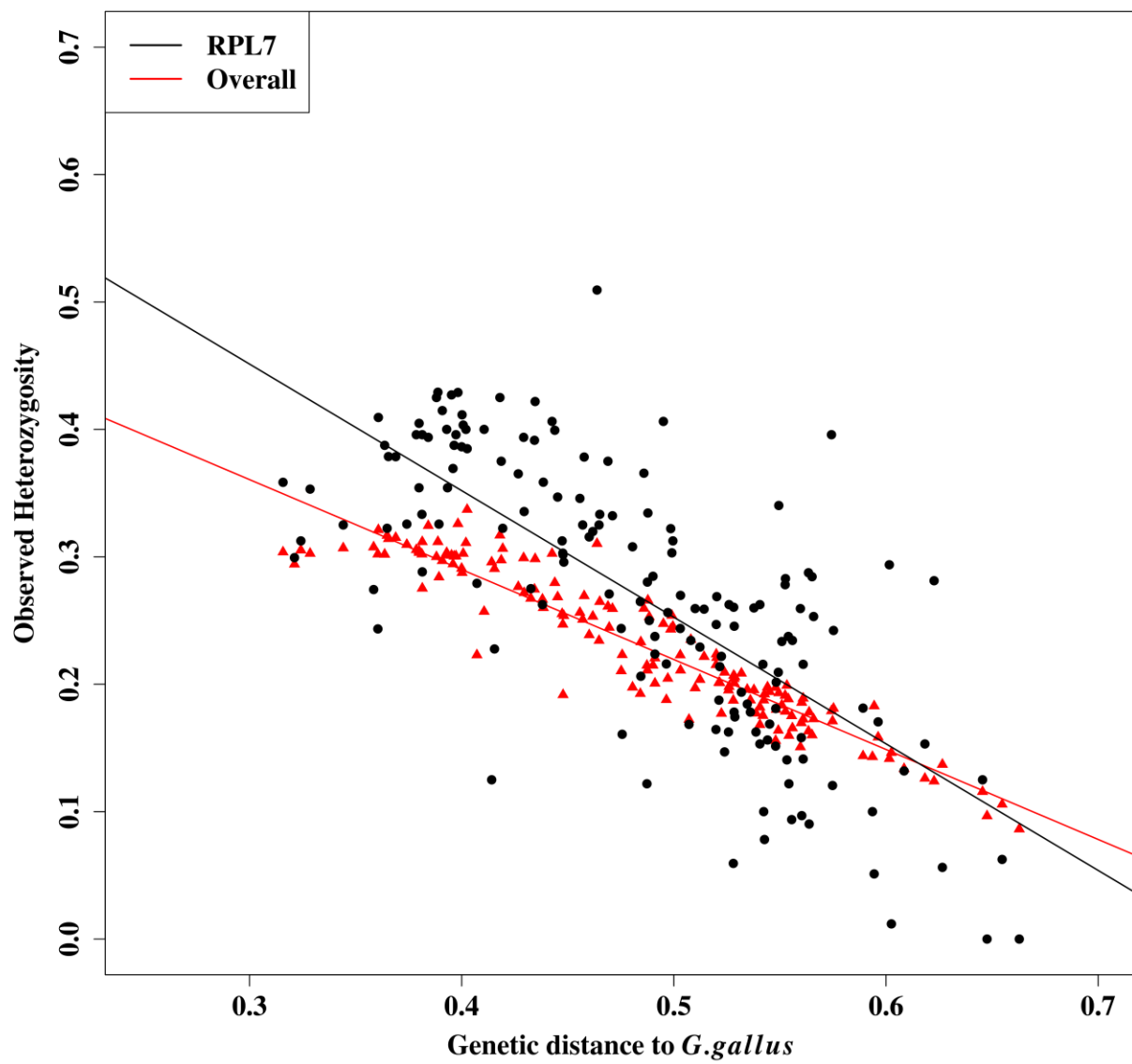

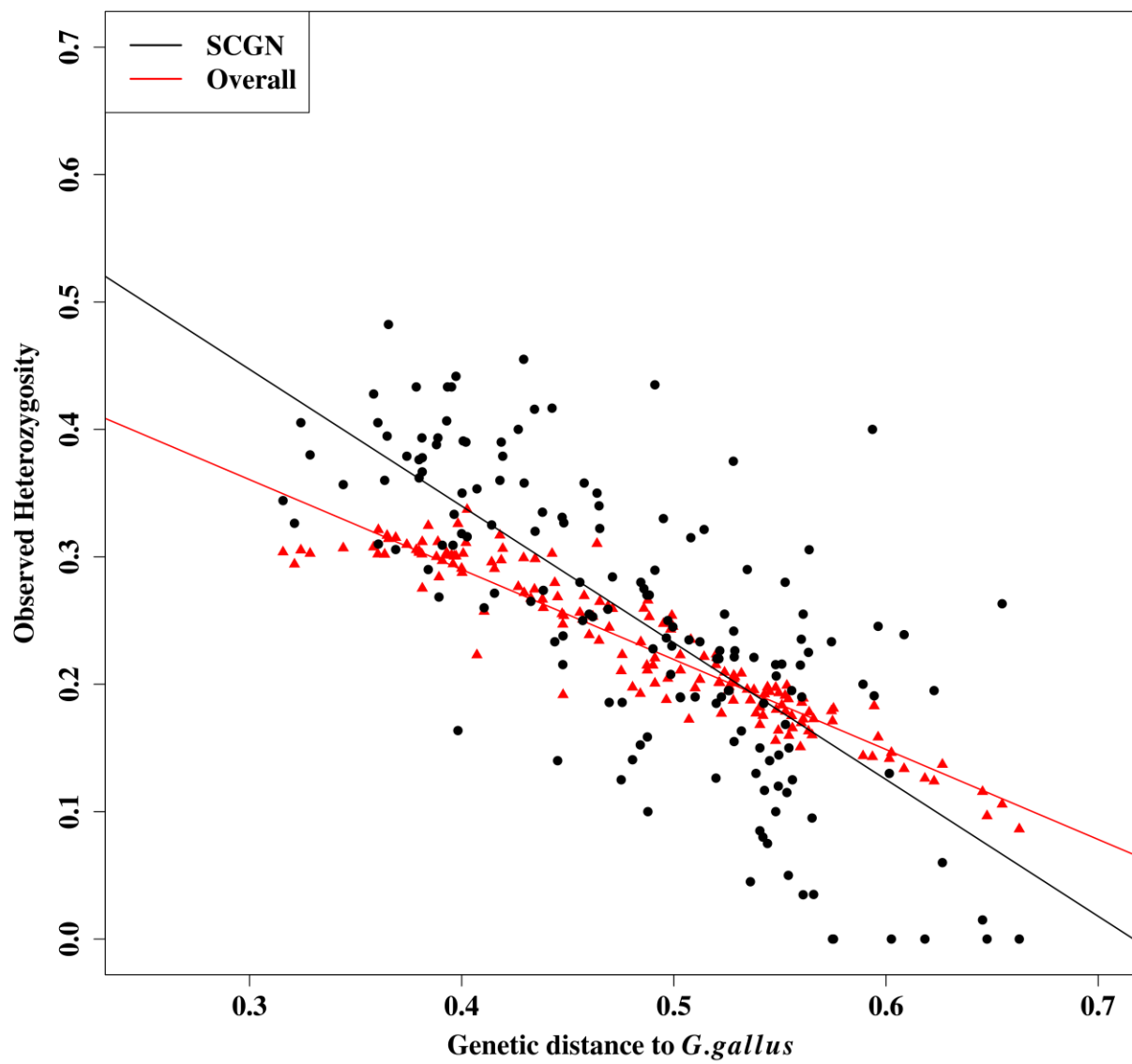

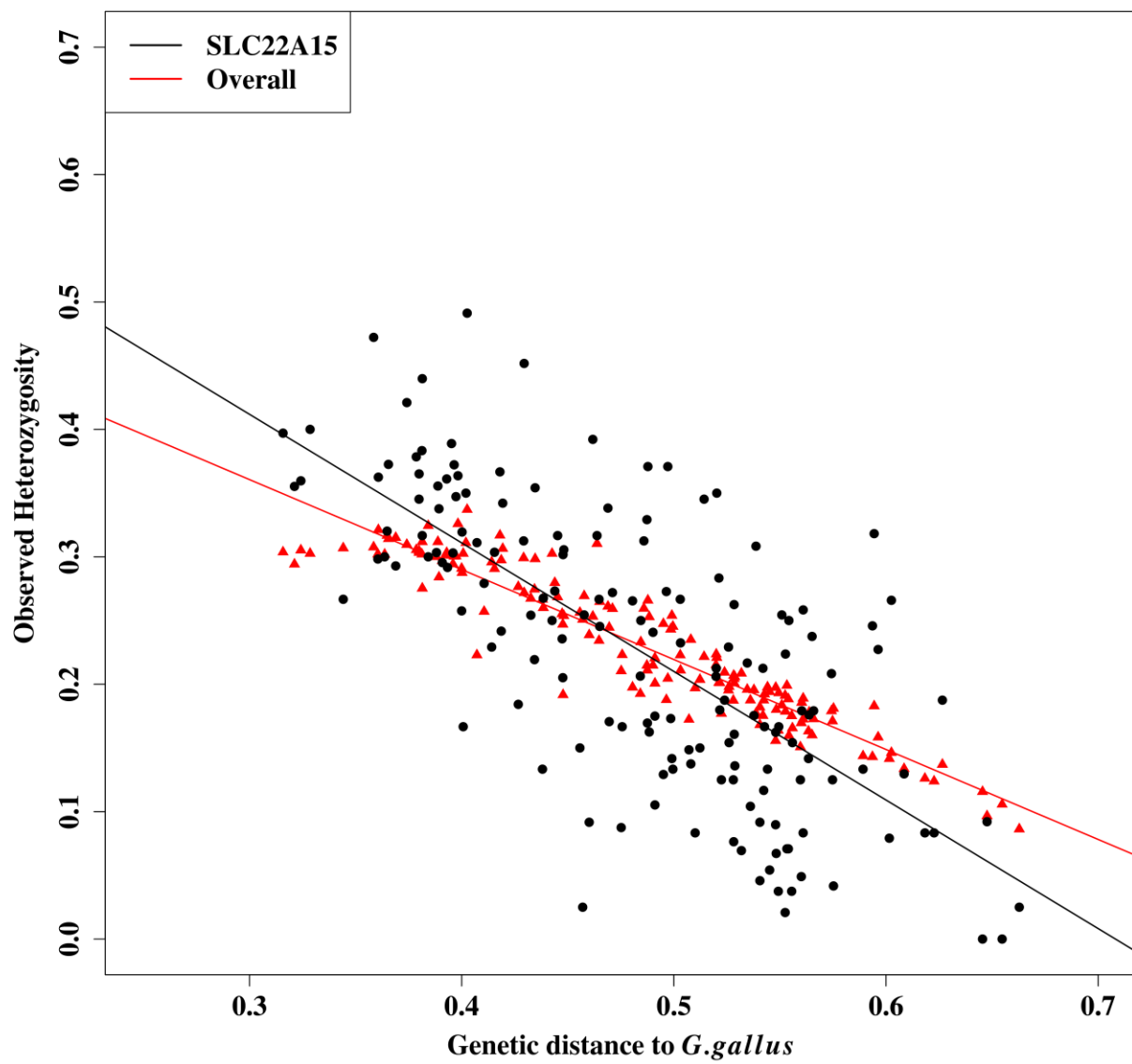

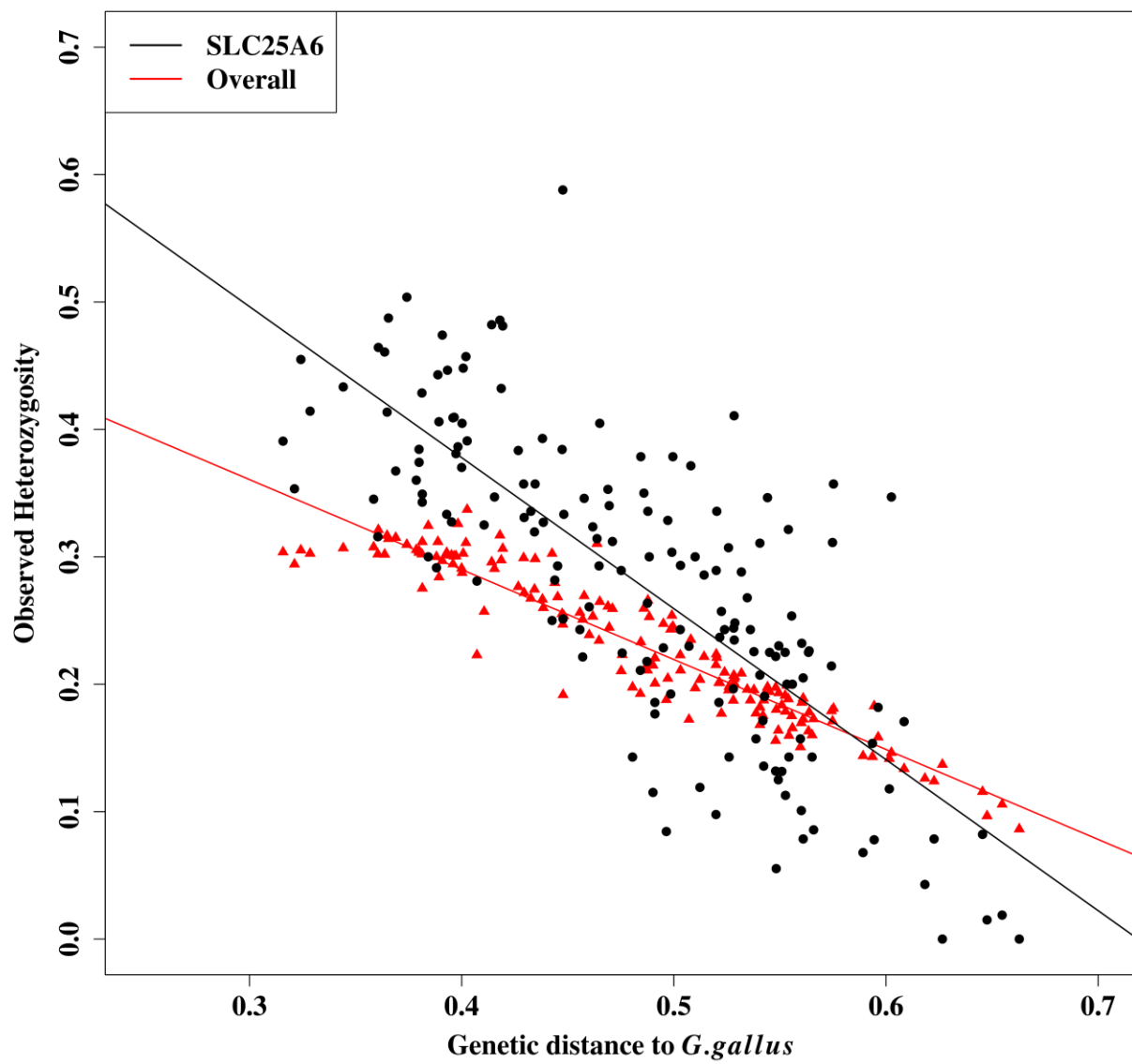

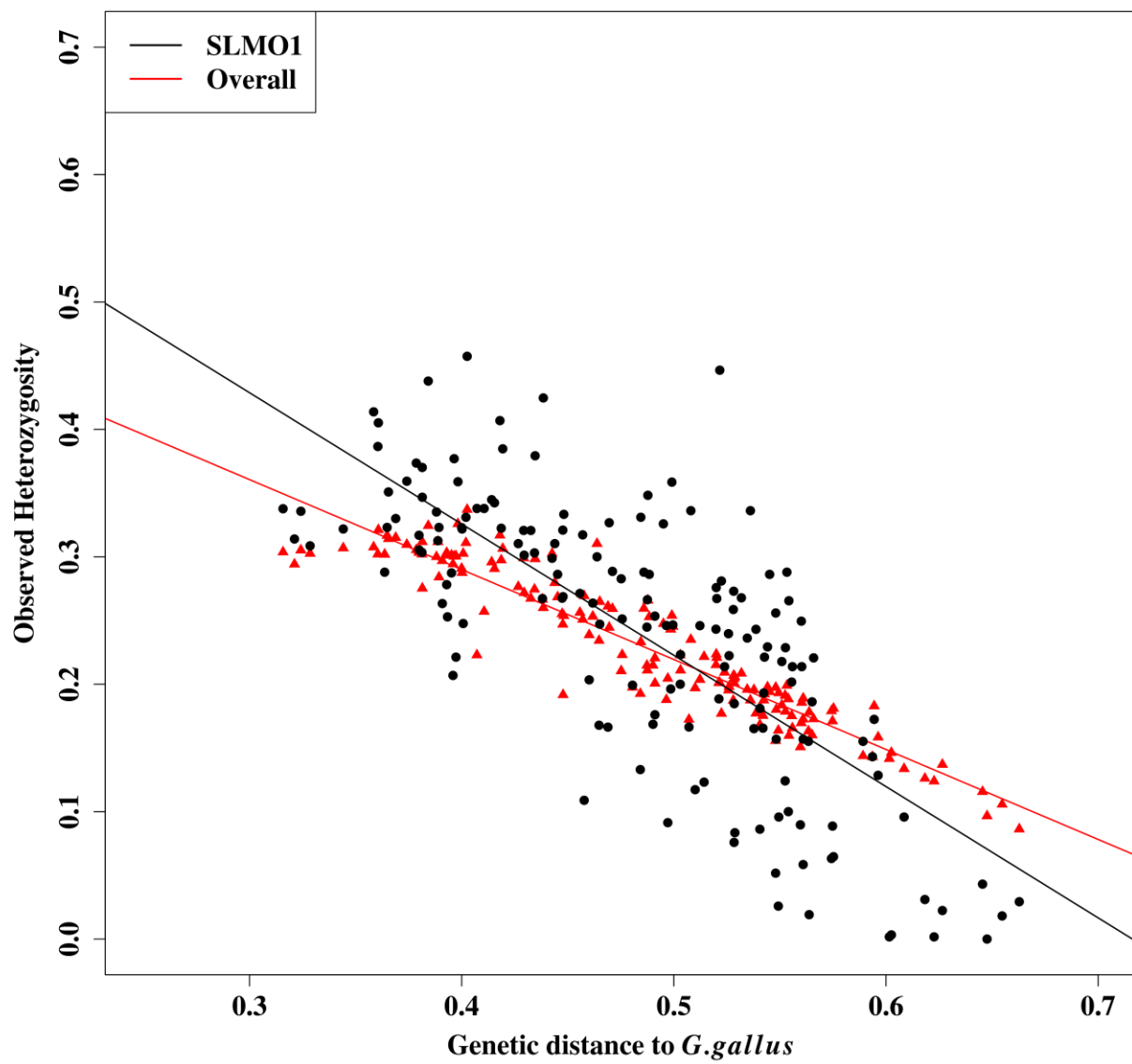

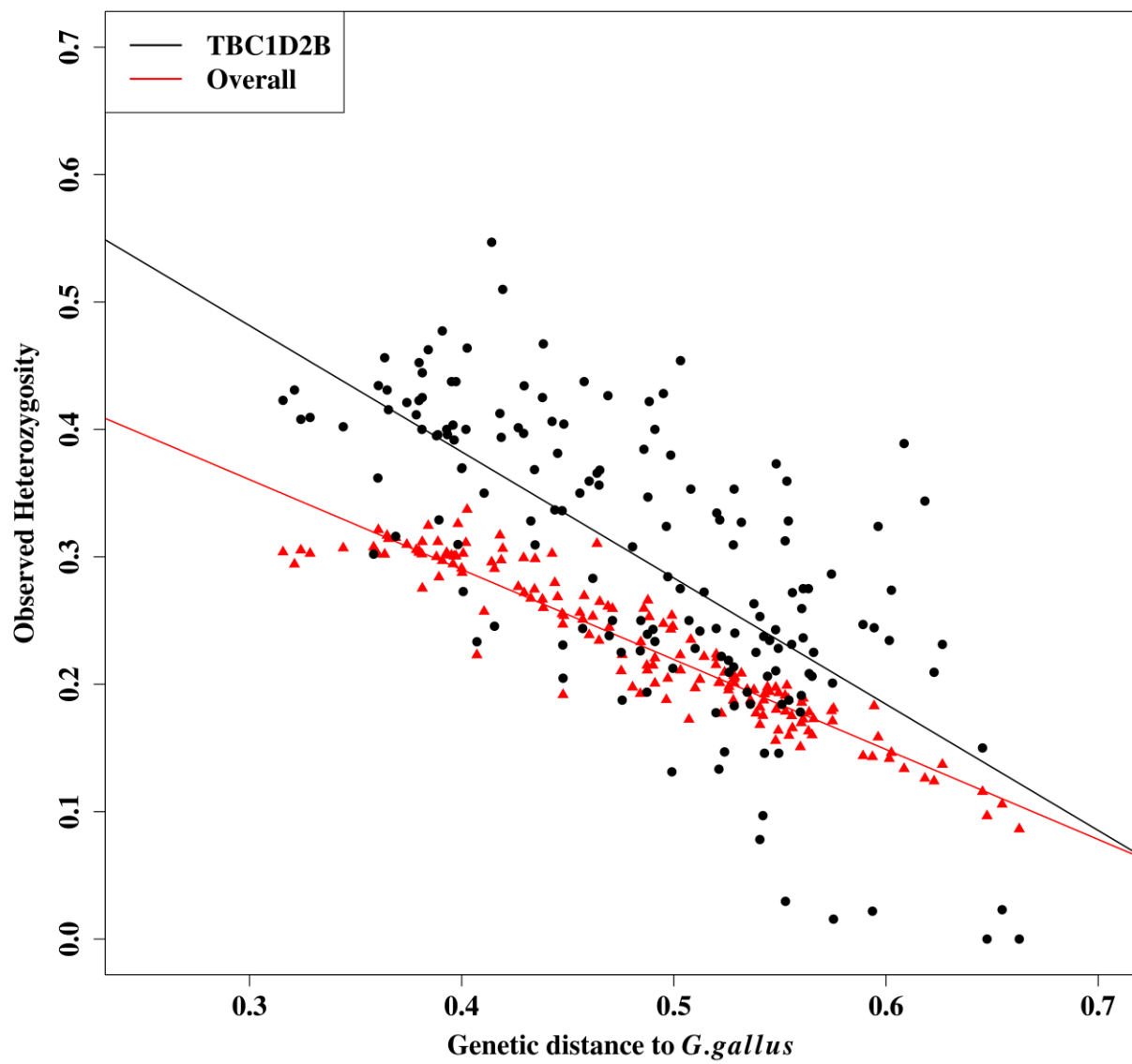

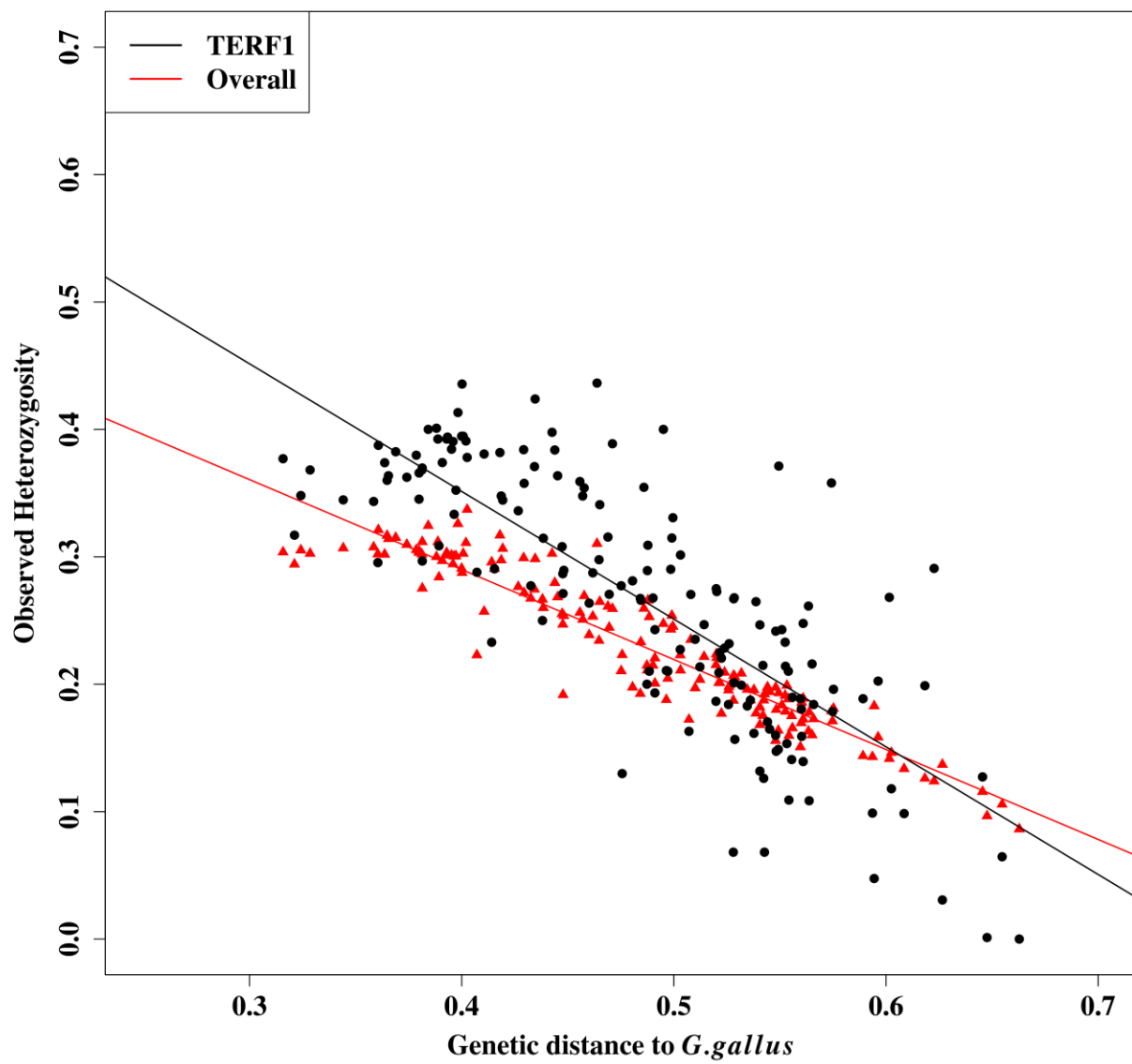

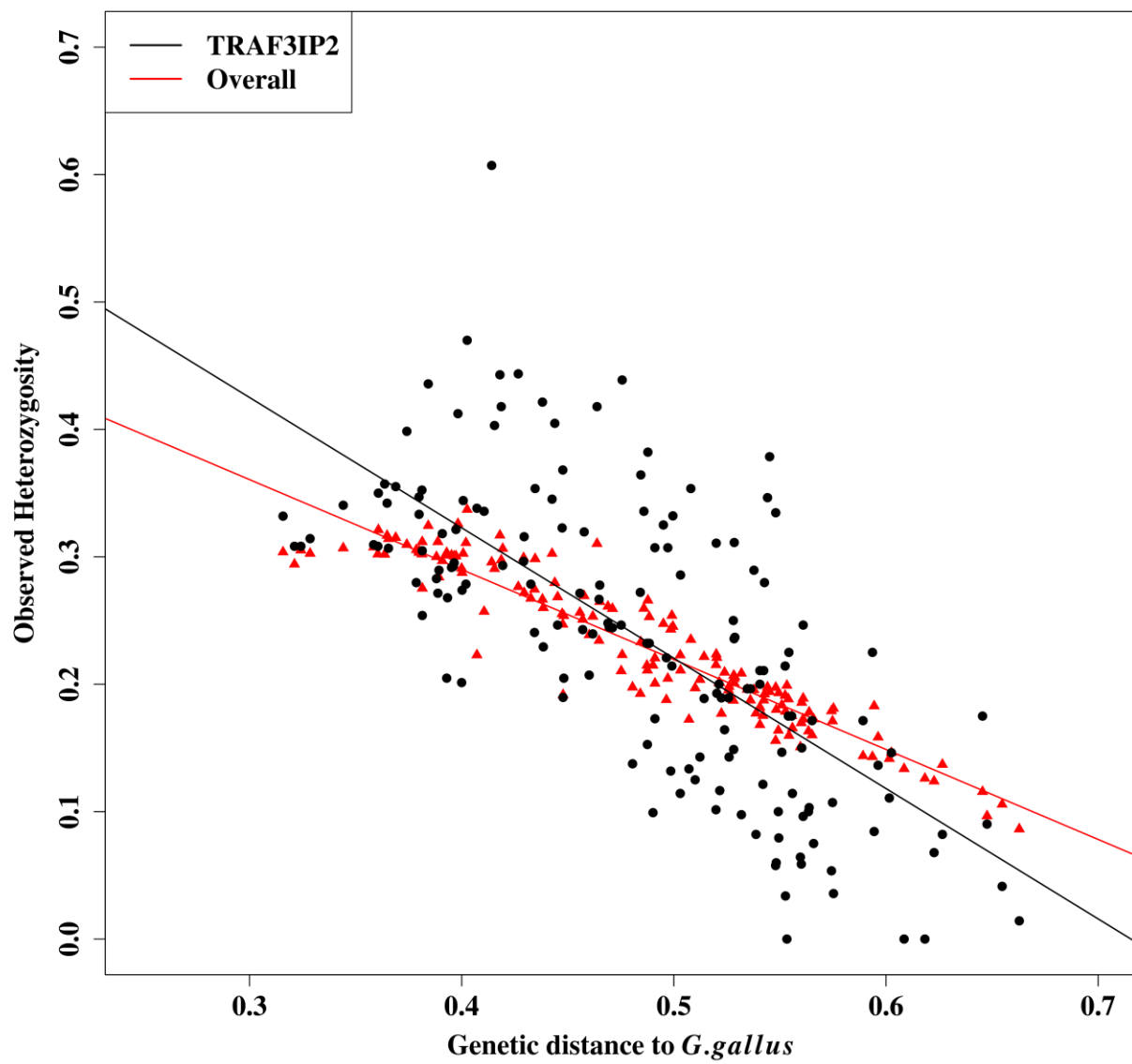

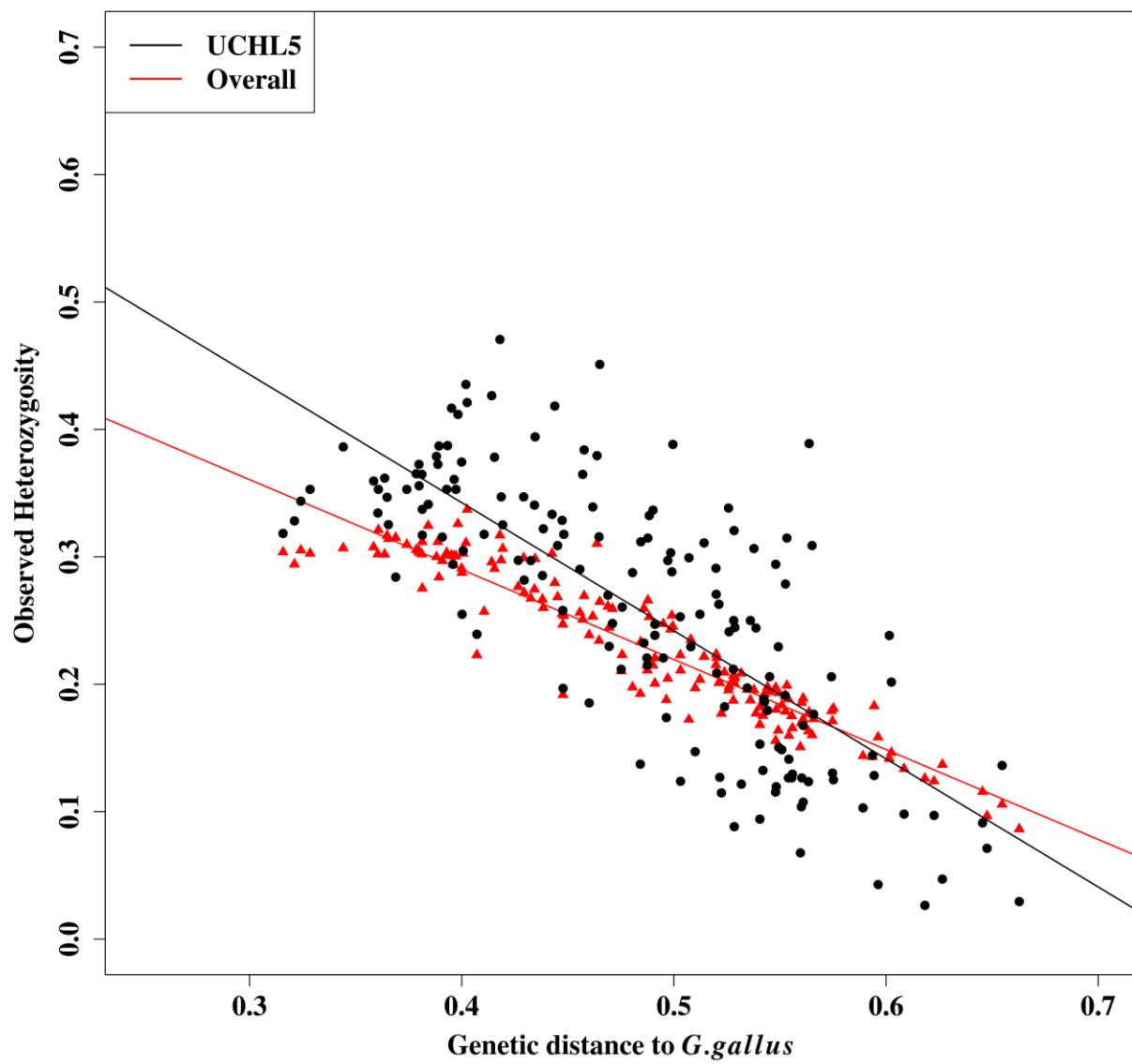

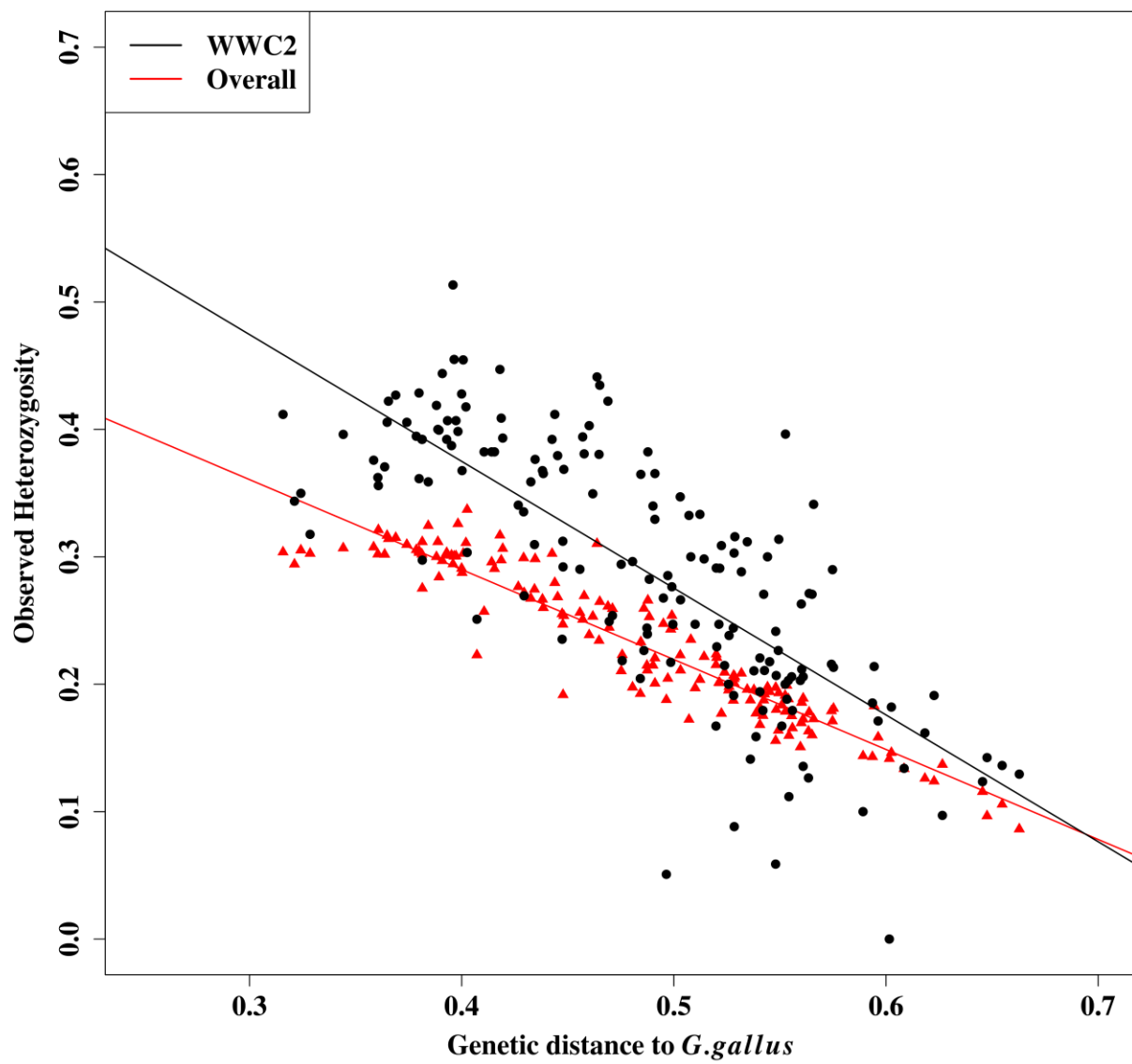

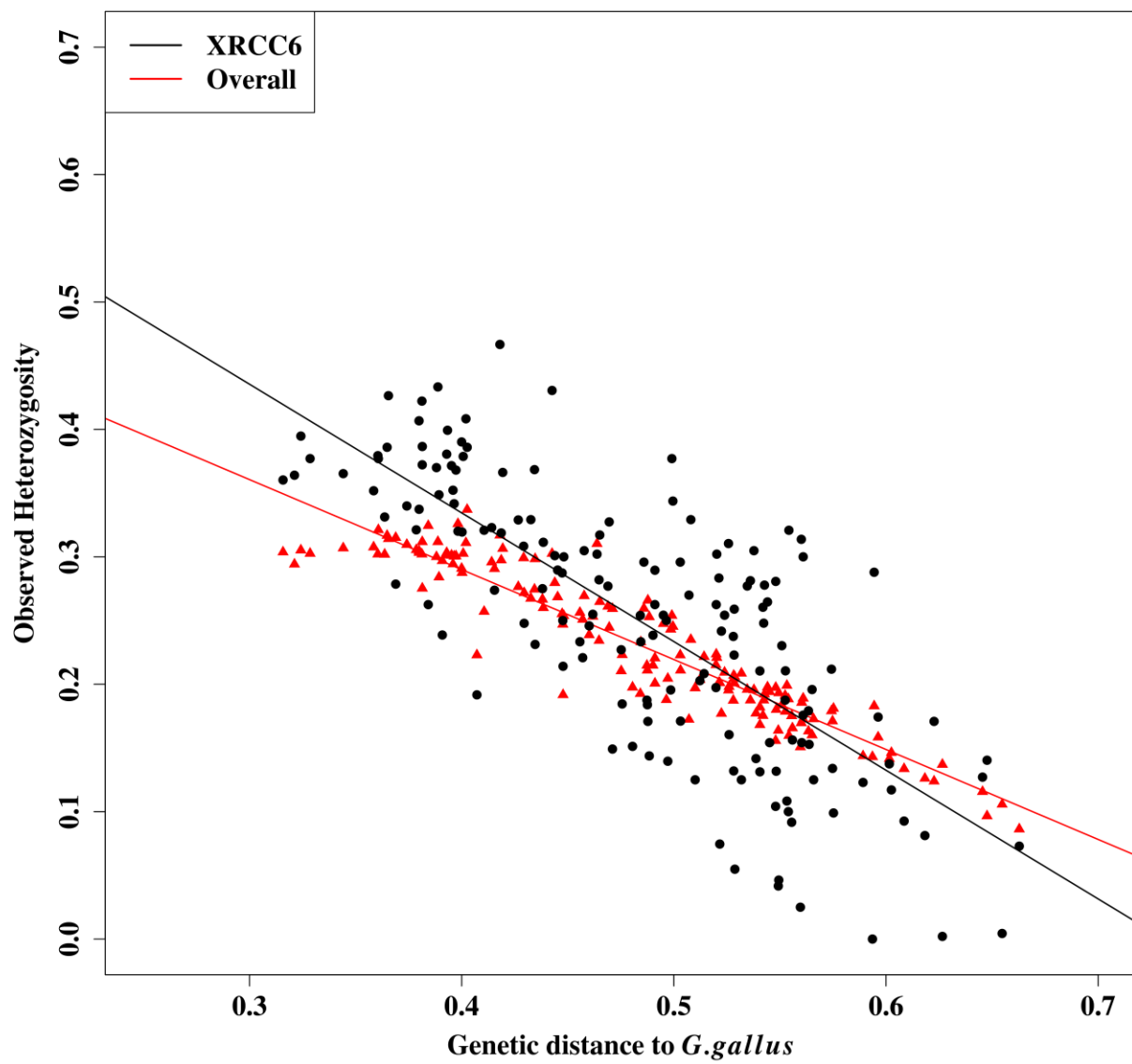

Supplement: Supplementary file 4 — Additional file 4. Relationships between observed heterozygosity and genetic distance to G. gallus for genes in the top 5% slope range. [file 12711_2021_628_MOESM4_ESM.pdf]

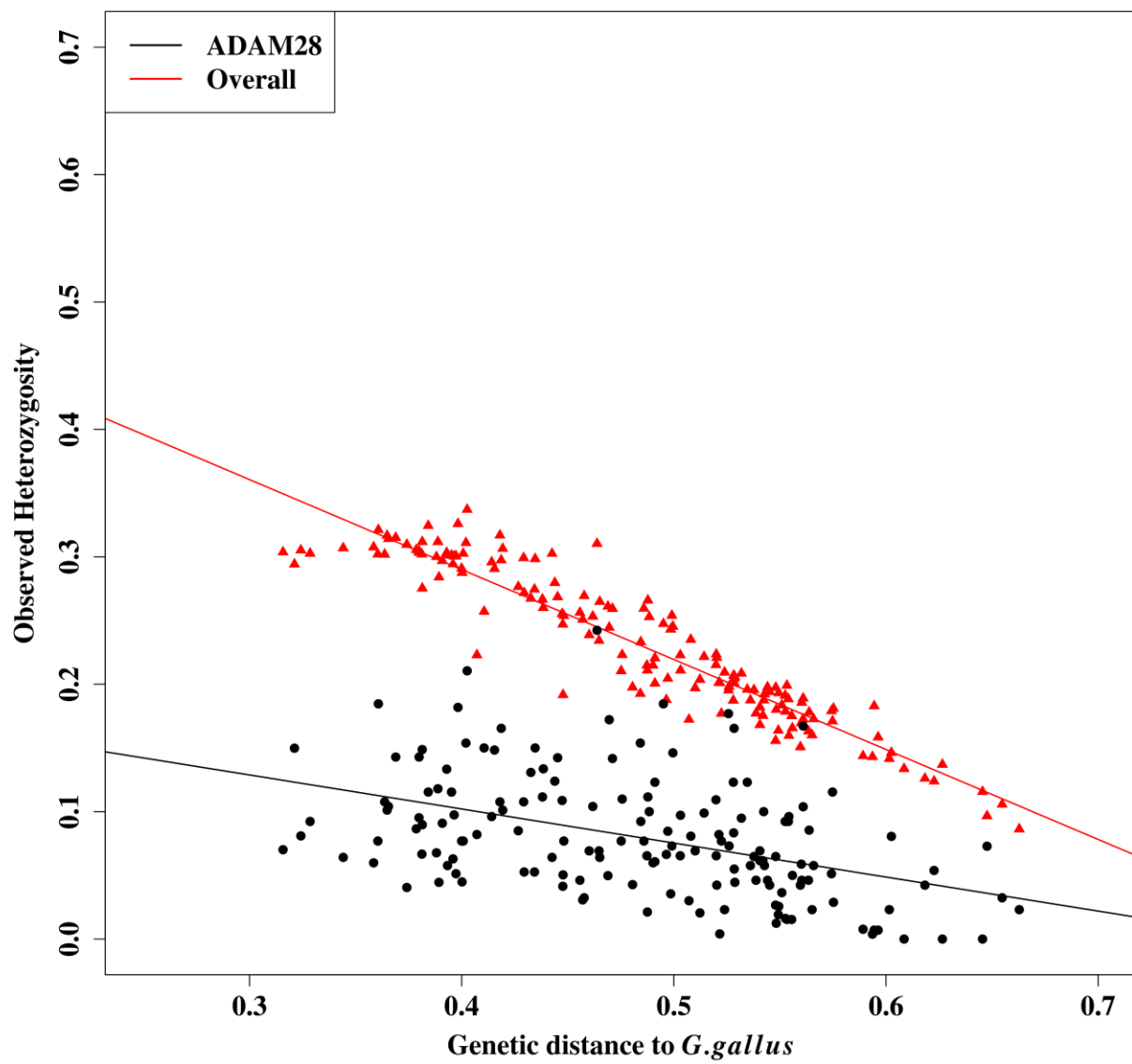

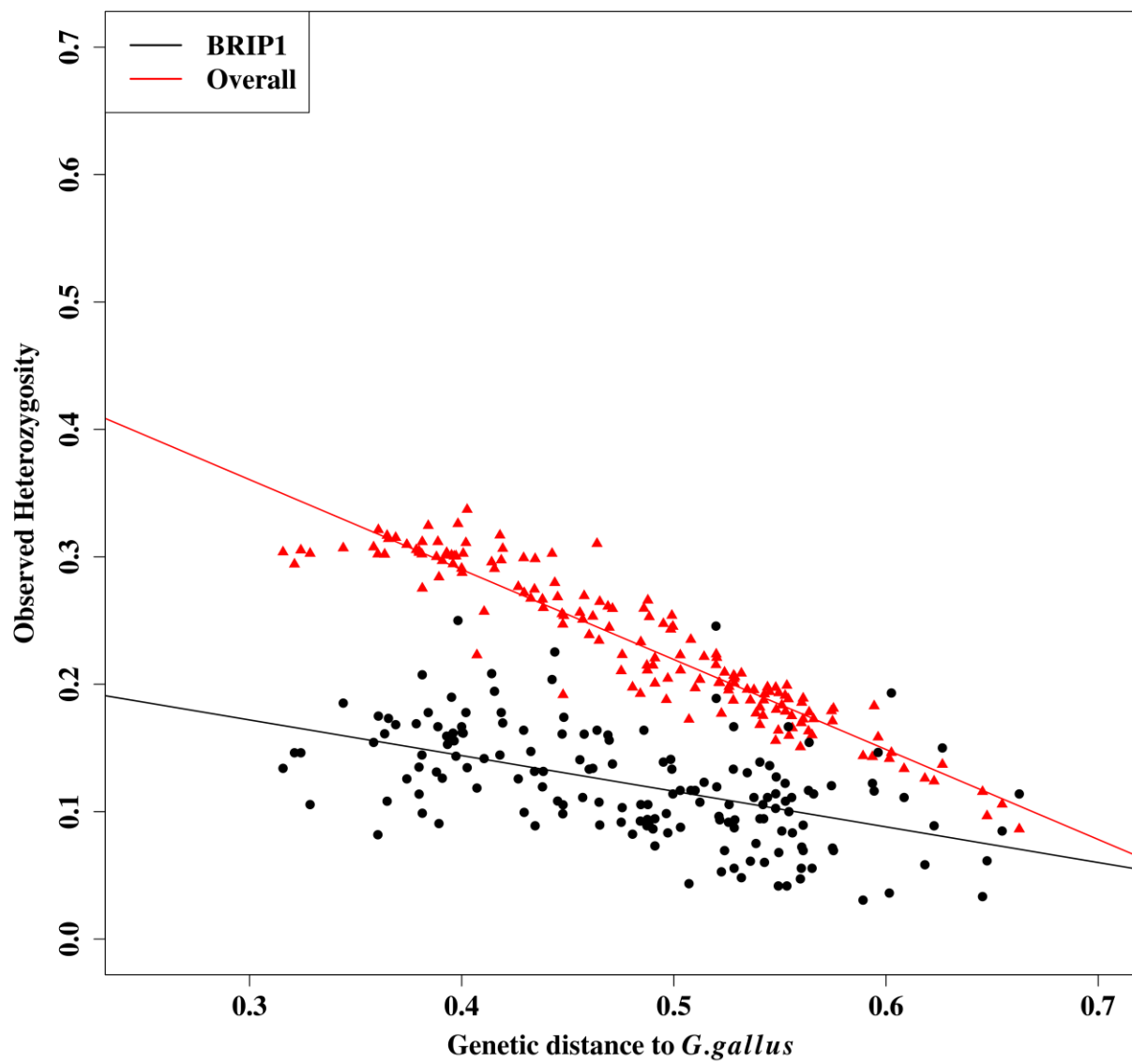

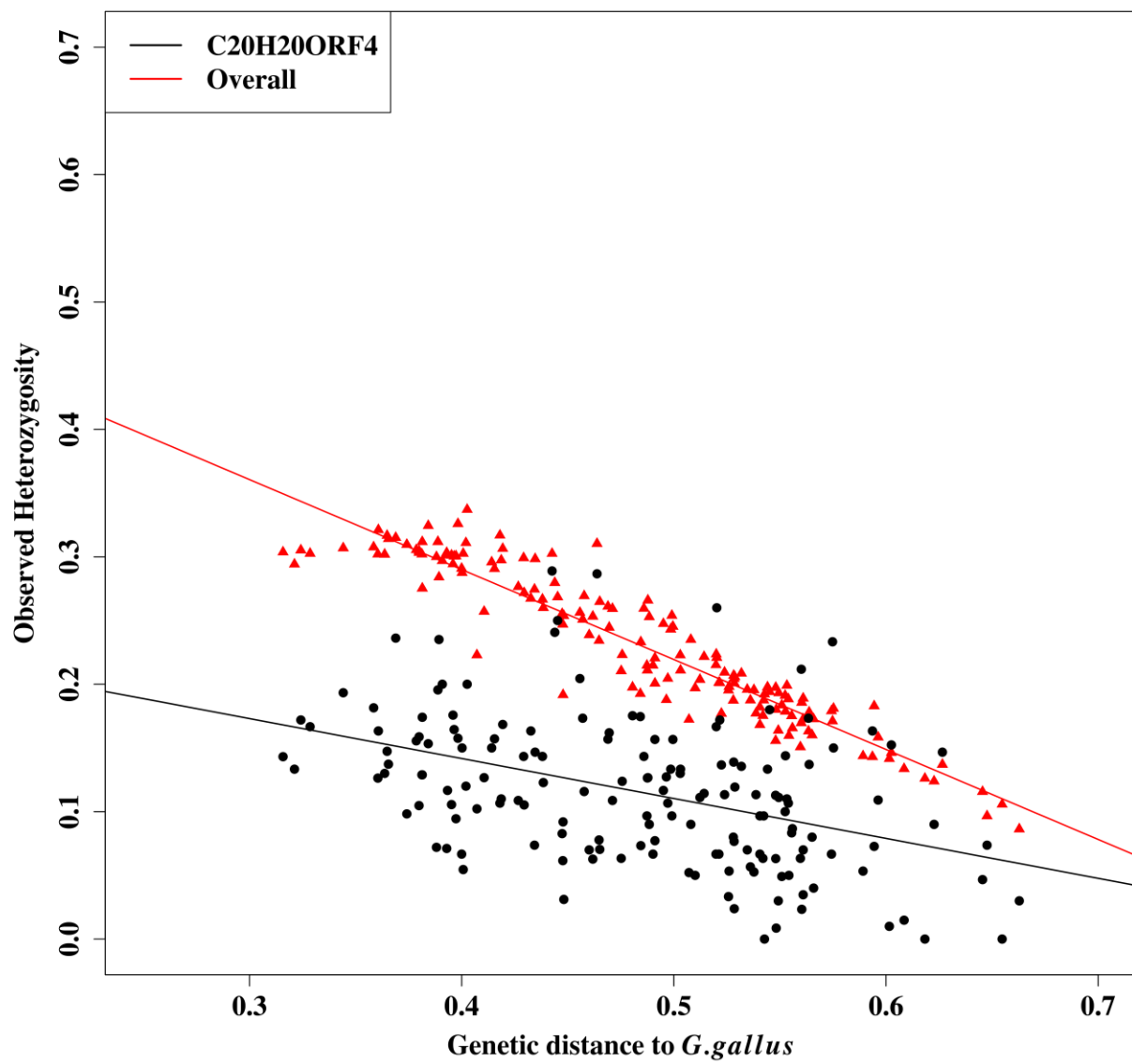

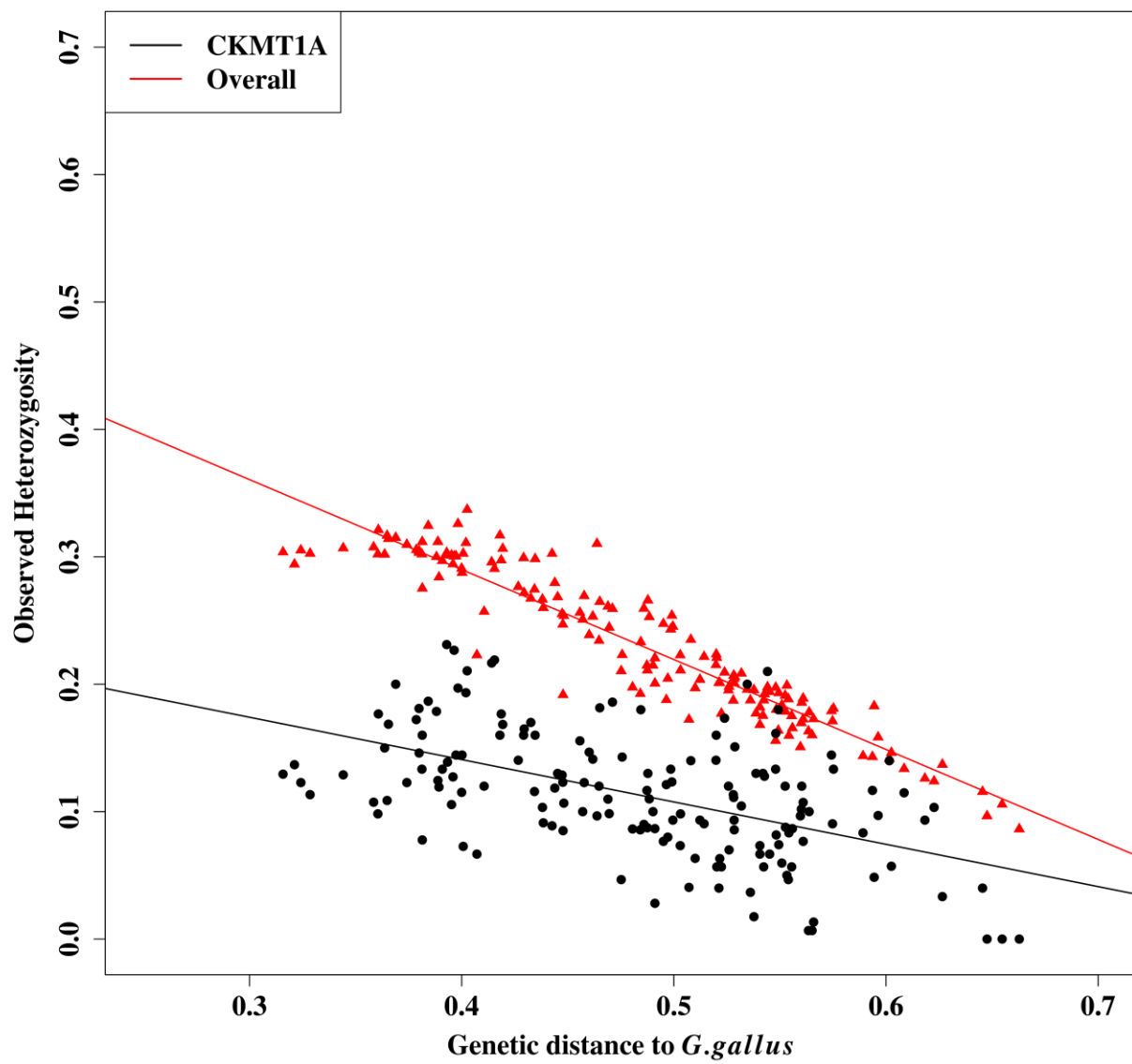

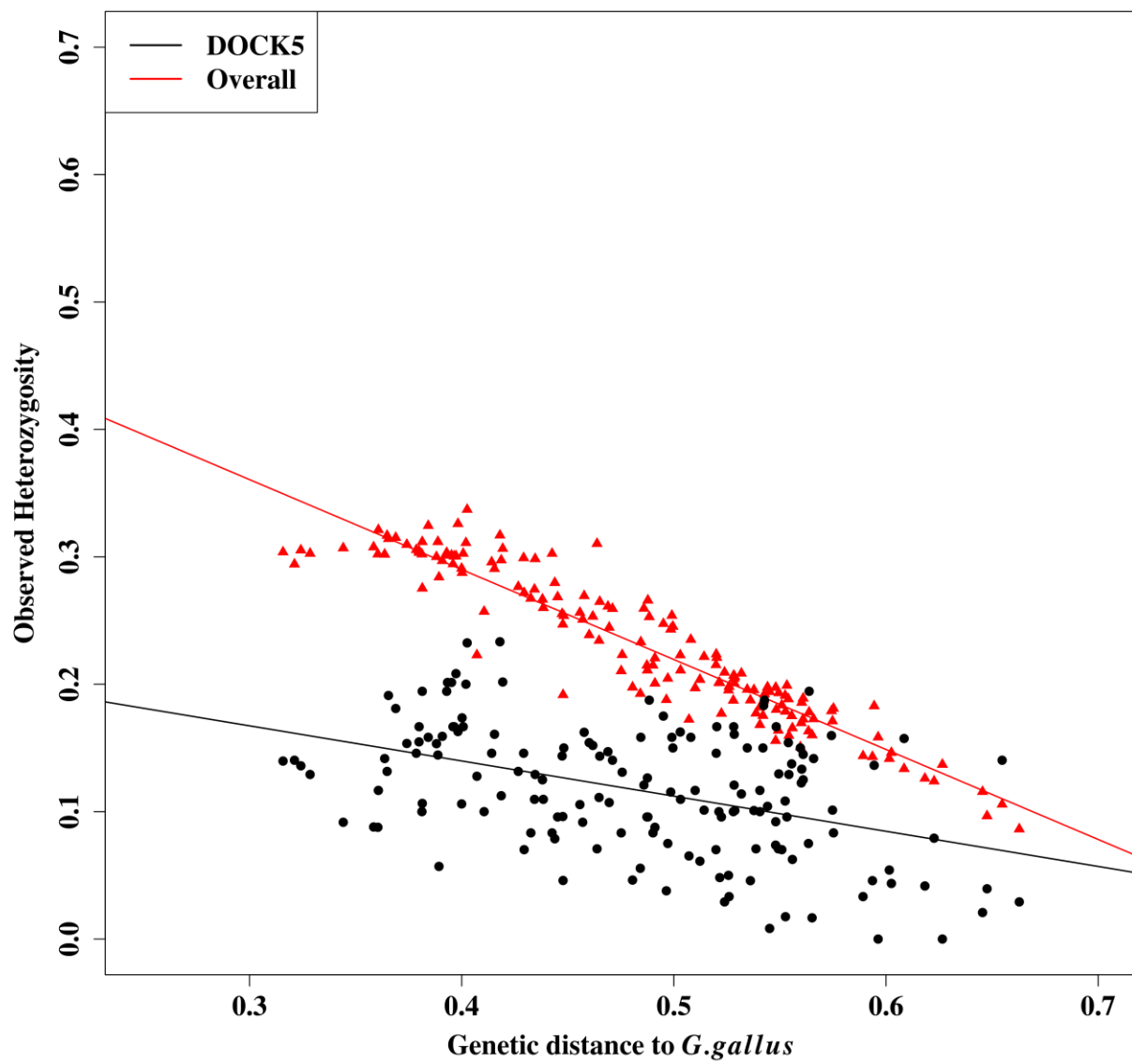

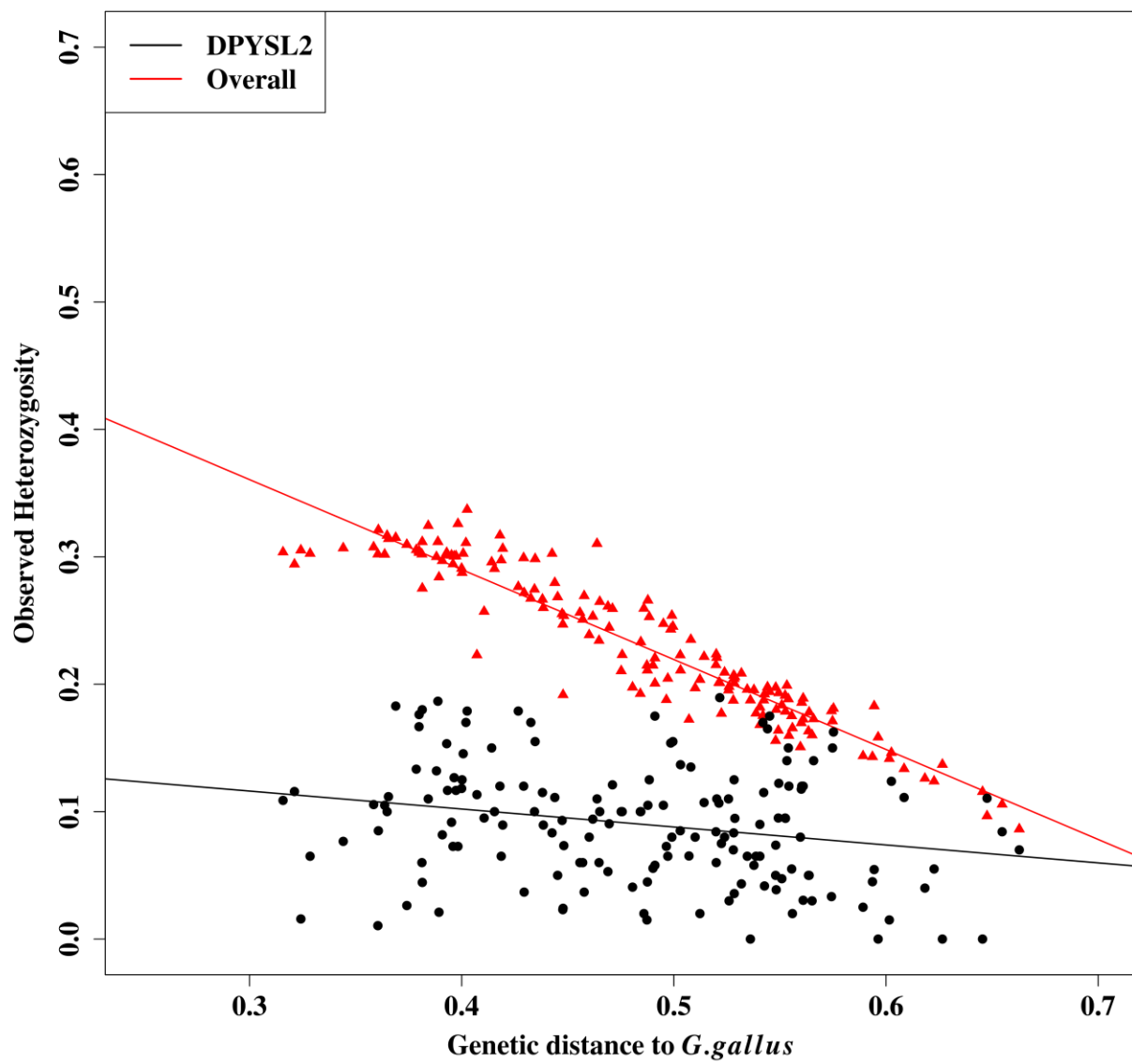

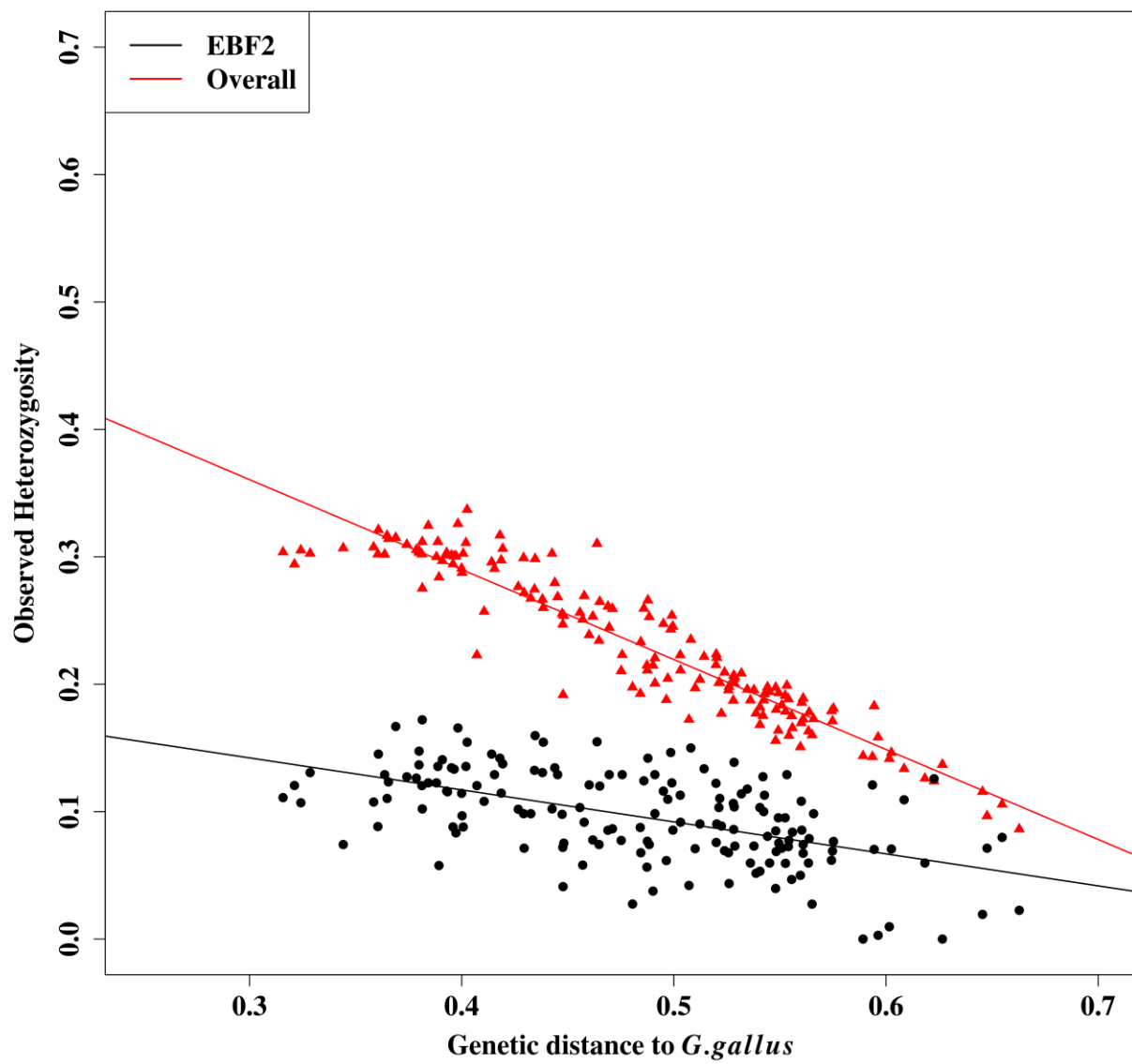

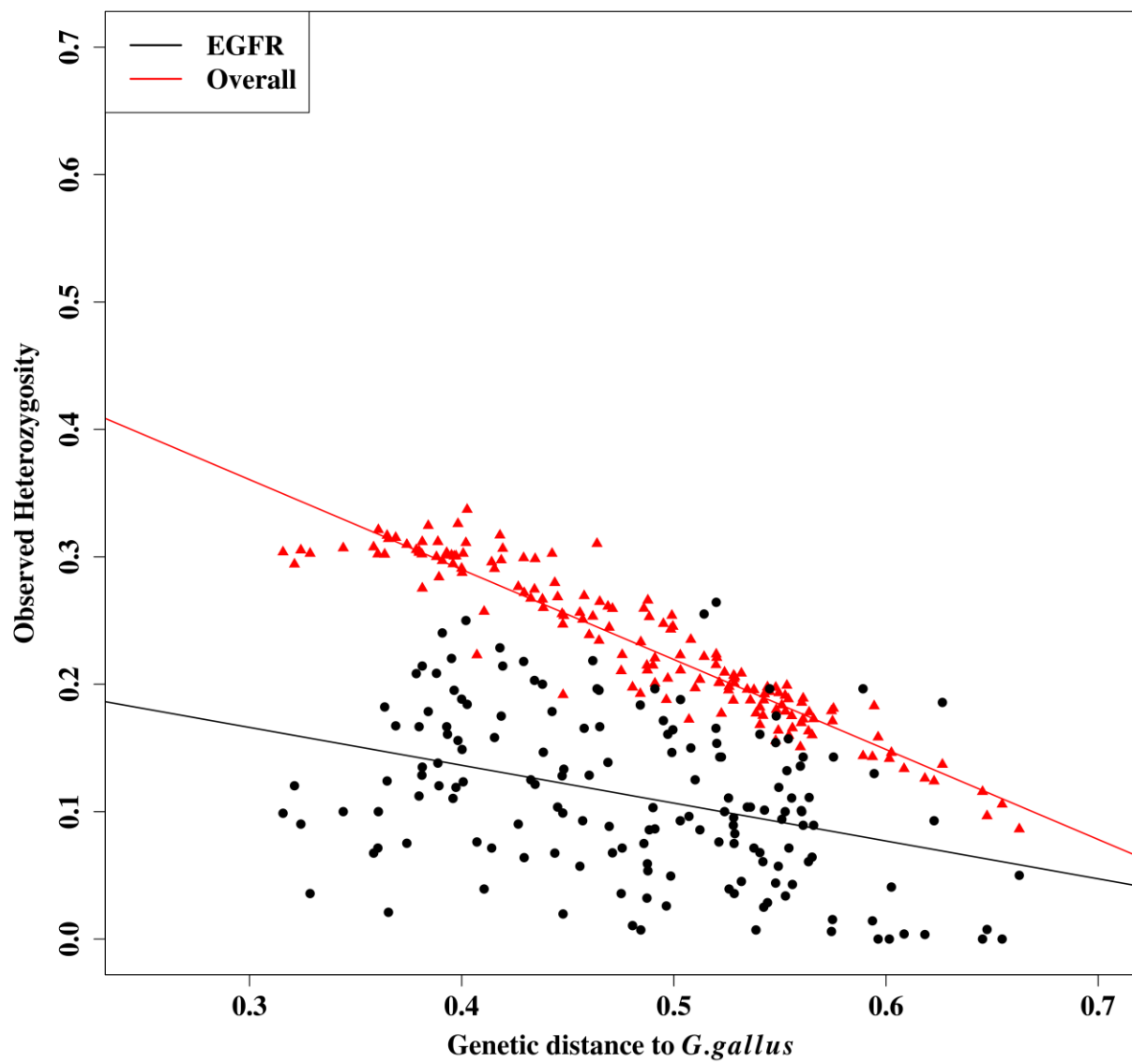

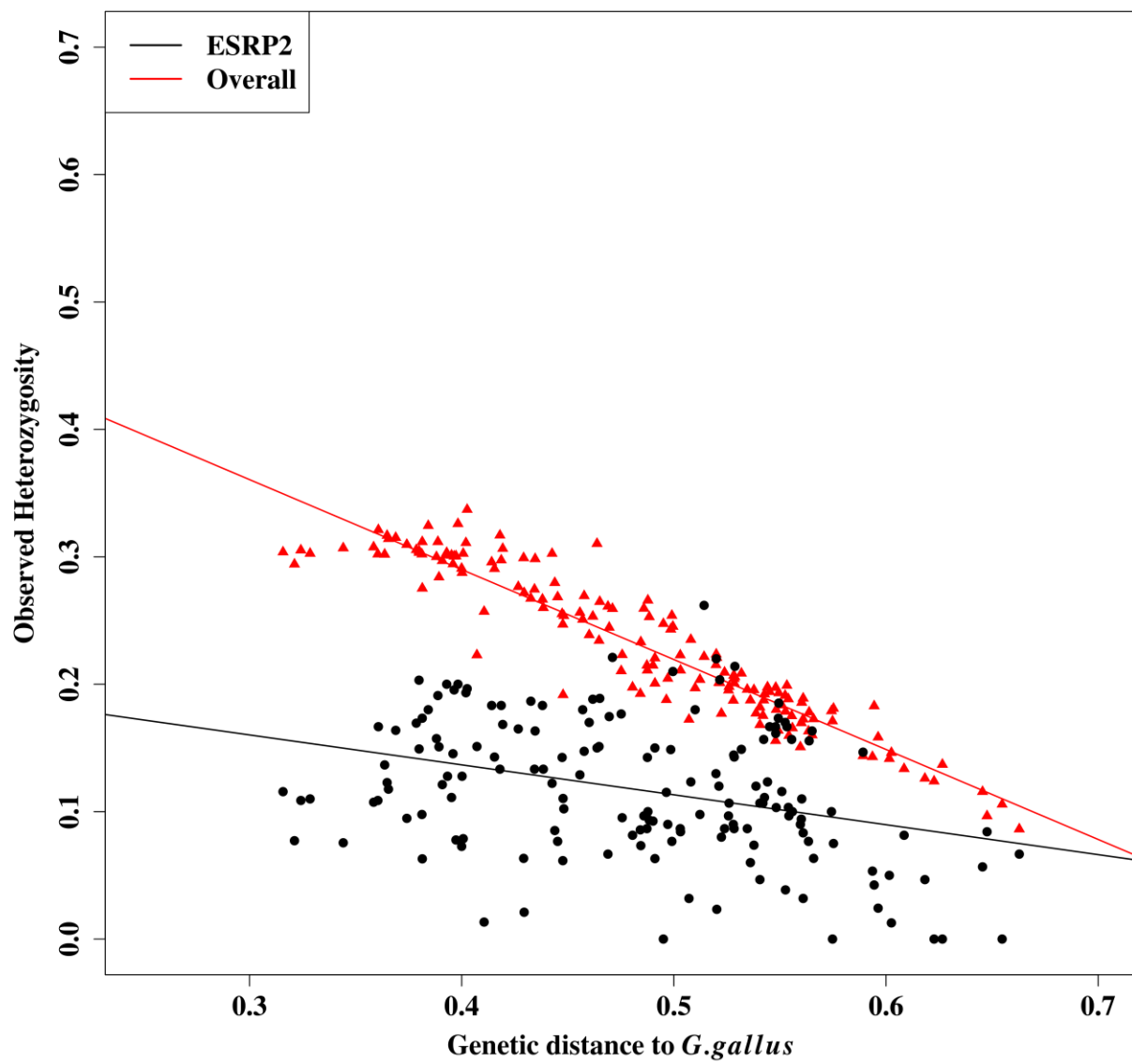

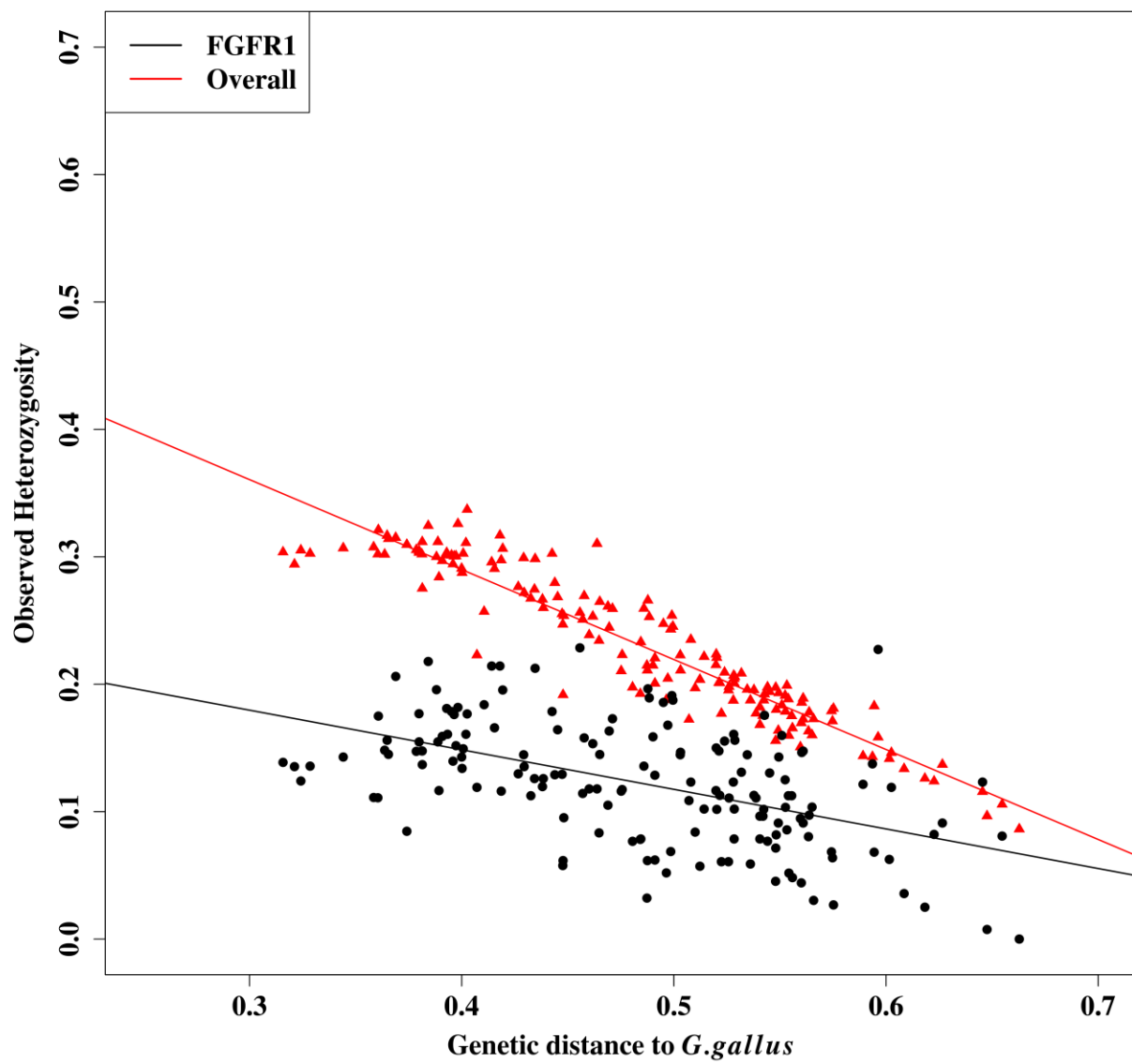

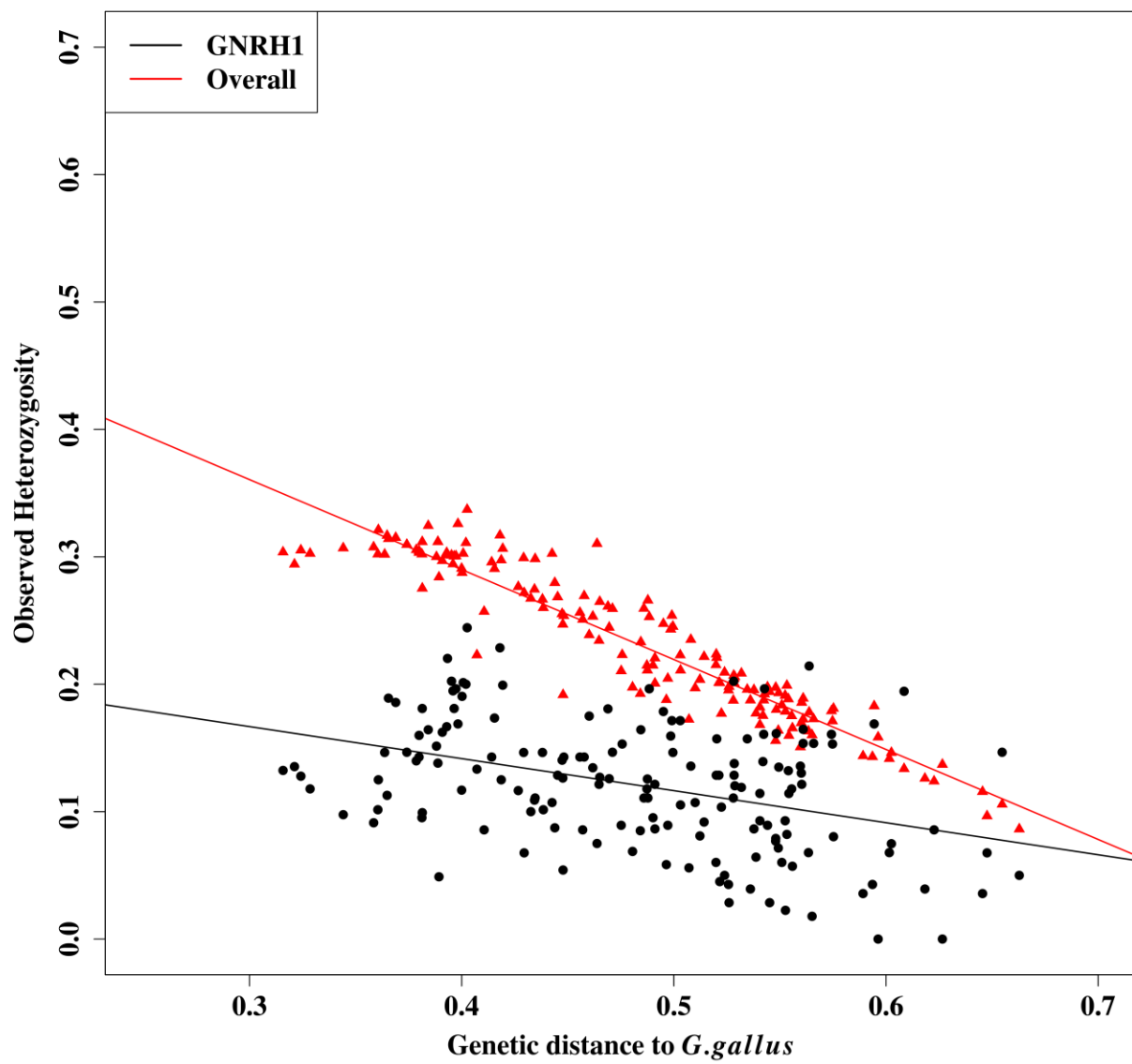

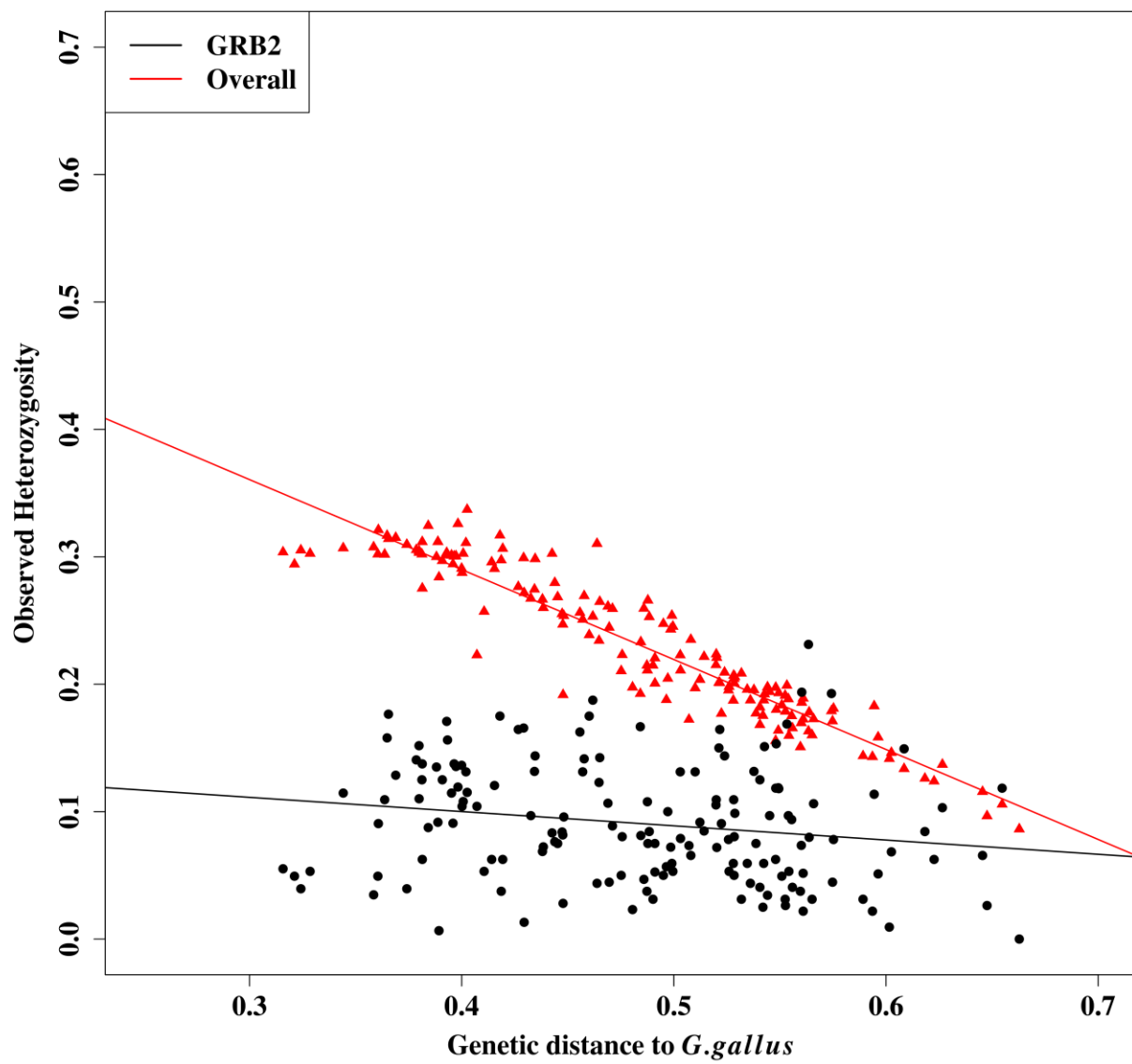

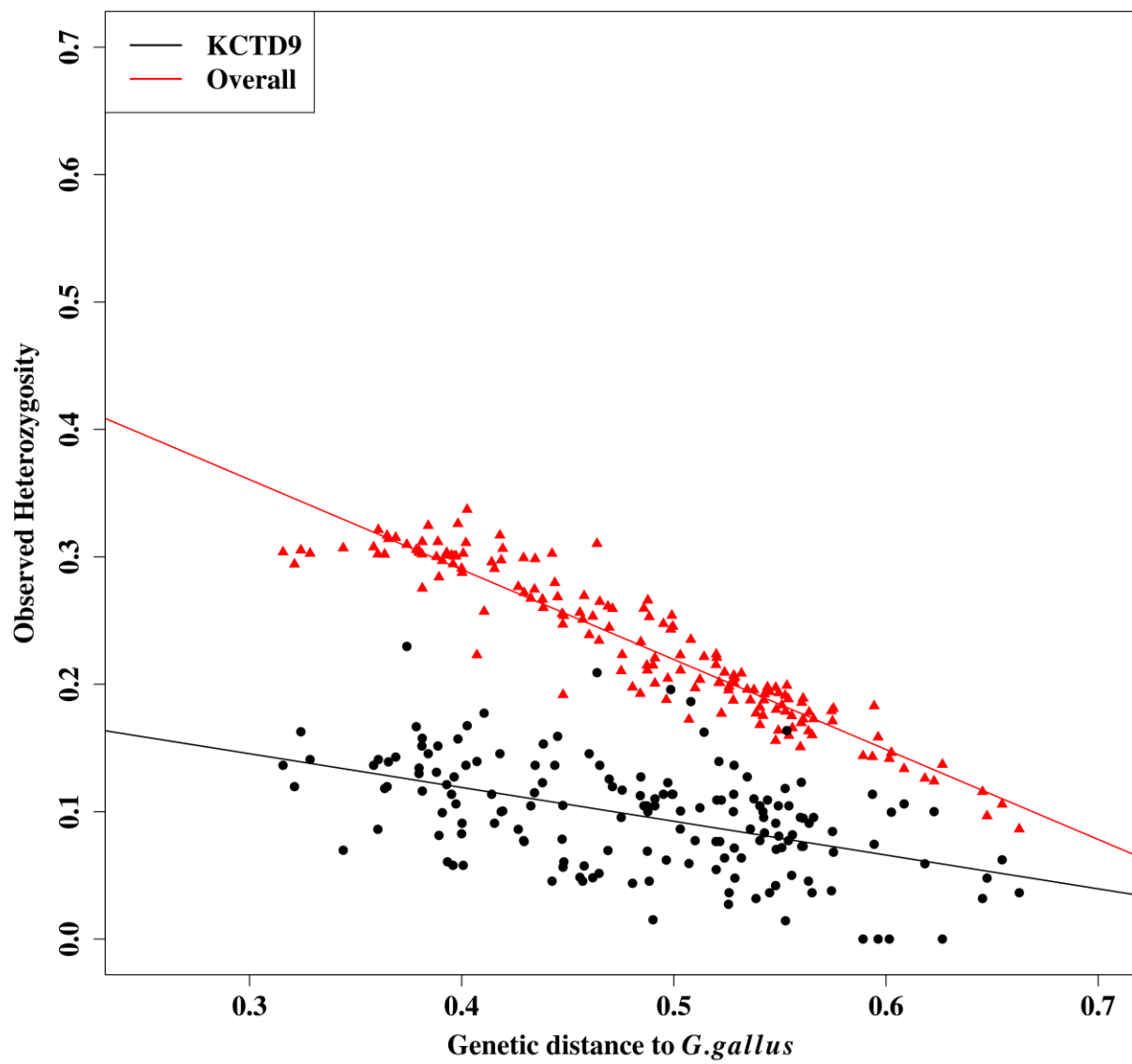

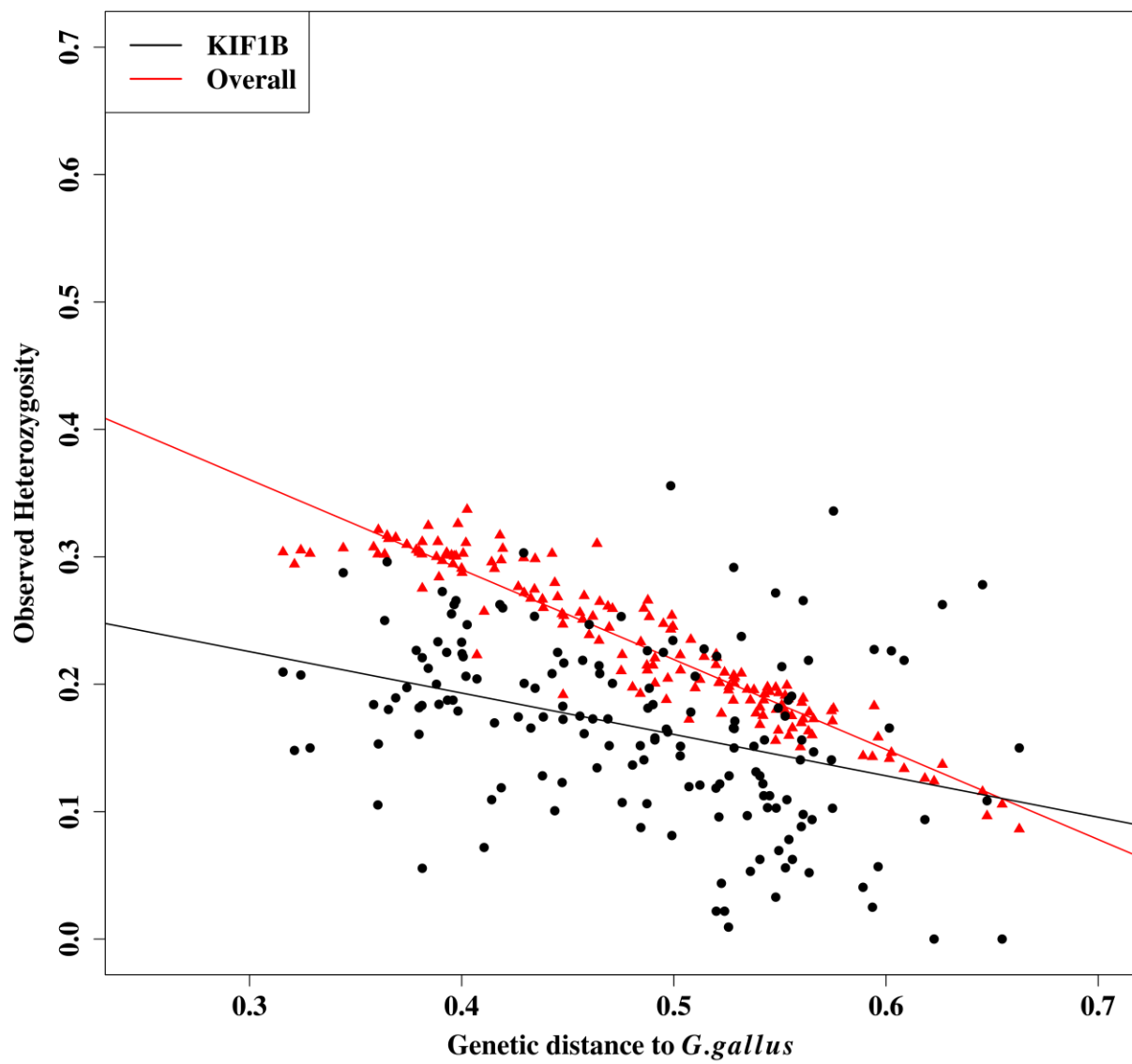

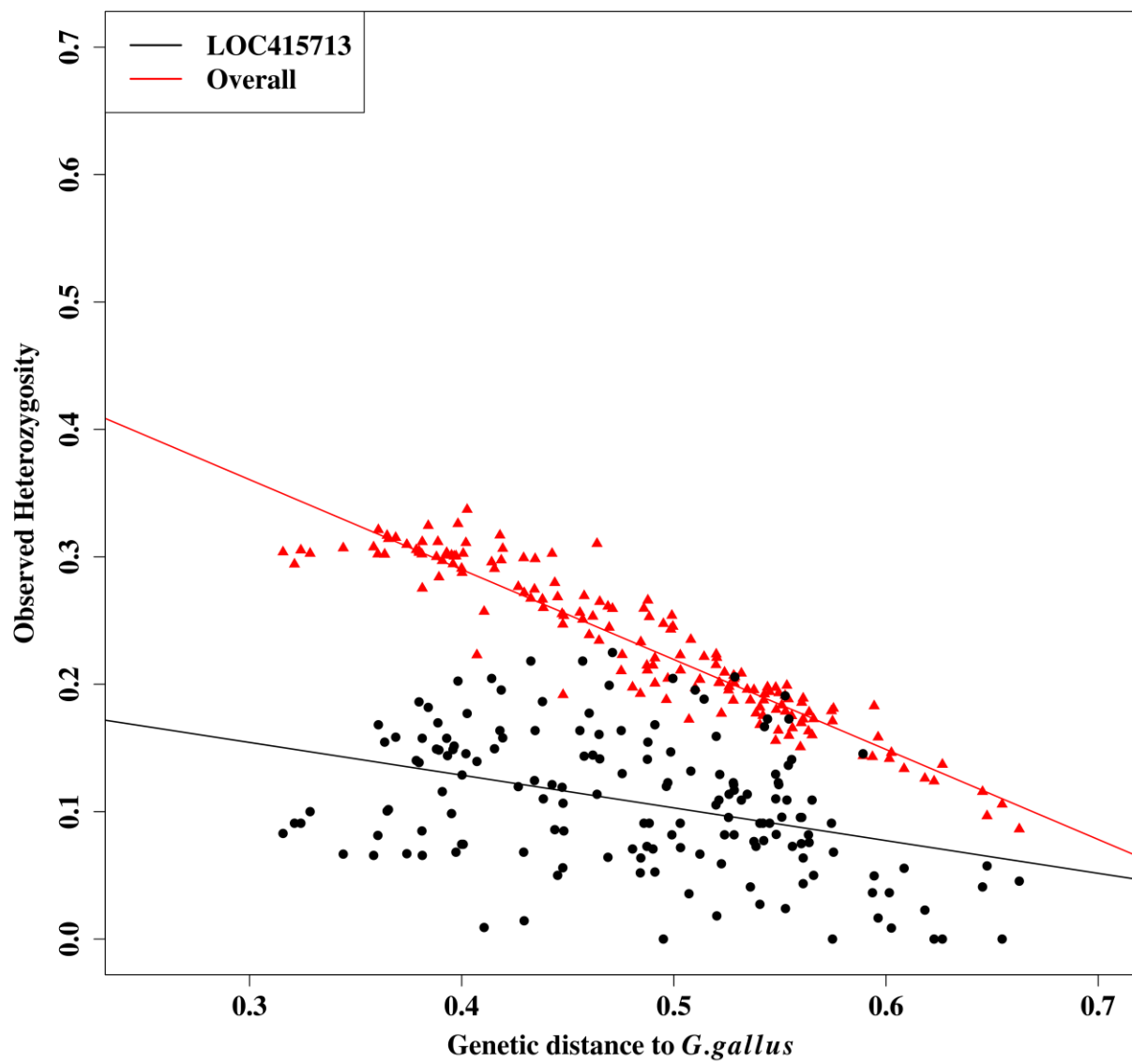

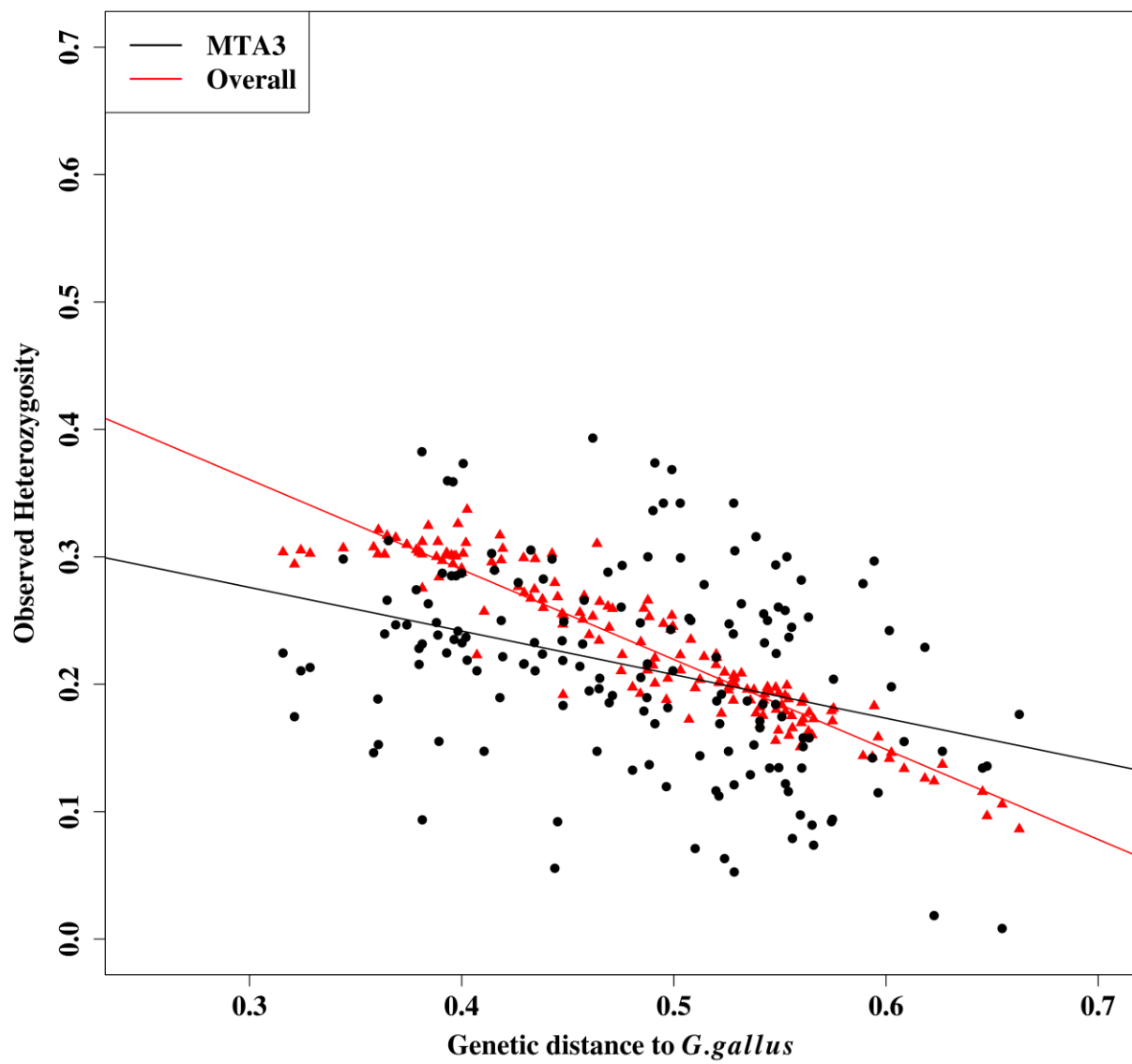

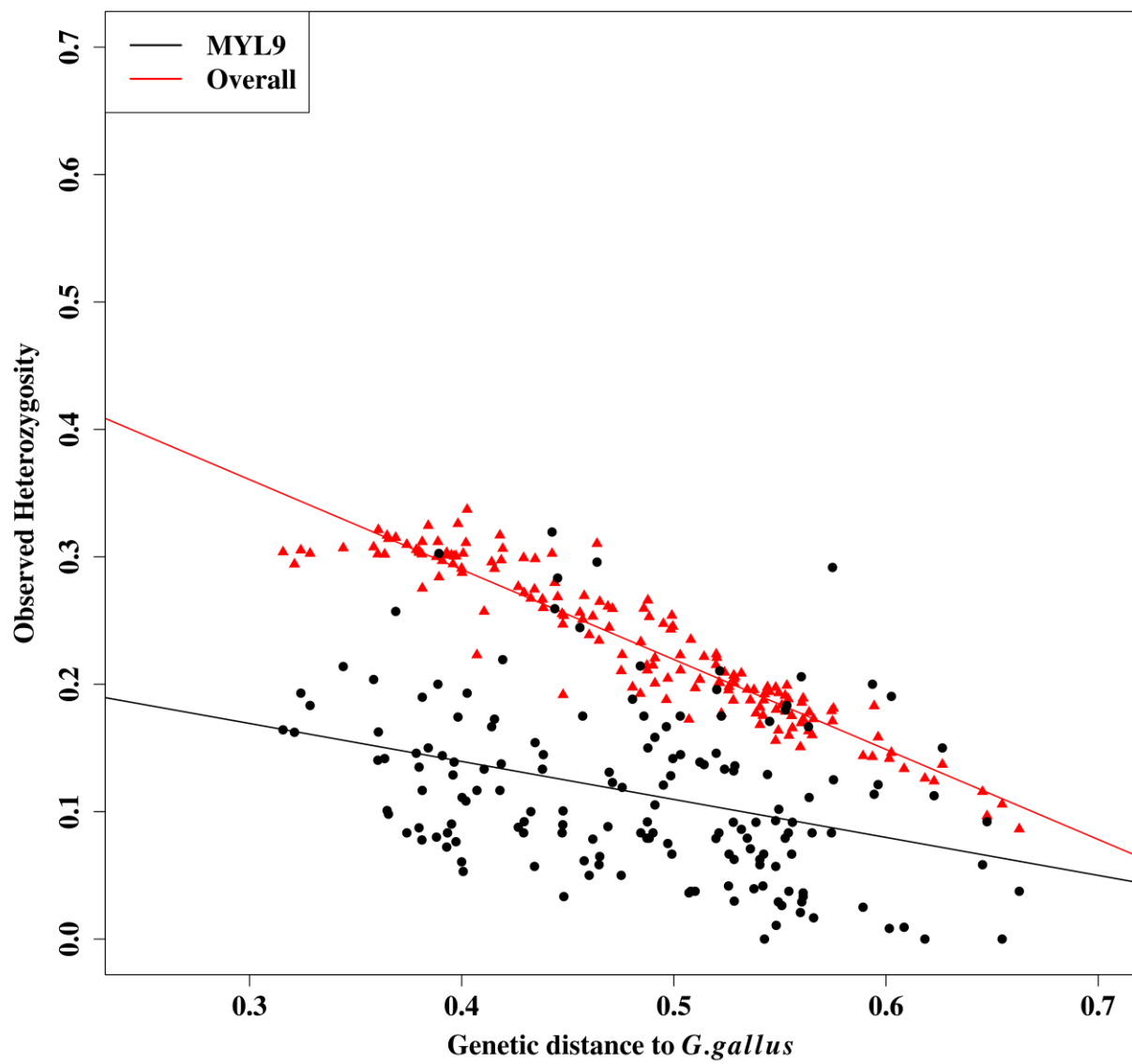

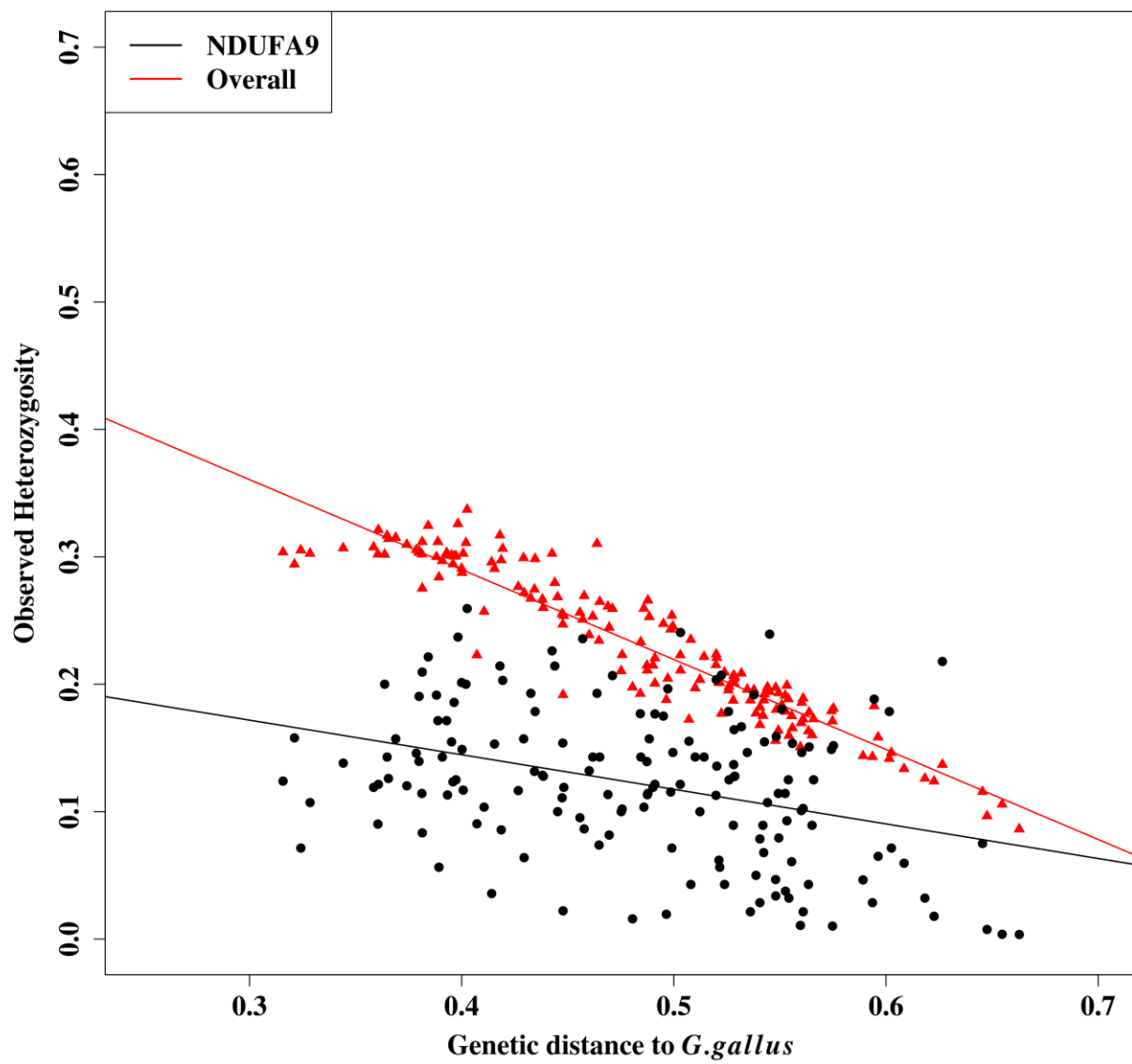

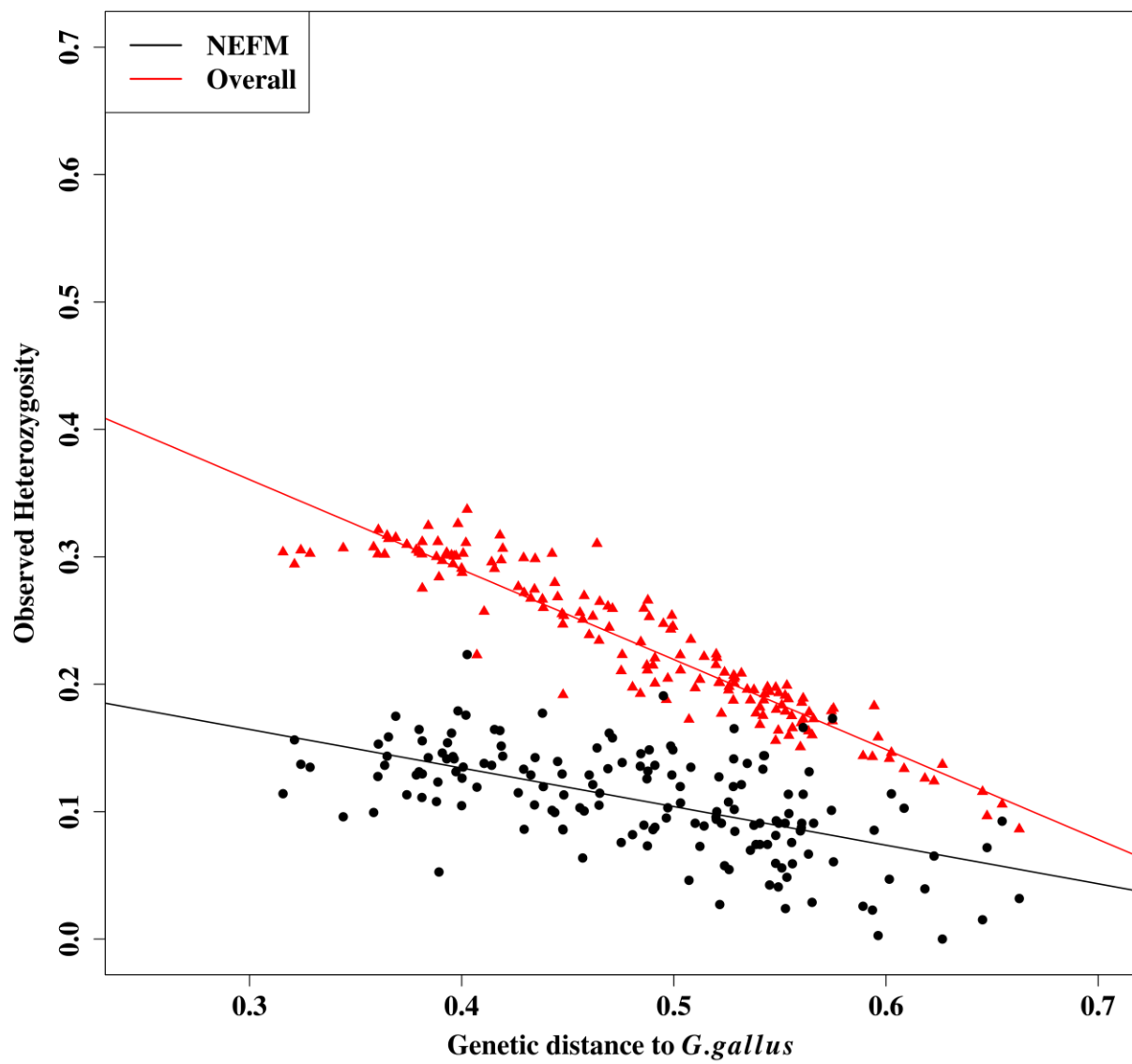

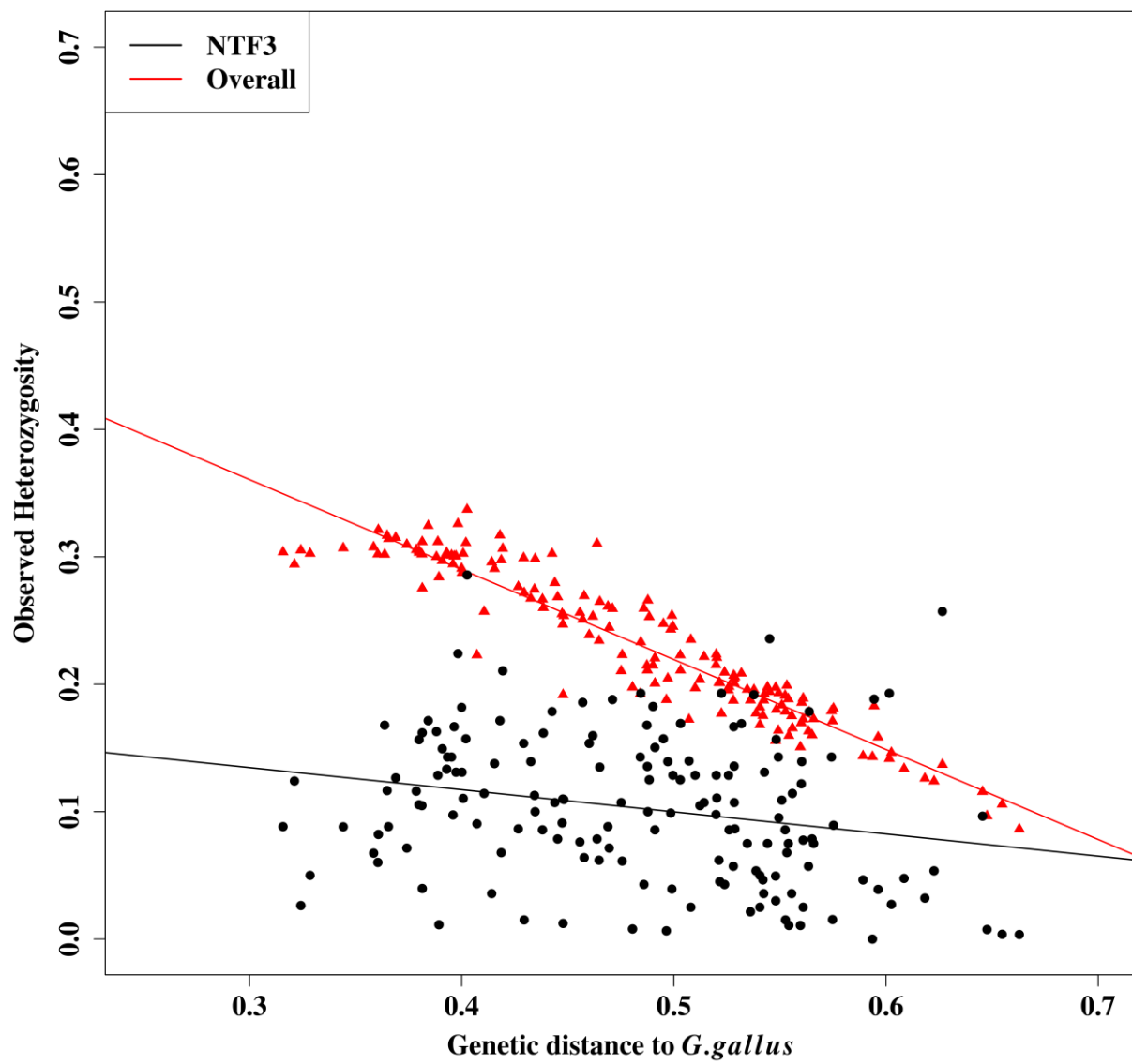

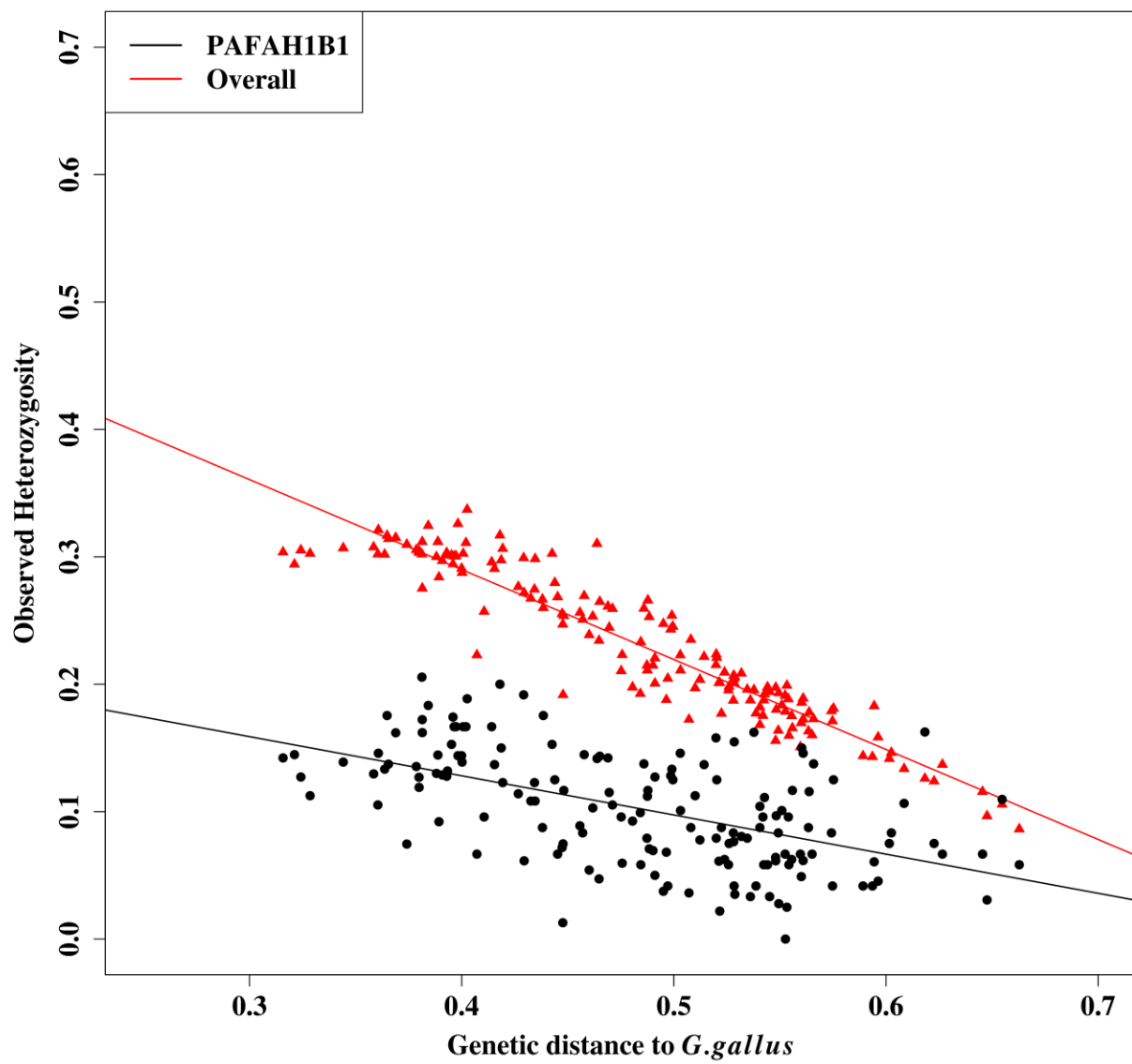

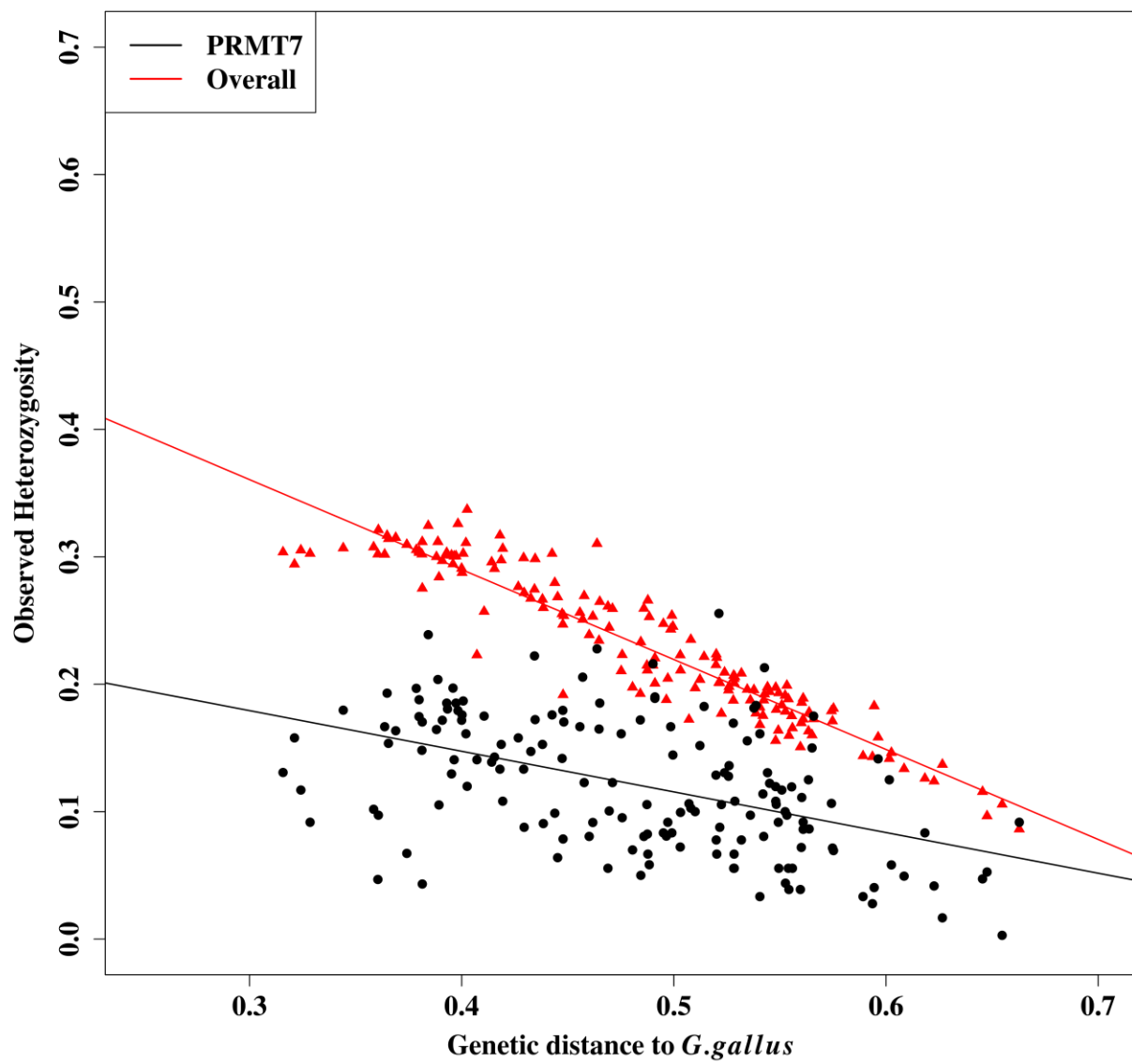

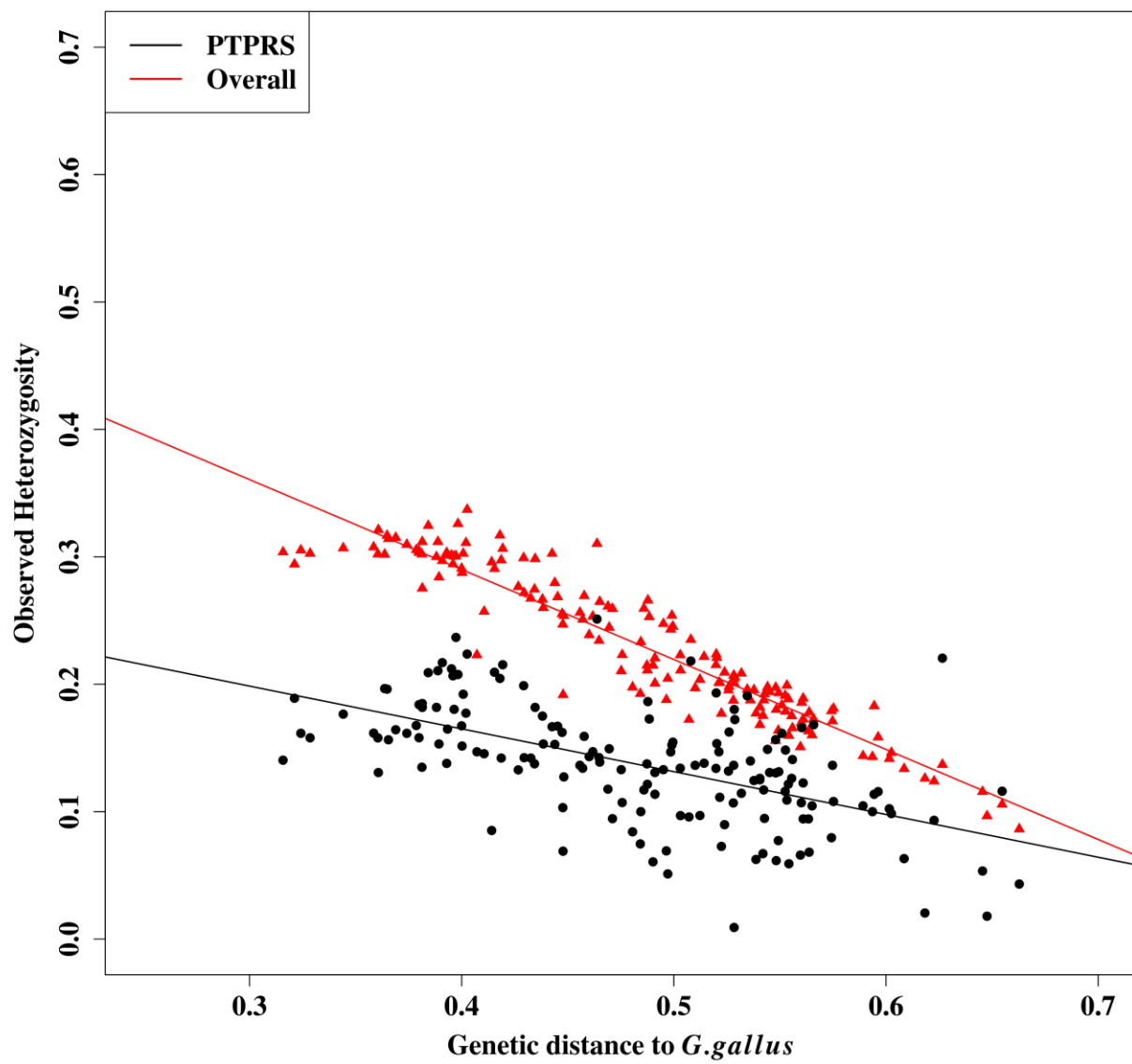

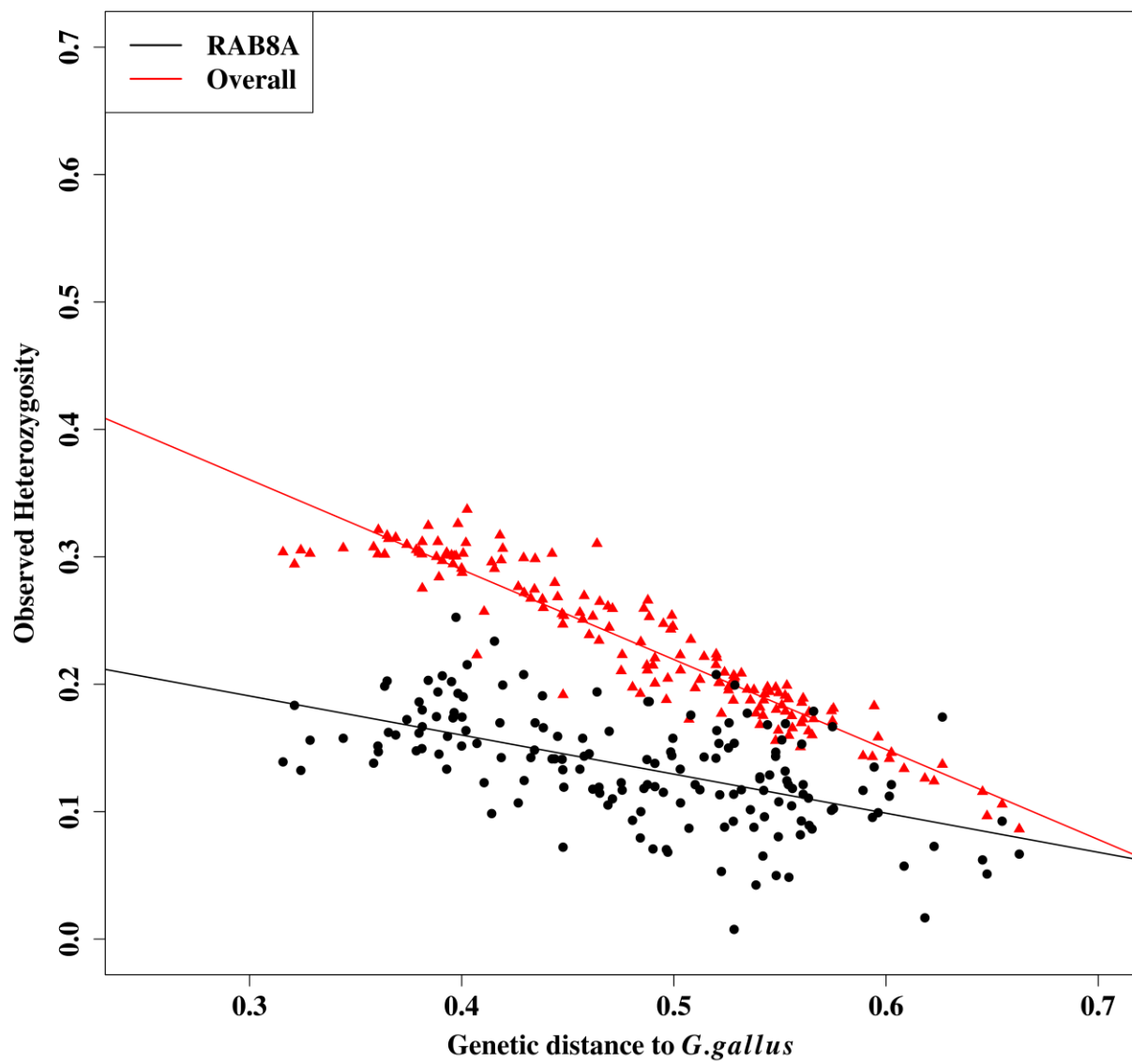

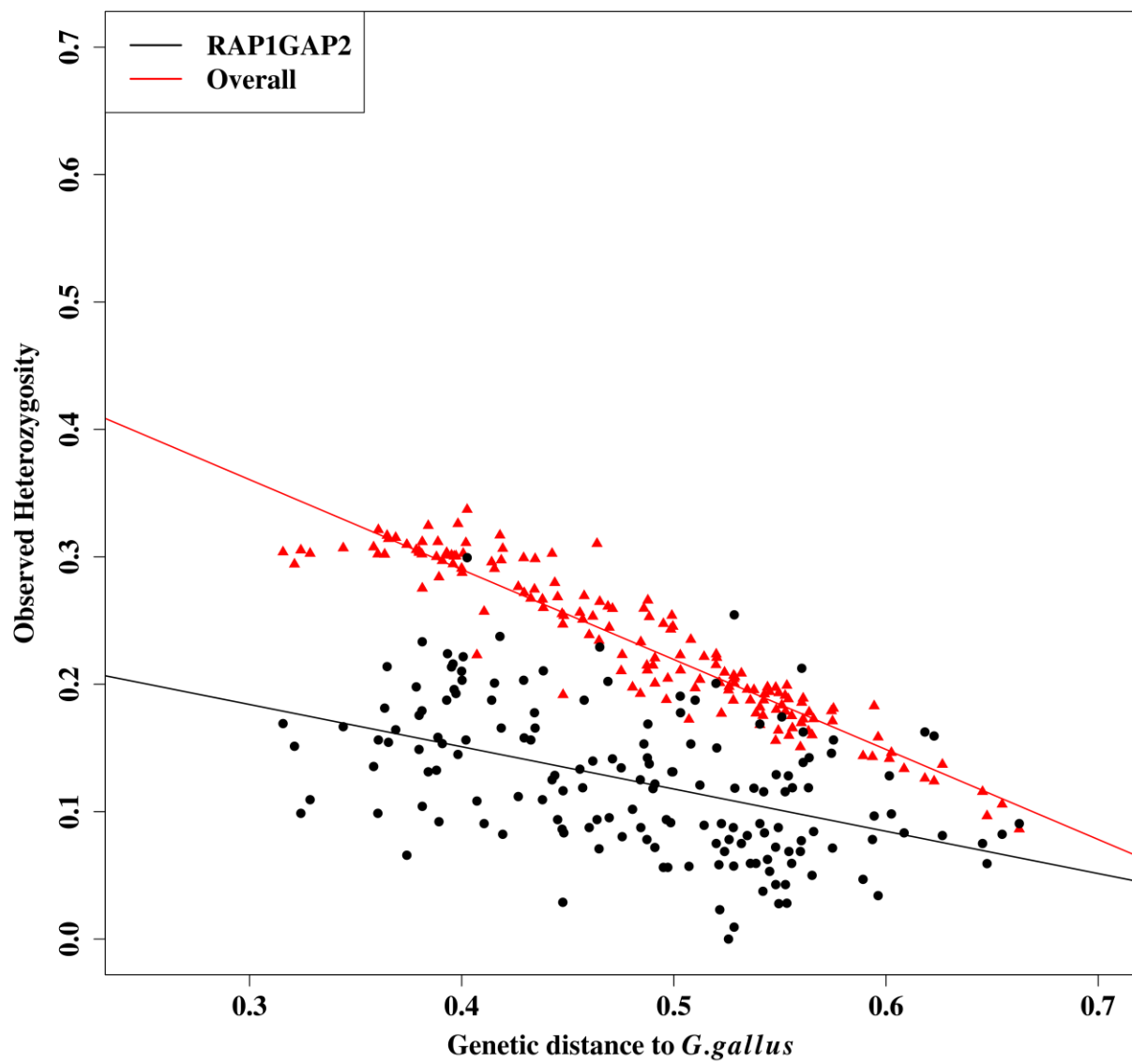

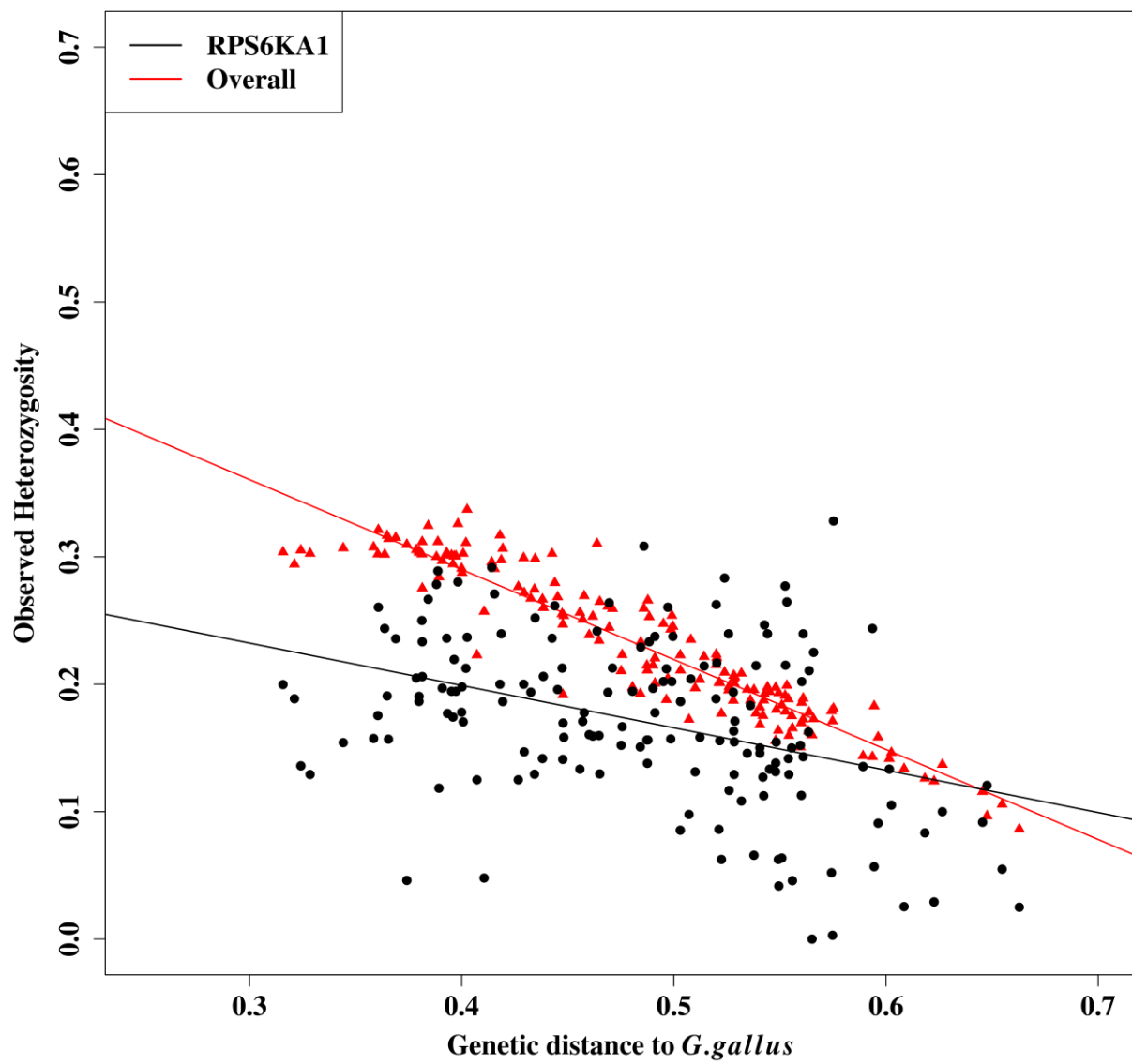

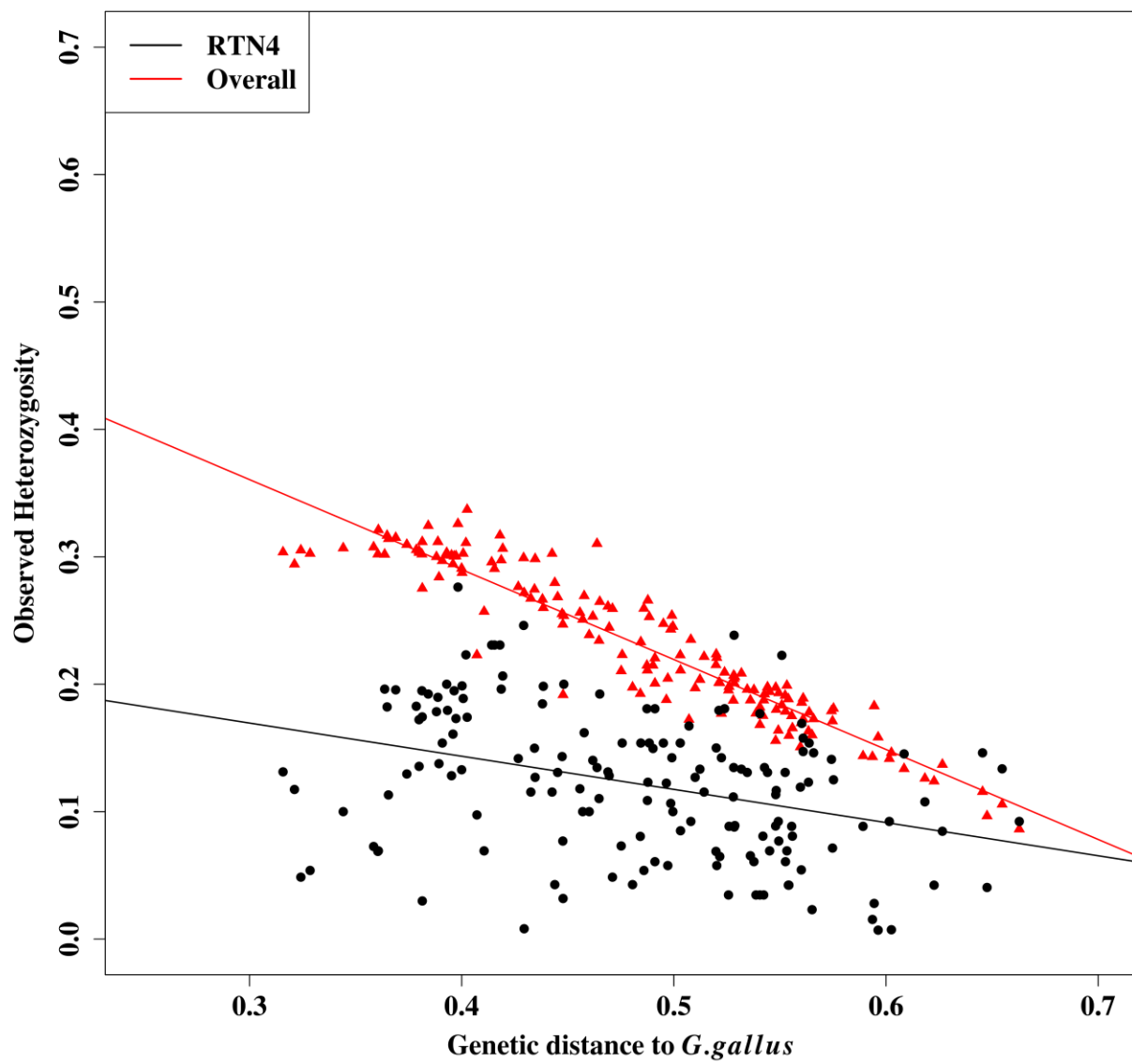

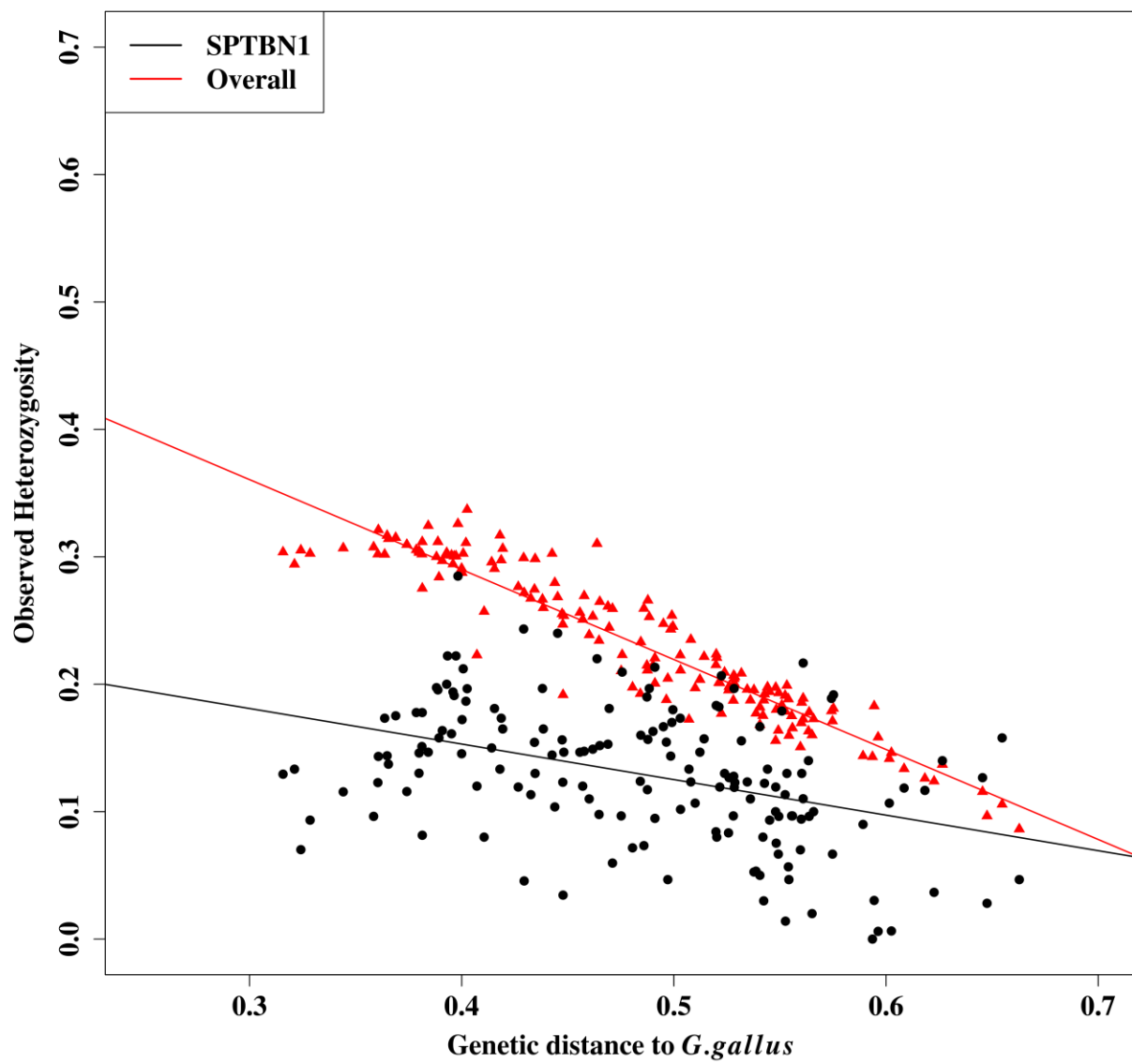

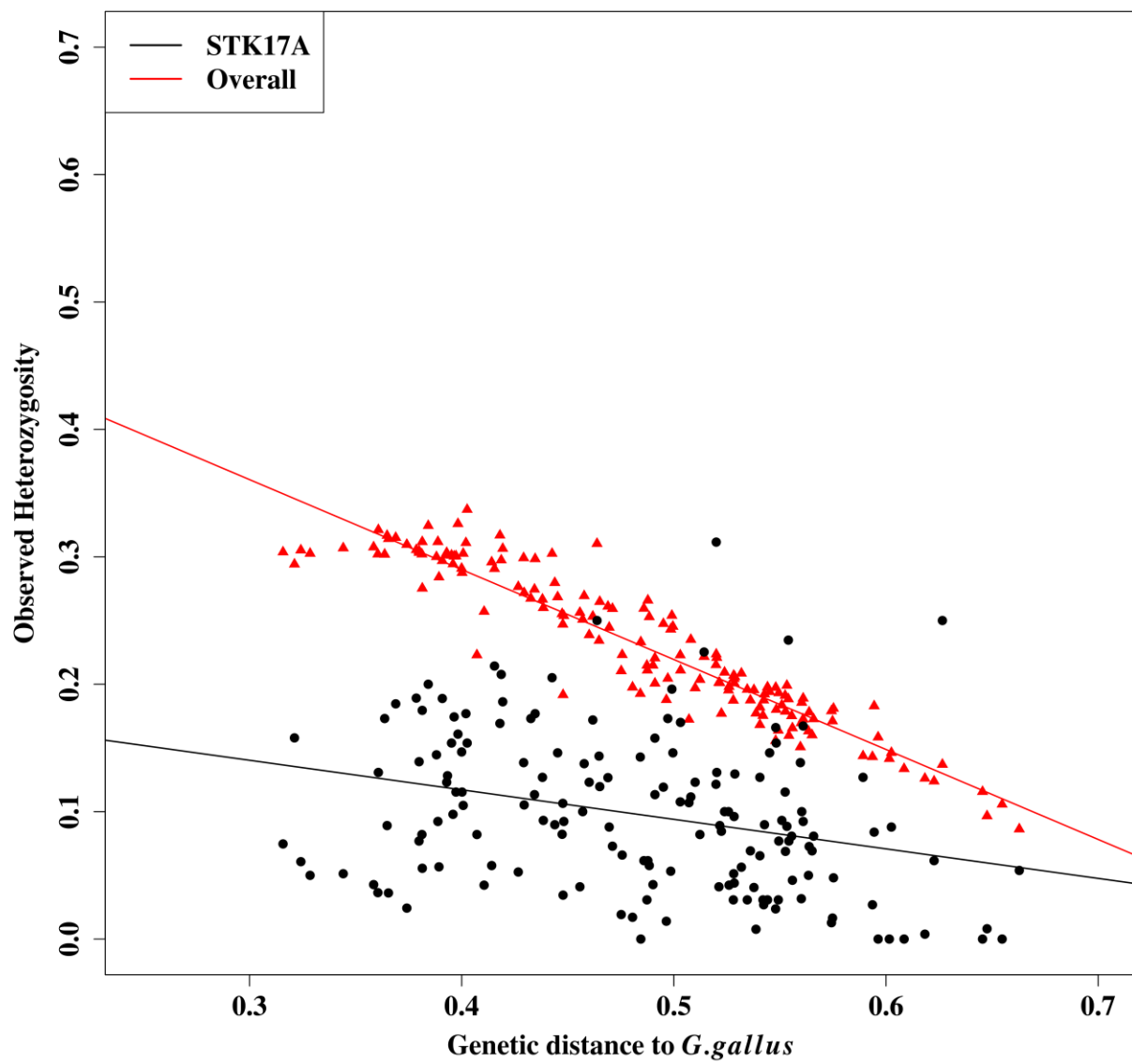

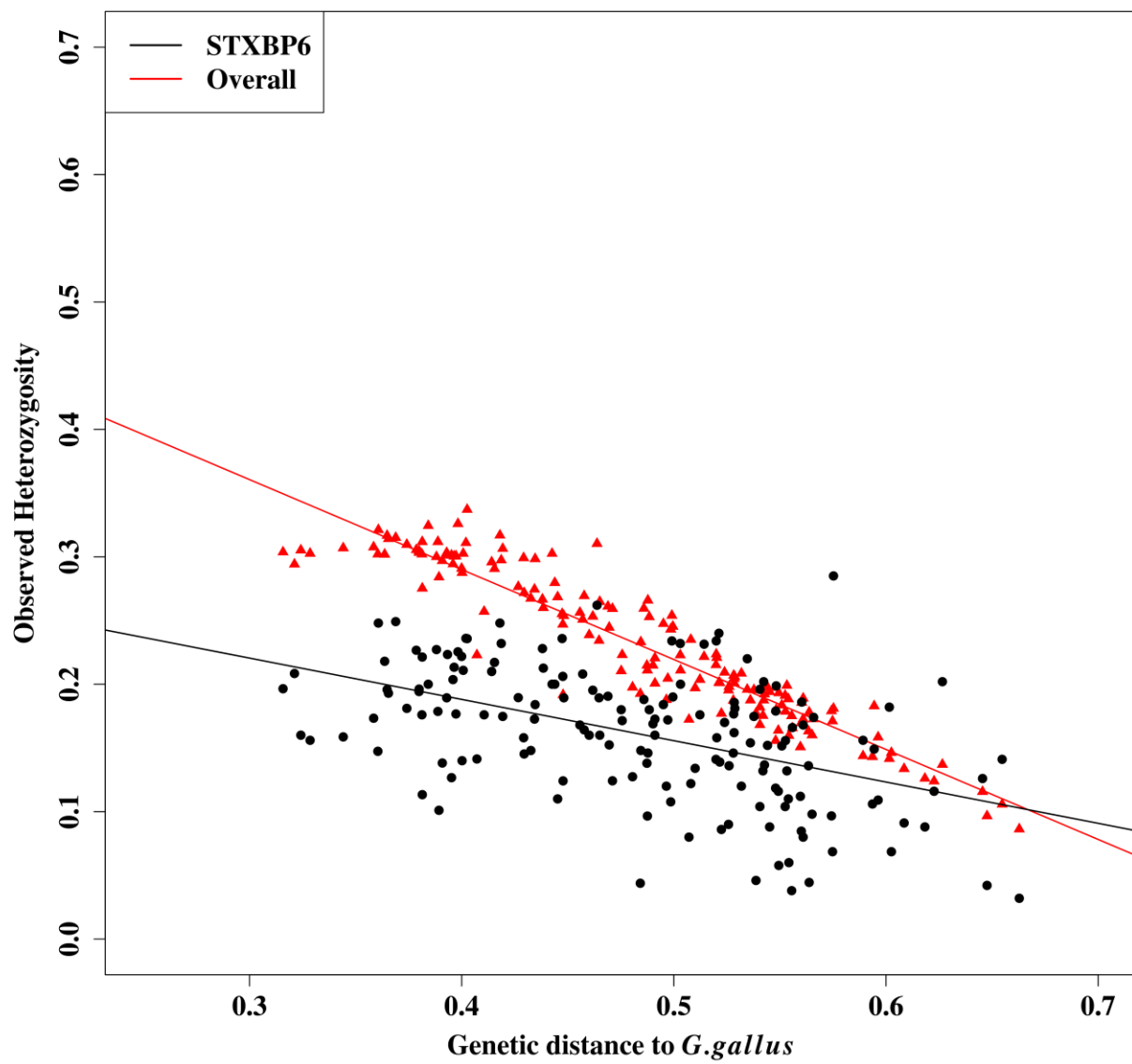

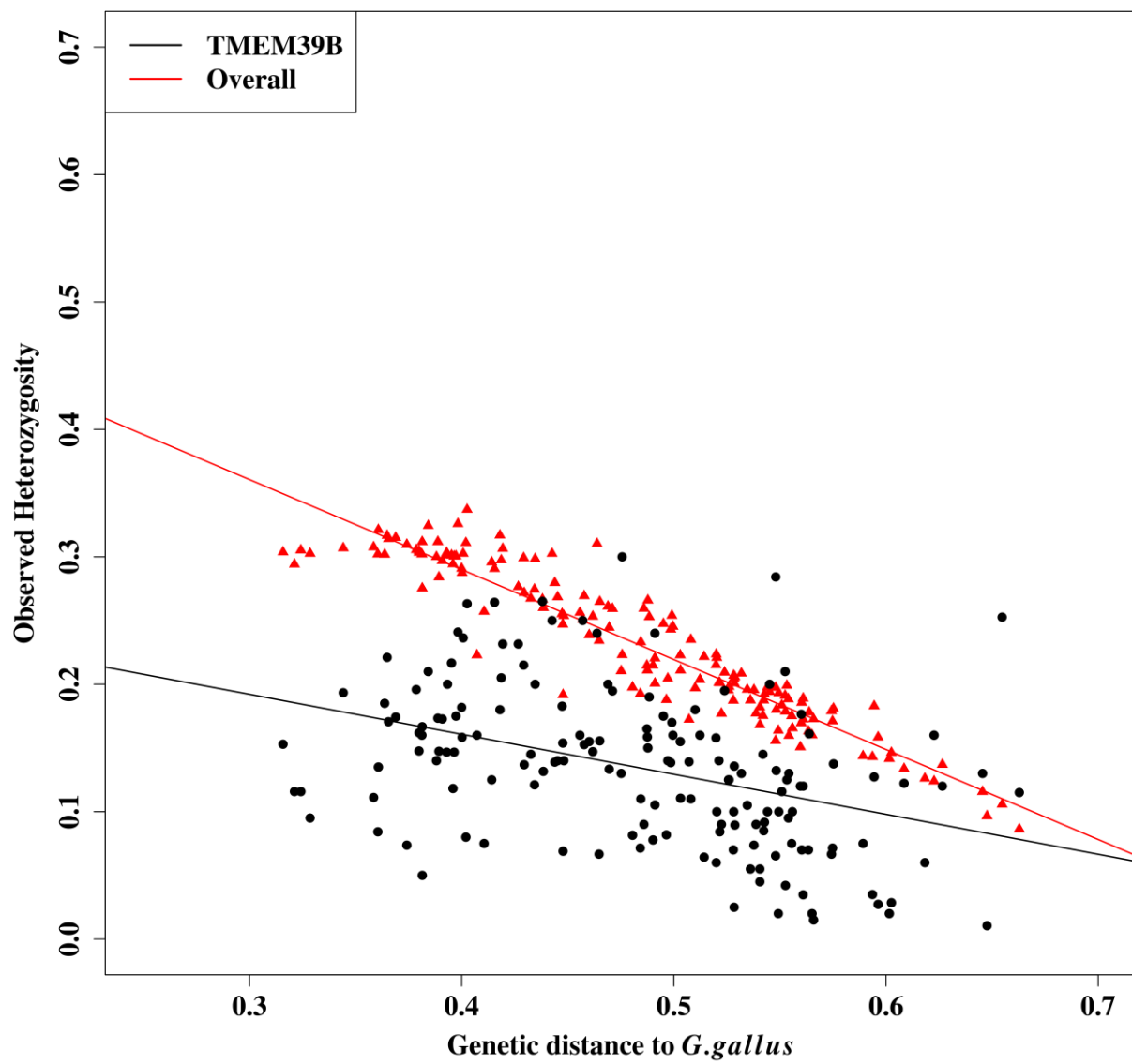

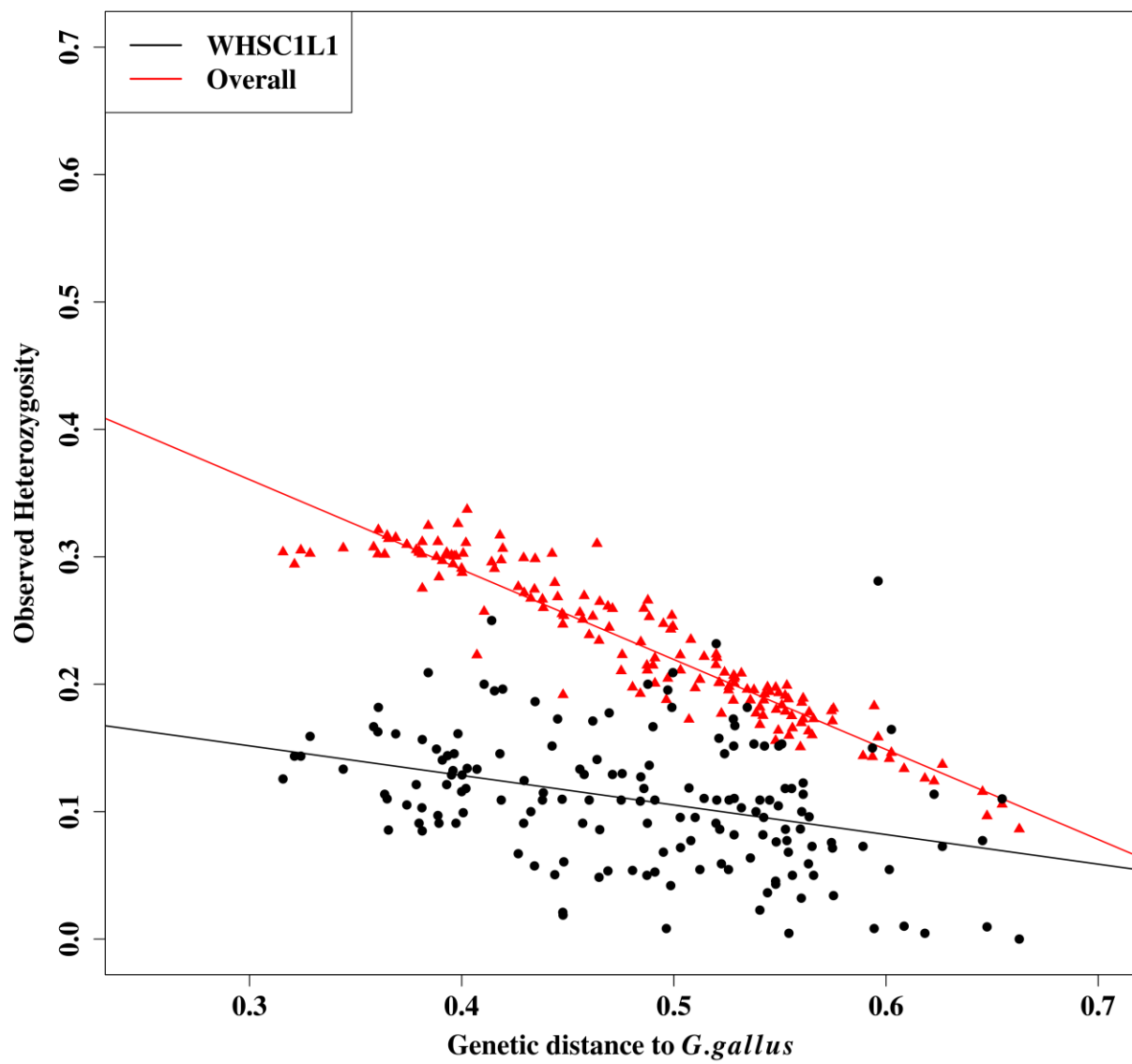

Supplement: Supplementary file 5 — Additional file 5. Relationships between observed heterozygosity and genetic distance to G. gallus for genes in the lowest 5% slope range. [file 12711_2021_628_MOESM5_ESM.pdf]
